# New Analogs of Polyamine Toxins from Spiders and Wasps: Liquid Phase Fragment Synthesis and Evaluation of Antiproliferative Activity

**DOI:** 10.3390/molecules27020447

**Published:** 2022-01-10

**Authors:** Christos Vassileiou, Stefania Kalantzi, Eleanna Vachlioti, Constantinos M. Athanassopoulos, Christos Koutsakis, Zoi Piperigkou, Nikos Karamanos, Theodora Stivarou, Peggy Lymberi, Konstantinos Avgoustakis, Dionissios Papaioannou

**Affiliations:** 1 Laboratory of Synthetic Organic Chemistry, Department of Chemistry, University of Patras, 265 04 Patras, Greece; c.vassileiou@gmail.com (C.V.); s.kalantzi8@gmail.com (S.K.); eleannavah@yahoo.gr (E.V.); 2 Biochemical Analysis & Matrix Pathobiology Research Group, Laboratory of Biochemistry, Department of Chemistry, University of Patras, 265 04 Patras, Greece; ckoutsakis@upatras.gr (C.K.); zoipip@upatras.gr (Z.P.); 3 Foundation for Research and Technology-Hellas (FORTH)/Institute of Chemical Engineering Sciences (ICE-HT), 261 10 Patras, Greece; 4 Immunology Laboratory, Department of Immunology, Hellenic Pasteur Institute, 127 Vas. Sofias Avenue, Ampelokipi, 115 21 Athens, Greece; tstivarou@pasteur.gr (T.S.); plymberi@pasteur.gr (P.L.); 5 Department of Pharmacy, University of Patras, 265 04 Patras, Greece; avgoust@upatras.gr

**Keywords:** polyamine toxins, polyamines, lipophilic head groups, hydroxy amino acids, (indol-3-yl)acetic acid, propargylglycine, 4-(3-trifluoromethyldiazirin-3-yl)benzoic acid, antiproliferative activity, breast cancer cells, cytotoxicity

## Abstract

Polyamine toxins (PATs) are conjugates of polyamines (PAs) with lipophilic carboxylic acids, which have been recently shown to present antiproliferative activity. Ten analogs of the spider PATs **Agel 416**, **HO-416b**, and JSTX-3 and the wasp PAT PhTX-433 were synthesized with changes in the lipophilic head group and/or the PA chain, and their antiproliferative activity was evaluated on MCF-7 and MDA-MB-231 breast cancer cells, using **Agel 416** and **HO-416b** as reference compounds. All five analogs of PhTX-433 were of very low activity on both cell lines, whereas the two analogs of JSTX-3 were highly active only on the MCF-7 cell line with IC_50_ values of 2.63–2.81 μΜ. Of the remaining three **Agel 416** or **HO-416b** analogs, only the one with the spermidine chain was highly active on both cells with IC_50_ values of 3.15–12.6 μM. The two most potent compounds in this series, **Agel 416** and **HO-416b**, with IC_50_ values of 0.09–3.98 μΜ for both cell lines, were found to have a very weak cytotoxic effect on the MCF-12A normal breast cells. The present study points out that the structure of both the head group and the PA chain determine the strength of the antiproliferative activity of PATs and their selectivity towards different cells.

## 1. Introduction

**Figure 1:**
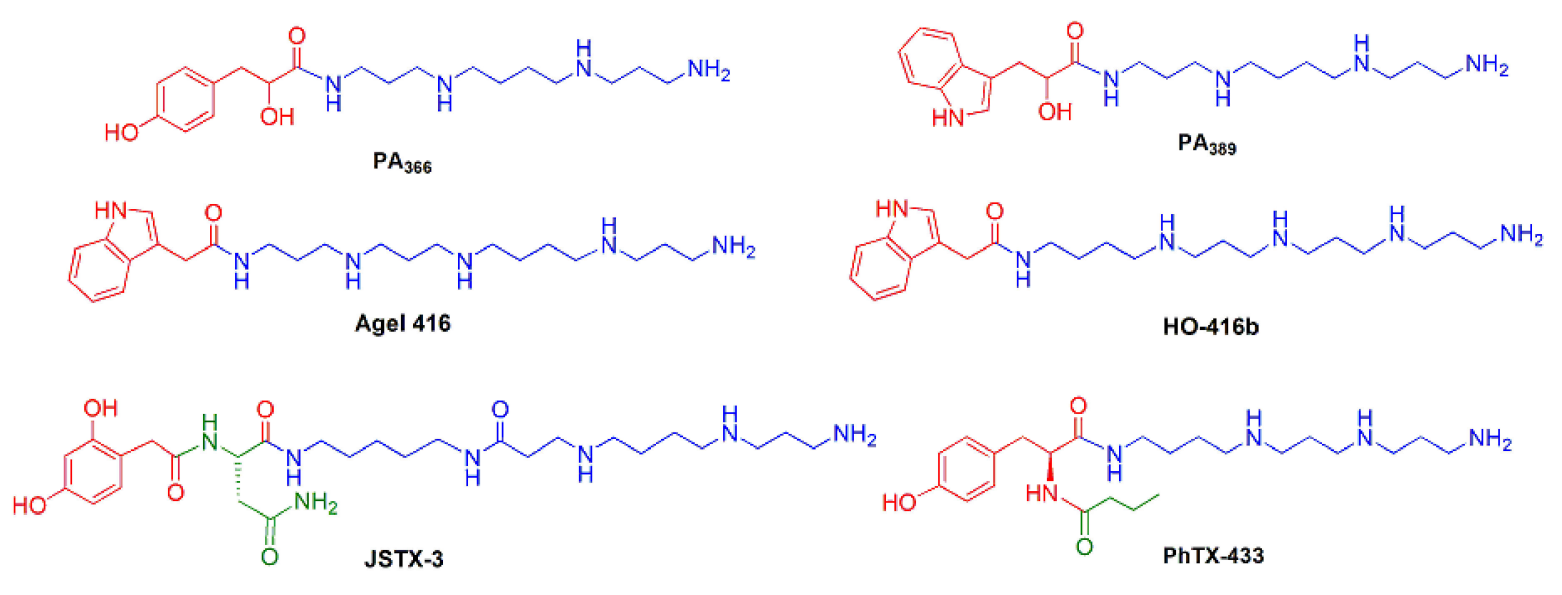
Structures of natural PATs from spider and wasp venoms related to the present work.

Polyamine toxins (PATs) are conjugates of polyamines (PAs) with lipophilic acids isolated from spider or wasp venoms (Figure 1). PATs are primarily investigated for their ability to block ionotropic glutamate receptors (iGluR) and thus constitute interesting drug targets for neurological and psychiatric disorders [1,2,3,4]. Recent studies showed that the PATs are also endowed with interesting antiproliferative activity, which seems to depend on the structure of the lipophilic head group (PATs PA_366_ and PA_389_ from spider venom), with PA_366_ being significantly more active than PA_389_ in MCF-7 breast cancer cells. Both PATs incorporate the spermine (Spm, a 3-4-3 PA) chain and either a 4-hydroxyphenyl (PA_366_) or 1*H*-indol-3-yl subgroup (PA_389_) in the head group [5]. Potential protein targets involved in tumor development and breast cancer were identified for these PATs [5]. On the other hand, the antiproliferative activity seems to depend on the structure of the PA chain (PATs **Agel 416** and **HO-416b** also from spider venom), with PAT **HO-416b** being *ca* six times more active than PAT **Agel 416** in the estrogen receptor alpha (ERα)-positive MCF-7 breast cancer cell line with low metastatic potential, but slightly less active in the ERβ-positive MDA-MB-231 highly invasive breast cancer cell line [6]. PATs **Agel 416** and **HO-416b** differ only in the arrangement of their amino functions in the PA chain, the former being a 3-3-4-3 PA and the latter a 4-3-3-3 PA. It should be noted that a plethora of PA analogs with changes in the basic skeleton of naturally occurring PAs, suitable for SAR studies, has been synthesized and evaluated for their ability to inhibit the proliferation of cancer cells. These studies showed that the antiproliferative activity of this class of compounds depends on the number of the nitrogen atoms in the chain, the distance between the nitrogen atoms, the nature of the terminal alkyl substituents, and the charge these molecules can bear at physiological pH [2,7,8,9].

**Figure 2:**
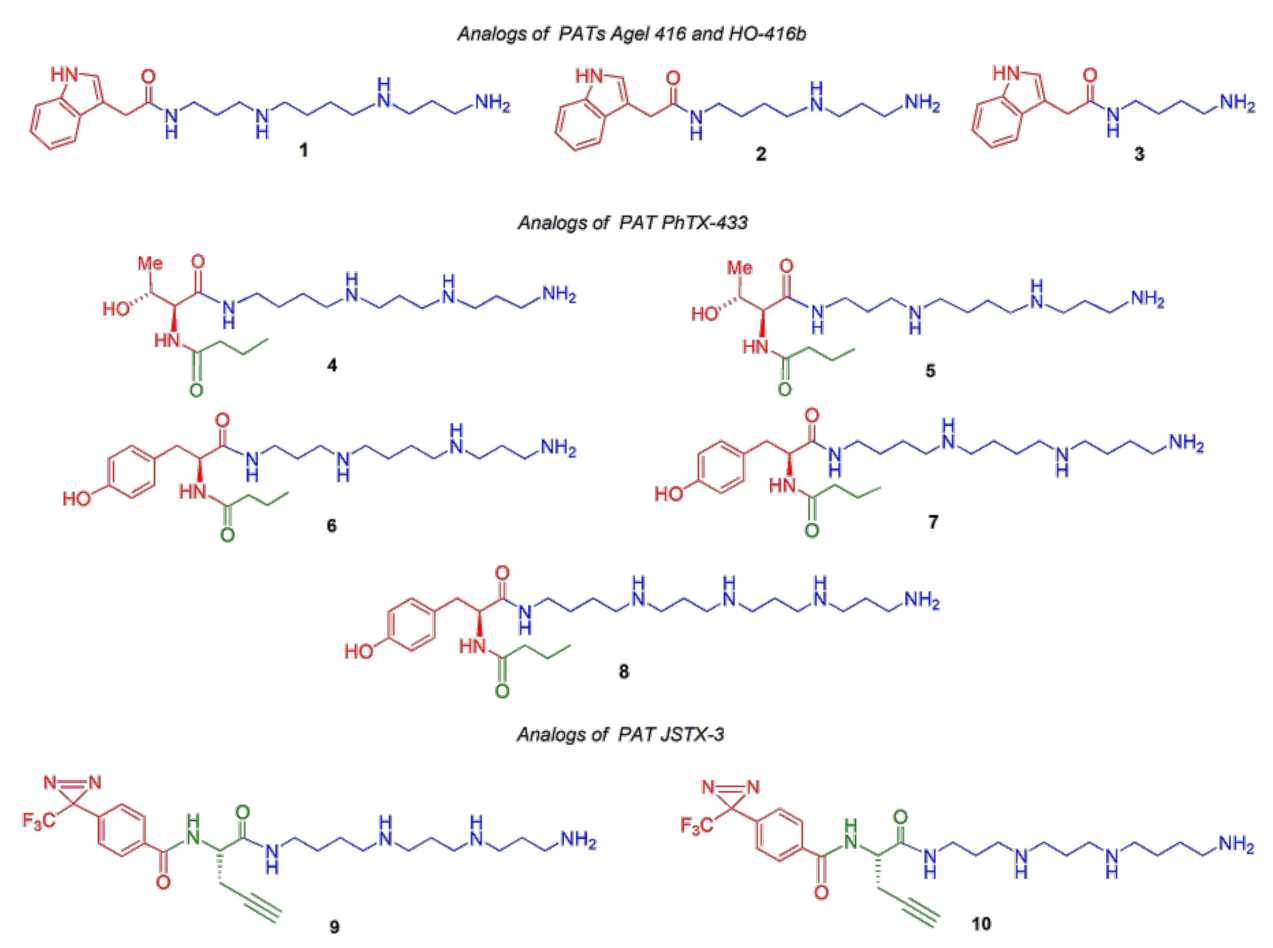
Structures of synthesized PAT analogs in the context of the present study.

We now wish to report our results on the synthesis and antiproliferative activity in the two breast cancer cell lines MCF-7 and MDA-MB-231, of (a) shorter PA chain analogs (**1**–**3**, Figure 2) of PATs **Agel 416** and **HO-416b**, (b) analogs of PhTX-433 with changes in the lipophilic head group (compounds **4** and **5**) or the PA chain (compounds **6**–**8**), and (c) analogs (compounds **9** and **10**) of PAT JSTX-3, isolated from spider venoms, incorporating photoactivatable and clickable moieties [10] in the lipophilic part and the naturally occurring thermospermine (Tsm, a 3-3-4 PA) as the PA chain in the place of the longer PA chain of JSTX-3. With analogs **1**–**3**, we aimed to study the effect of the PA chain length and the number of the amino functions on the established strong antiproliferative effect of PATs **Agel 416** and **HO-416b**, whereas with analogs **4**–**8**, we wished to study the effect of the structures of the lipophilic head group and of the PA chain on the potential antiproliferative effect of PAT PhTX-433. It should be noted that the synthetic analog **6**, commonly abbreviated as PhTX-343, has been used as a reference compound for SAR studies instead of the natural PAT PhTX-433 [11]. Finally, bisamide analogs **9** and **10**, bearing in the lipophilic head group a combination of an aromatic carboxylic acid with a photo-labeling moiety (a diazirine ring) and an amino acid with a clickable moiety (a terminal alkyne function), were designed in such a way that in the case they also presented comparable antiproliferative activity with the natural PATs or analogs thereof, they could be used at a later stage for the identification of the potential target(s) of their antiproliferative activity. They could also provide evidence of the possible effect of the site of attachment of the lipophilic head group in an asymmetric PA molecule, such as Tsm, on the antiproliferative activity. 

## 2. Results and Discussion

### 2.1. Chemistry

A variety of chemical strategies have been developed, both in liquid and solid phases, for the synthesis of PAs, analogs, and conjugates thereof [2,12]. Quite recently, a general approach for the liquid-phase fragment synthesis of orthogonally protected PAs and their application to the synthesis of the PATs **Agel 416** and **HO-416b** has been reported [13]. This particular methodology, with minor modifications, was also applied for the synthesis of the PAT analogs **1**–**10** described in the present work, which involved the assembly of the required PA skeleta orthogonally protected at their amino functions so that selective deprotection of the desired terminal amino functionality would allow the attachment of the appropriate lipophilic head group on that particular position of the PA chain.

#### 2.1.1. The Selectively Protected PA Skeleta

**Figure 3:**
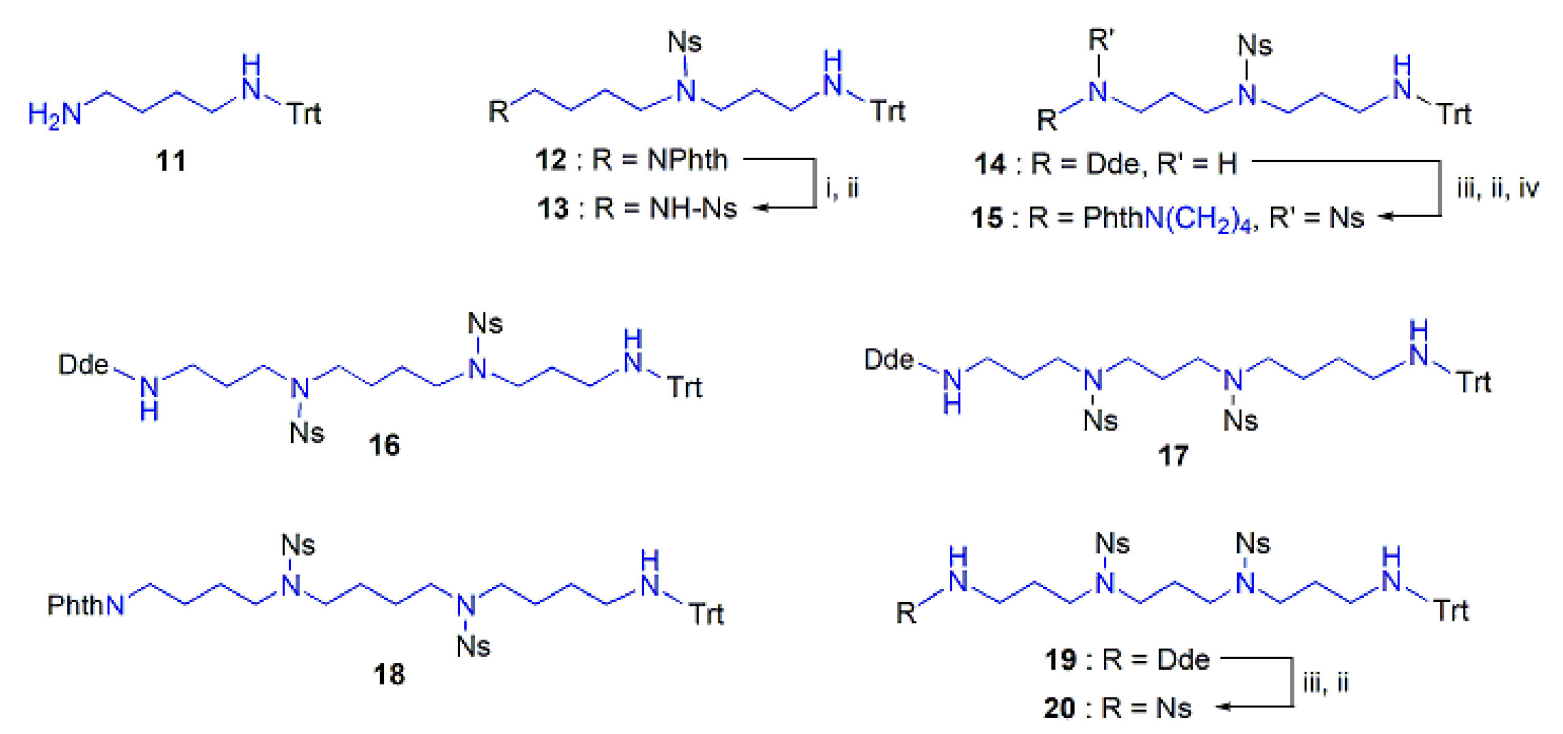
Structures of selectively protected PAs. *Reagents and conditions*: (i) H_2_NNH_2_^.^H_2_O, EtOH, 78 °C, 2 h, 87%; (ii) NsCl, Et_3_N, DCM, 0 °C, 2 h then 25 °C, 2 h, 62–81%; (iii) 2% Et_2_NH in DMF, 25 °C, 30 min, 72–94%; (iv) PhthN(CH_2_)_4_Br, K_2_CO_3_, DMF, 60 °C, 2.5 h, 80%.

Key intermediates for the assembly of conjugates **1**–**10** are the selectively protected PAs **11**–**13**, **15**–**18**, and **20** (Figure 3). From the latter, compounds **11**, **12**, and **16**–**18** were available in our laboratory from a previous project [13] whereas compounds **13**, **15**, and **20** were synthesized as depicted in Figure 3 using compounds **12**, **14**, and **19** as starting materials [13], respectively. In particular, intermediate **13** was prepared from the spermidine (Spd, a 3-4 PA) derivative **12** through a two-step sequence in 54% yield for the two steps [13]. It involved the selective removal of the Phth-protecting group via hydrazinolysis at refluxing EtOH and then nosylation of the deprotected primary amino group with *o*-nitrophenylsulphonyl chloride (nosyl chloride, NsCl) in dichloromethane (DCM) in the presence of Et_3_N.

On the other hand, compound **15** was synthesized from the norspermidine (Nsd, a 3-3 PA) derivative **14** through a three-step sequence, which involved hydrazine-mediated removal of the 1-(4,4-dimethyl-2,6-dioxocyclohexylidene)ethyl (Dde)-protecting group, followed by nosylation of the resulting primary amino function. Finally, alkylation of the nosylated amino functionality with *N*-(4-bromobutyl)phthalimide in the presence of K_2_CO_3_ produced compound **15** in 39% overall yield. The *N*-(4-bromobutyl)phthalimide was obtained in 90% from *N*-(4-hydroxybutyl)phthalimide [13] using the Appel reaction (see Experimental Section). The conversion of the orthogonally protected nonspermine (Nsm, a 3-3-3 PA) derivative **19** to the trinosylated Nsm intermediate **20** was performed in two steps with 76% overall yield, which involved Dde group removal followed by nosylation of the liberated primary amino function [13].

#### 2.1.2. The Lipophilic Head Groups

**Figure 4:**
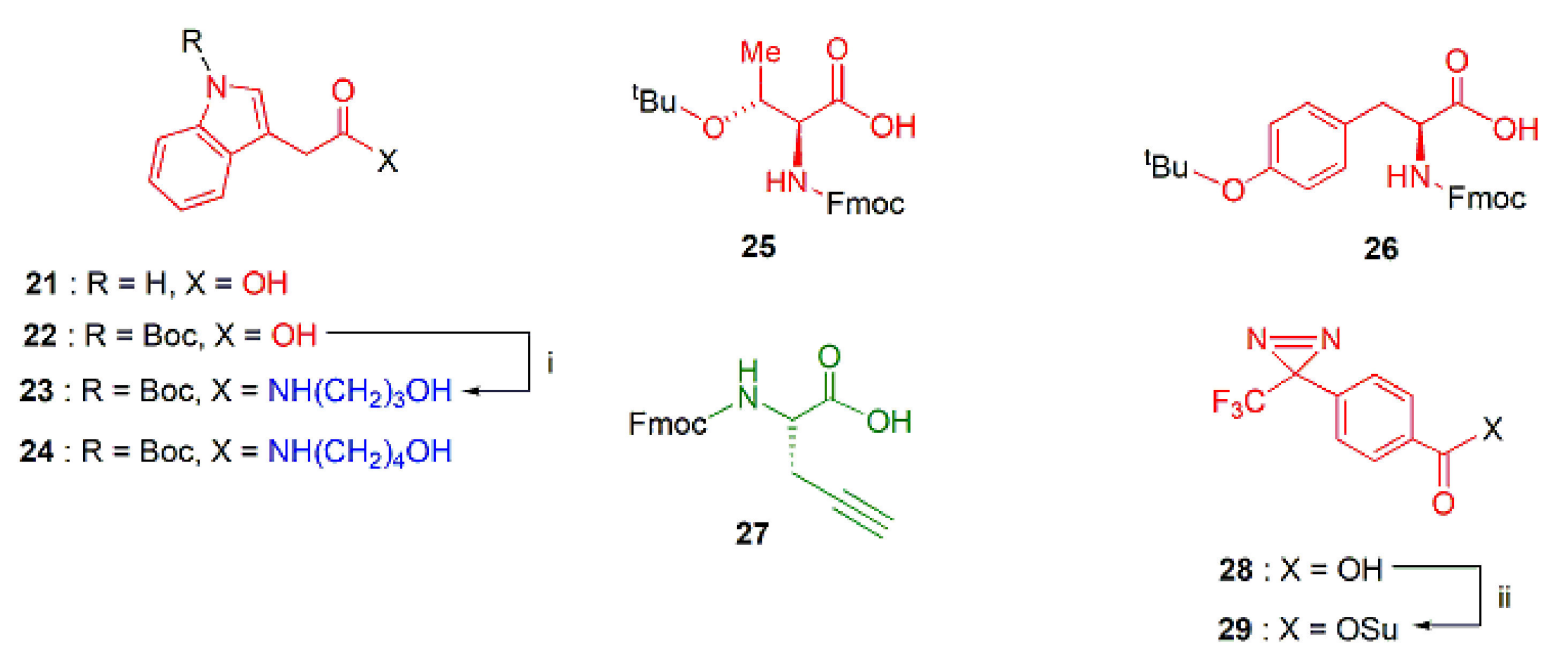
Structures of compounds suitable for the attachment of the lipophilic head groups to the PA chains. *Reagents and conditions*: (i) HOSu/DCC, DMF, 0 °C, 30 min then 25 °C, 2 h, followed by addition of H_2_N(CH_2_)_3_OH, 25 °C, 10 min, 80%; (ii) HOSu/DCC, THF, 0 °C, 30 min then 25 °C, 2 h, 87%.

The lipophilic head groups of the synthesized PAT analogs **1**–**10** were attached to the PA chains using either commercially available building blocks, such as compounds **21** and **25**–**28**, or suitably derivatized intermediates, such as **23** or the isolable succinimidyl ‘active’ ester **29** (Figure 4). Compound **23** was prepared from carboxylic acid **22** [13] in 80% yield in an analogous manner to that described for its homologous compound **24** [13]. On the other hand, ‘active’ ester **29**, a commercially available but very expensive compound (CAS 87736-89-8), was readily prepared for the needs of the present work in 91% yield through the condensation of acid **28** and *N*-hydroxysuccinimide (HOSu) in the presence of the coupling agent *N*,*N*’-dicyclohexylcarbodiimide (DCC). 

#### 2.1.3. Assembly of the Conjugates

##### Shorter PA Chain Analogs **1**–**3** of PATs **Agel 416** and **HO-416b**

**Scheme 1:**
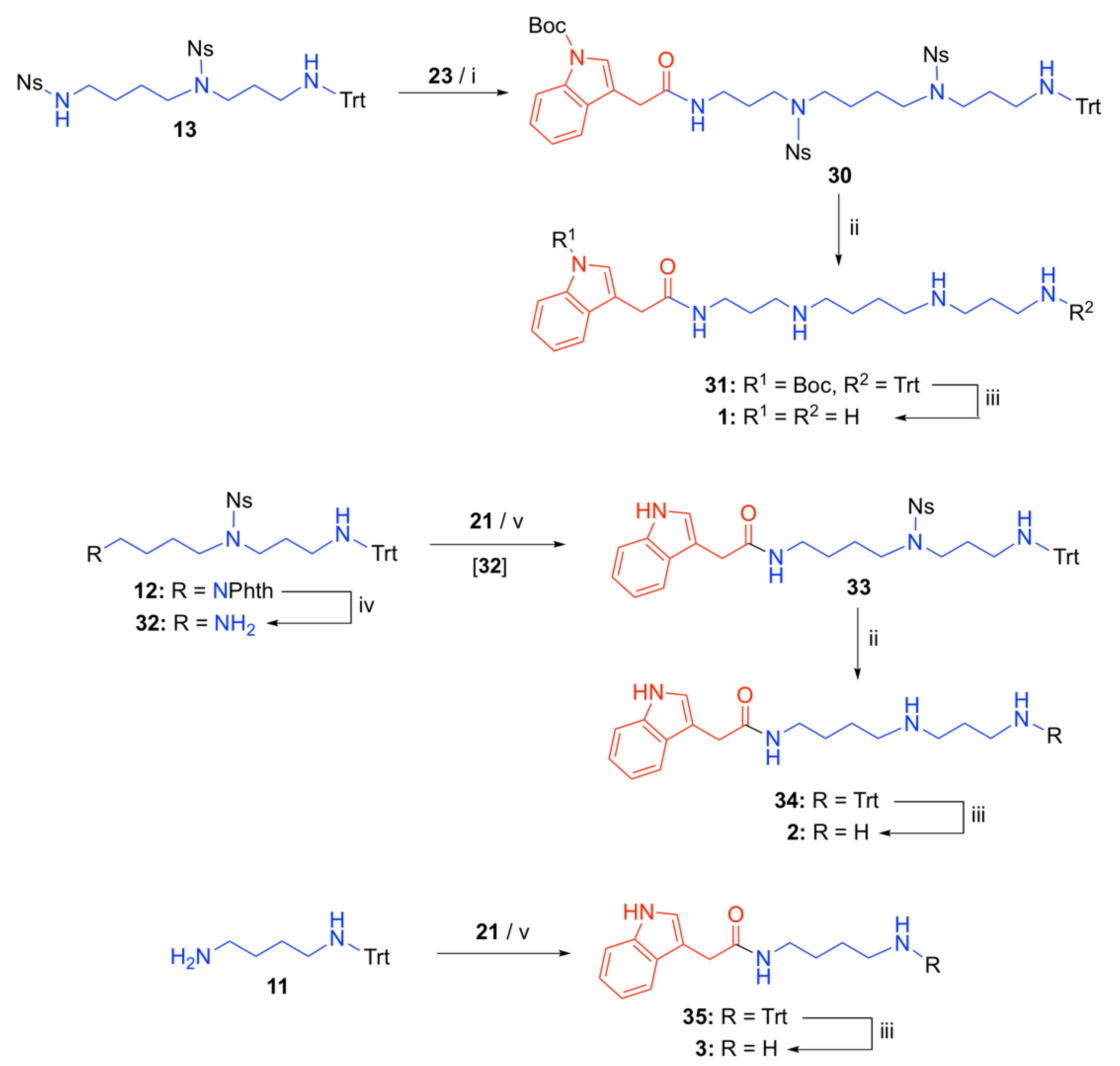
Outline of synthetic routes to shorter PA chain analogs **1**–**3** of the PATs **Agel 416** and **HO-416b**. *Reagents and conditions*: (i) Ph_3_P/^i^PrO_2_CN=NCO_2_^i^Pr, THF, 0 °C, 15 min then 25 °C, 2 h, 48%; (ii) PhSH/Na_2_CO_3_, DMF, 25 °C, 12 h, 46–61%; (iii) 10% TFA in CH_2_Cl_2_, PhSH, 25 °C, 1 h, 53–67%; (iv) H_2_NNH_2_^.^H_2_O, EtOH, 80 °C, 2 h, 87%; (v) (a) DCC/HOSu, DMF, 0 °C, 30 min then 25 °C, 2h, (b) Et_3_N, 25 °C, 2 h, 65–75%.

The synthesis of **Agel 416** analog **1** (Scheme 1), incorporating the Spm chain, initially involved the condensation of the dinosylated spermidine (Spd, a 3-4 PA) derivative **13** with alcohol **23** under Mitsunobu reaction conditions providing the fully protected Spm conjugate **30** in 48% yield. Then, selective denosylation of the secondary amino functions of **30** was performed using sodium thiophenolate providing the new intermediate **31** in 61% yield. Finally, both the *tert*-botuxycarbonyl (Boc) and the triphenylmethyl (trityl, Trt)-protecting groups were removed upon trifluoroacetic acid (TFA)-mediated acidolysis yielding **Agel 416** analogue **1**, isolated as its corresponding tetratrifluoroacetate salt, in 53% yield.

On the other hand, the synthesis of **HO-416b** analog **2**, incorporating the Spd chain, initially involved the removal of the phthalyl (Phth)-protecting group from the orthogonally protected Spd derivative **12** [13], through hydrazinolysis in refluxing ethanol, yielding the selectively deprotected Spd derivative **32** in 87% yield. The primary amino function of compound **32** was then acylated with (indol-3-yl)acetic acid (**21**), which had been previously activated with the coupling agent DCC in the presence of the coupling auxiliary HOSu. That way, the intermediate **33** was obtained in 65% yield. Treatment of the latter with sodium thiophenolate in DMF resulted in the selective deprotection of the secondary amino function yielding the new intermediate **34** in 46% yield. Finally, acidolysis of the Trt amino-protecting group with 10% TFA in DCM, in the presence of PhSH serving as carbocation scavenger, provided the PAT analog **2** in 55% yield.

The synthesis of **HO-416b** analog **3**, incorporating the putrescine (Put, a 4 PA) chain, initially involved the acylation of the monotritylated Put derivative **11** with acid **21**, which had been previously activated with DCC and HOSu, yielding the intermediate **35** in 75% yield. Then, acidolysis of the Trt amino-protecting group with 10% TFA in DCM, in the presence of PhSH, provided the PAT analog **3** in 67% yield.

##### PAT Analogs **4** and **9**–**10** Incorporating the Tsm Chain

**Scheme 2:**
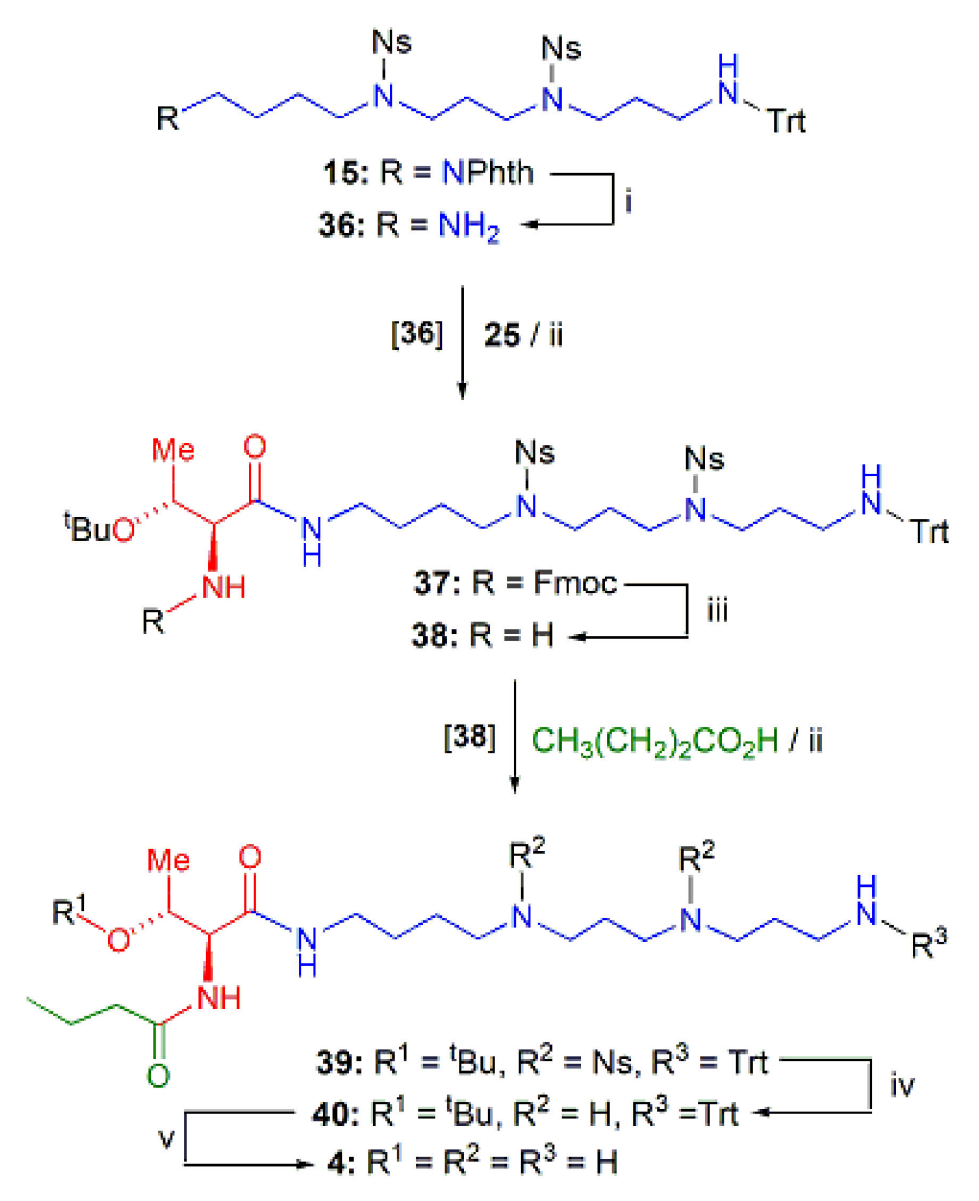
Outline of synthetic route to PAT analog **10**. *Reagents and conditions*: (i) H_2_NNH_2_^.^H_2_O, EtOH, 80 °C, 1 h, 71%; (ii) HBTU, ^i^Pr_2_NEt, DMF, 0 °C, 5 min then 25 °C, 30 min, 61–81%; (iii) 25% Et_2_NH in CH_2_Cl_2_, 25 °C, 1 h, 78%; (iv) PhSH/Na_2_CO_3_, DMF, 25 °C, 12 h, 51%; (v) 50% TFA in CH_2_Cl_2_, PhSH, 25 °C, 1 h, 89%.

The synthesis of the PhTX-433 analog **4**, in which the aromatic amino acid L-tyrosine (L-Tyr) has been replaced by the aliphatic amino acid L-threonine (L-Thr), is outlined in Scheme 2. 

It starts from the orthogonally protected Tsm derivative **15**, whose synthesis has been described above (Figure 3). Compound **15**, upon hydrazinolysis, yielded the selectively protected Tsm derivative **36** in 71% yield. This was then coupled with the commercially available Fmoc-L-Thr(^t^Bu)-OH (**25**) in the presence of the coupling agent 2-(1*H*-benzotriazol-1-yl)-1,1,3,3-tetramethyluronium hexafluorophosphate (HBTU) and ethyldiisopropylamine (DIPEA) in DMF yielding the conjugate **37** in 81% yield. Removal of the 9-fluorenylmethoxycarbonyl (Fmoc)-protecting group from the amino acid residue took place with 25% diethylamine in DCM yielding the partially protected conjugate **38**, which upon coupling with butanoic acid, also in the presence of the coupling agents HBTU and DIPEA, yielded bisamide **39** in 48% yield for the two last steps. Treatment of intermediate **39** with sodium thiophenolate in DMF resulted in the removal of the nosyl-protecting groups yielding the partially protected PAT analog **40** in 51% yield. Finally, both the *tert*-butyl (^t^Bu) and the Trt-protecting groups were removed on treatment with 50% TFA in DCM, in the presence of PhSH, yielding the PAT analog **4**, in the form of its corresponding tritrifluoroacetate salt, in 89% yield.

**Scheme 3:**
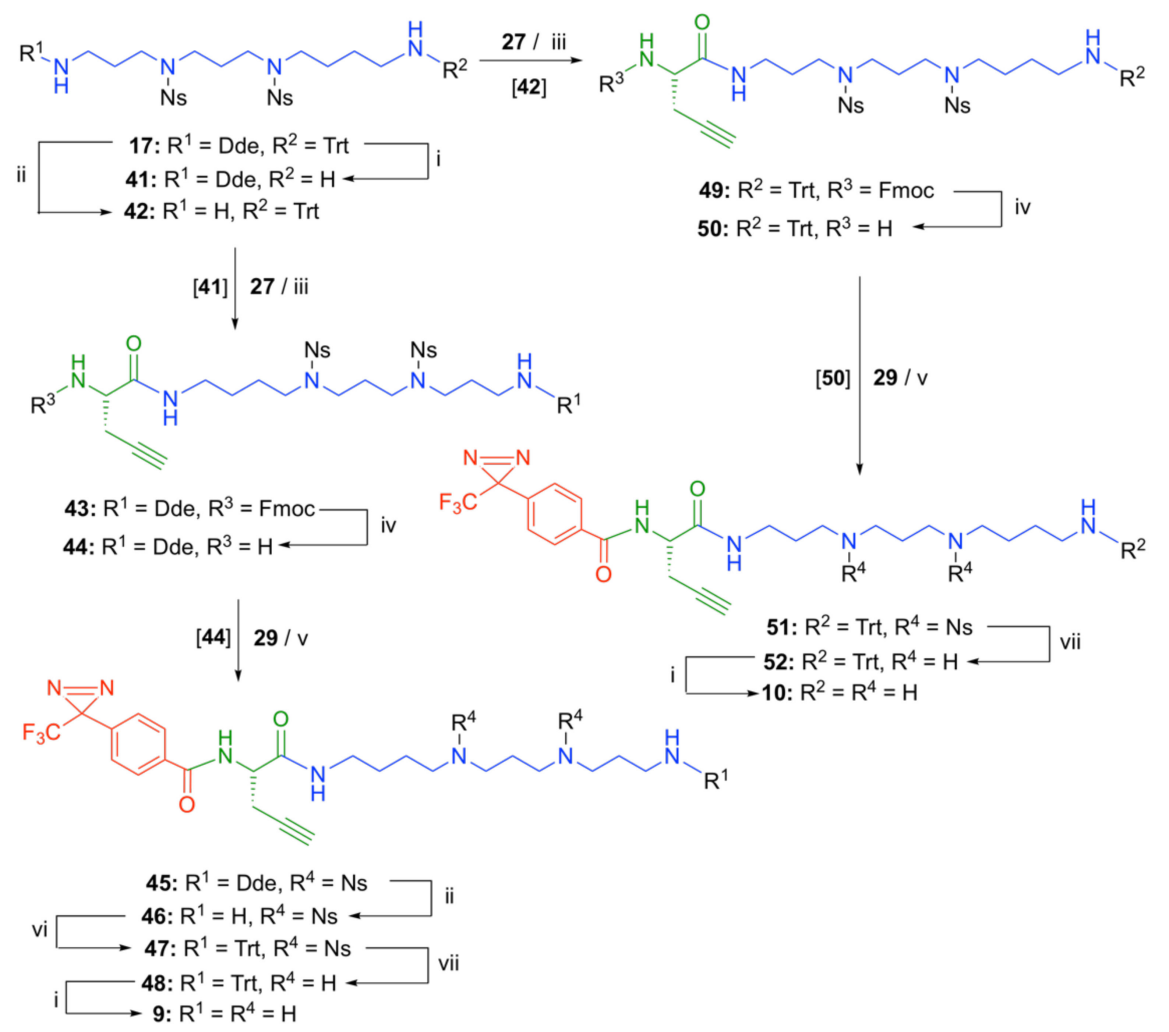
Outline of synthetic routes to PAT analogs **9** and **10**. *Reagents and conditions*: (i) 10% TFA in CH_2_Cl_2_, PhSH, 25 °C, 0.5–1 h, 65% (**9**) and 87% (**10**); (ii) 2% H_2_NNH_2_⋅H_2_O in DMF, 25 °C, 30–90 min, 79% (**42**) and 73% (**46**); (iii) PyBrOP, ^i^Pr_2_NEt, DMF, 0 °C, 5 min then 25 °C, 30 min, 70% (**43**) and 76% (**49**); (iv) 25% Et_2_NH in CH_2_Cl_2_, 25 °C, 1–3 h; (v) ^i^Pr_2_NEt, DMF, 25 °C, 12 h, 71% (**45**) and 74% (**51**); (vi) TrtCl, Et_3_N, CH_2_Cl_2_, 25 °C, 1 h; (vii) PhSH/Na_2_CO_3_, DMF, 25 °C, 3–5 h, 75% (**48**) and 92% (**52**).

On the other hand, the synthesis of the regioisomeric PAT analogs **9** and **10**, incorporating an amino acid and a lipophilic head group at N-12 and the N-1 positions, respectively, of the Tsm skeleton, is outlined in Scheme 3. Both syntheses have a common starting material, namely the orthogonally protected Tsm derivative **17** [13]. Treatment of compound **17** with 10% TFA in DCM resulted in the selective removal of the Trt-protecting group yielding the partially protected Tsm derivative **41**, whereas treatment of the same compound with 2% H_2_NNH_2_⋅H_2_O in DMF resulted in the removal of the Dde-protecting group providing the alternative partially protected Tsm derivative **42** in 79% yield. Tsm derivatives **41** and **42** are key intermediates in the assembly of the skeleton of PAT analogs **9** and **10**, respectively.

Thus, coupling of compounds **41** or **42** with the commercially available *N*-Fmoc-protected amino acid (*S*)-propargylglycine in the presence of the coupling agent bromotripyrrolidinophosphonium hexafluorophosphate (PyBrOP) and DIPEA in DMF produced the corresponding amides **43** and **49** in 70% and 76% yields, respectively, for the two steps (detritylation and coupling). Removal of the Fmoc-protecting group from these compounds with 20% Et_2_NH in DCM produced the corresponding partially protected conjugates **44** and **50**, respectively. Each one of these conjugates were then acylated by the succinimidyl ‘active’ ester **29** in the presence of DIPEA in DMF to give bisamides **45** and **51** in 71% and 74% yield for the last two steps, respectively. In compound **45**, we first proceeded with the replacement of the Dde-protecting group by the highly lipophilic Trt group, which facilitates aqueous work-up procedures and purifications with FCC chromatography [13], as follows. Treatment of bisamide **45** with 2% H_2_NNH_2_⋅H_2_O in DMF produced the intermediate **46** in 73%, which was then tritylated with TrtCl and Et_3_N in DCM to give the new intermediate **47**. Finally, the Ns-protecting groups were removed from the intermediates **47** and **51** upon treatment with sodium thiophenolate in DMF, yielding the N-tritylated intermediates **48** and **52** in 75% and 92% yield, respectively, for the two steps (tritylation and denosylation). From these compounds, the projected PAT analogs **9** and **10** were obtained as the corresponding tritrifluoroacetate salts in 65% and 87% yield, respectively, upon TFA-mediated acidolysis.

##### PAT Analogs **5** and **6** Incorporating the Spm Chain

**Scheme 4:**
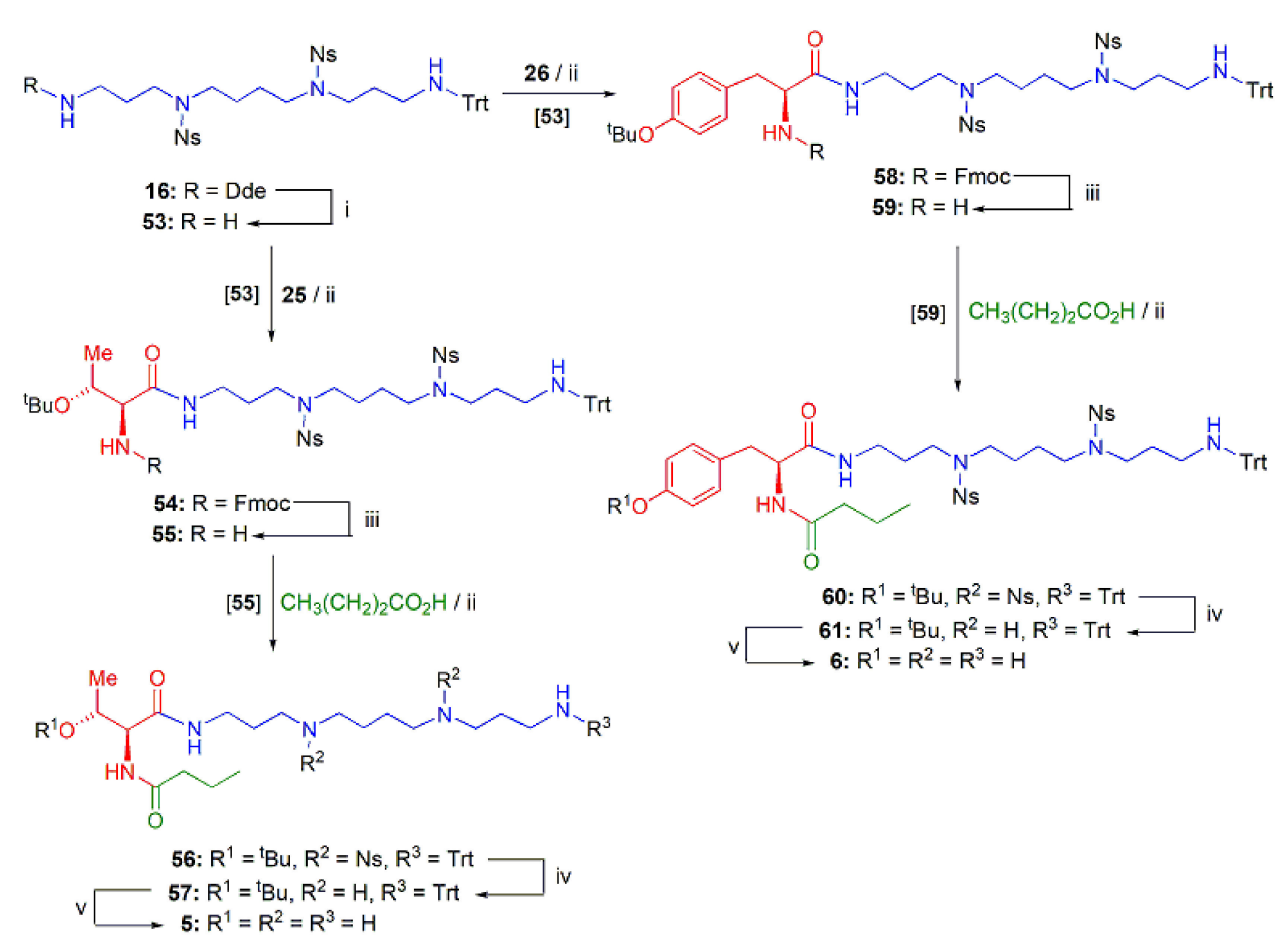
Outline of synthetic routes to PAT analogues **5** and **6**. *Reagents and conditions*: (i) 2% H_2_NNH_2_⋅H_2_O in DMF, 25 °C, 30 min, 91%; (ii) HBTU, ^i^Pr_2_NEt, DMF, 0 °C, 5 min then 25 °C, 30 min, 78% (**54**), 57% (**62**) and 64% (**56**); (iii) 25% Et_2_NH in CH_2_Cl_2_, 25 °C, 1 h, 90% (**55**); (iv) PhSH/Na_2_CO_3_, DMF, 25 °C, 12 h, 72% (**57**) and 65% (**61**); (v) 50% TFA in CH_2_Cl_2_, PhSH, 25 °C, 1 h, 87% (**5**) and 90% (**9**).

The synthesis of the PhTX-433 analog **5**, in which the Tsm chain has been replaced by the Spm chain and the amino acid L-Tyr in the lipophilic head group has been replaced by L-Thr, is outlined in Scheme 4. 

It started from the orthogonally protected Spm derivative **16** [13]. Compound **16**, upon the selective removal of the Dde-group with 2% H_2_NNH_2_⋅H_2_O in DMF, yielded the selectively protected Spm derivative **53** in 91% yield. This was then coupled with the commercially available Fmoc-L-Thr(^t^Bu)-OH (**25**) in the presence of the coupling agent HBTU and DIPEA, yielding the conjugate **54** in 78% yield. Removal of the Fmoc-protecting group from the amino acid residue took place with 25% diethylamine in DCM, yielding the partially protected conjugate **55** in 90% yield, which upon coupling with butanoic acid, also in the presence of HBTU and DIPEA, yielded the bisamide **56** in 57% yield. Treatment of intermediate **56** with sodium thiophenolate in DMF resulted in the removal of the nosyl-protecting groups yielding the partially protected intermediate **57**. Finally, the ^t^Bu- and the Trt-protecting groups were removed upon treatment with 50% TFA in DCM, in the presence of PhSH, yielding the PAT analog **5**, in the form of its corresponding tritrifluoroacetate salt, in 87% yield for the last two steps.

The synthesis of the PhTX-433 analog **6**, in which the Tsm chain has been replaced by the Spm chain, is also outlined in Scheme 4. It started from the partially protected Spm derivative **53**. Coupling of this compound with the commercially available Fmoc-L-Tyr(^t^Bu)-OH (**26**) in the presence of the coupling agent HBTU and DIPEA yielded the conjugate **58**. Removal of the Fmoc-protecting group from the amino acid residue took place with 25% diethylamine in DCM, yielding the partially protected conjugate **59**, which upon coupling with butanoic acid, also in the presence of HBTU and DIPEA, provided the bisamide **60** in 64% yield for the last three steps. Treatment of intermediate **60** with sodium thiophenolate in DMF resulted in the removal of the nosyl-protecting groups yielding the intermediate **61** in 65% yield. Finally, the ^t^Bu- and the Trt-protecting groups were removed uon treatment with 50% TFA in DCM, also in the presence of PhSH, yielding the PhTX-433 analog **6**, in the form of its corresponding tritrifluoroacetate salt, in 90% yield.

It should be noted that an extended array of PhTX-433 analogs, including PhTX-343, has been previously synthesized by several research groups, both in solution and solid phases, in order to be studied as antagonists of transmitter receptors [11,14,15,16,17,18,19,20,21].

##### Analogs **7** and **8** of PhTX-433 with Longer PA Chains

**Scheme 5:**
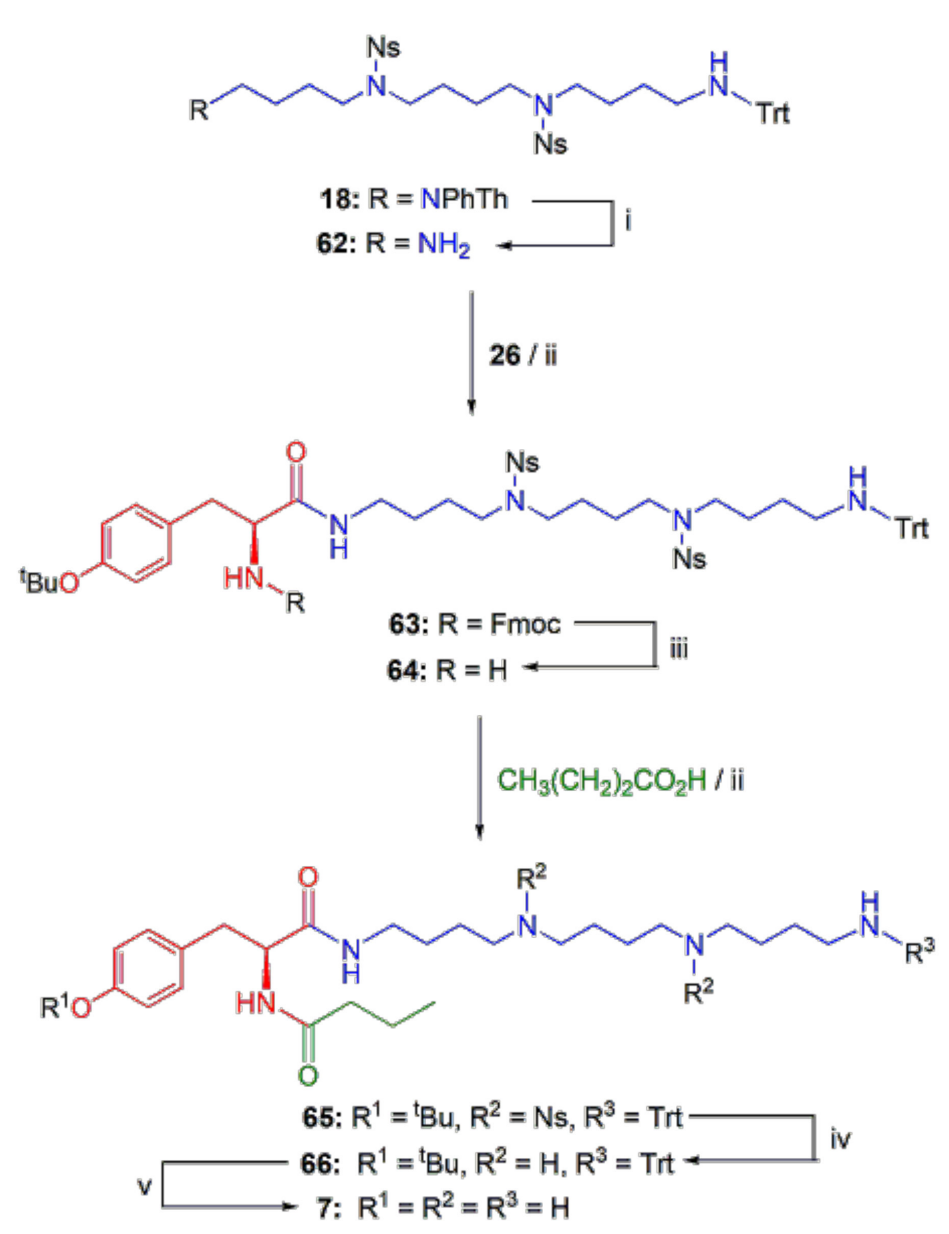
Outline of synthetic route to PhTX-433 analog **7**. *Reagents and conditions*: (i) H_2_NNH_2_^.^H_2_O, EtOH, 80 °C, 2 h, 41%; (ii) HBTU, ^i^Pr_2_NEt, DMF, 0 °C, 5 min then 25 °C, 30 min, 73% (**69**); (iii) 25% Et_2_NH in CH_2_Cl_2_, 25 °C, 1 h; (iv) PhSH/Na_2_CO_3_, DMF, 25 °C, 12 h, 79%; (v) 50% TFA in CH_2_Cl_2_, PhSH, 25 °C, 1 h, 85%.

The synthesis of the PhTX-433 analog **7**, in which the Tsm chain has been replaced by the longer homospermine (Hsm, a 4-4-4 PA) chain, is outlined in Scheme 5. It started with the hydrazinolysis in refluxing ethanol of the orthogonally protected Hsm derivative **18 [13]** yielding the partially protected Hsm derivative **62** in 41% yield. This was then coupled with the commercially available Fmoc-L-Tyr(^t^Bu)-OH (**26**) in the presence of the coupling agent HBTU and DIPEA, yielding the conjugate **63** in 73% yield.

Removal of the Fmoc-protecting group from the amino acid residue took place with 25% diethylamine in DCM yielding the partially protected conjugate **64**, which upon coupling with butanoic acid, also in the presence of HBTU and DIPEA, yielded the bisamide **65**. Treatment of intermediate **65** with sodium thiophenolate in DMF resulted in the removal of the nosyl-protecting groups yielding the partially protected PhTX-433 analog **66** in 79% overall yield for the last three steps. Finally, the ^t^Bu- and the Trt-protecting groups were removed upon treatment with 50% TFA in DCM, also in the presence of PhSH, yielding the PhTX-433 analog **7**, in the form of its corresponding tritrifluoroacetate salt, in 85% yield.

**Scheme 6:**
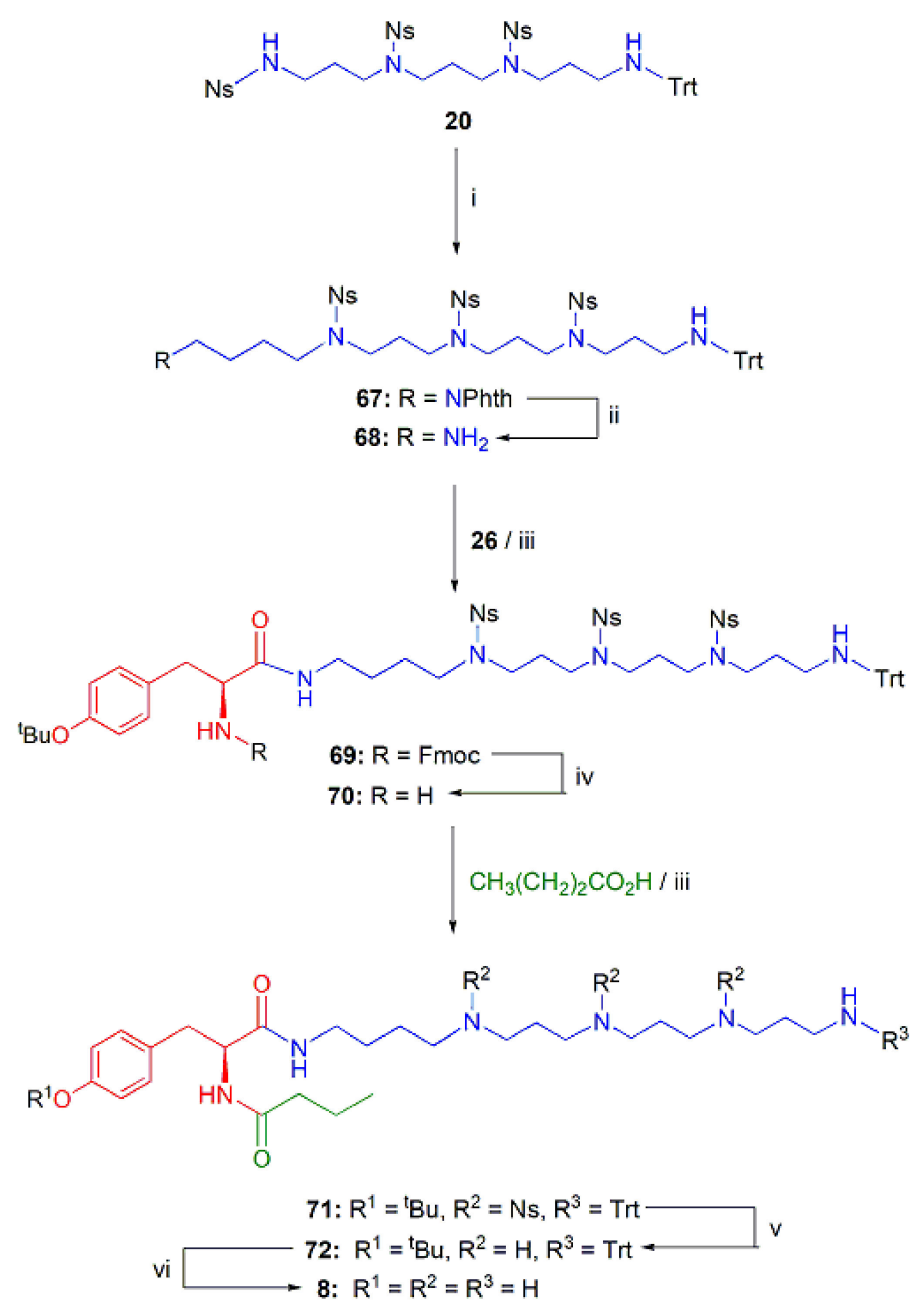
Outline of synthetic route to PhTX-433 analog **8**. *Reagents and conditions*: (i) PhthN(CH_2_)_4_Br, K_2_CO_3_, DMF, 60 °C, 2.5 h; (ii) H_2_NNH_2_^.^H_2_O, EtOH, 80 °C, 1 h, 52%; (iii) HBTU, ^i^Pr_2_NEt, DMF, 0 °C, 5 min then 25 °C, 30 min, 60% (**69**); (iv) 25% Et_2_NH in CH_2_Cl_2_, 25 °C, 1–2 h; (v) PhSH/Na_2_CO_3_, DMF, 25 °C, 12 h, 74%; (vi) 50% TFA in CH_2_Cl_2_, PhSH, 25 °C, 1 h, 83%.

On the other hand, the synthesis of the PhTX-433 analog **8**, in which the Tsm chain has been replaced by the longer 3-3-3-4 PA chain, is outlined in Scheme 6. It started from the norspermine (Nsm, a 3-3-3 PA) derivative **20**, whose preparation from the orthogonally protected Nsm derivative **19** [13] has been described above (Figure 3). It involved the alkylation of the primary nosylamide group of **20** with PhthN(CH_2_)_4_Br in DMF in the presence of K_2_CO_3_, yielding the orthogonally protected 3-3-3-4 PA derivative **67**. Hydrazinolysis of phthalimide **67 [13]** in refluxing ethanol yielded the partially protected 3-3-3-4 PA derivative **68** in 52% yield for the two steps. This was then coupled with the commercially available Fmoc-L-Tyr(^t^Bu)-OH (**26**) in the presence of the coupling agent HBTU and DIPEA, yielding the conjugate **69** in 60% yield. 

Removal of the Fmoc-protecting group from the amino acid residue took place with 25% Et_2_NH in DCM, yielding the partially protected conjugate **70**, which upon coupling with butanoic acid in the presence of HBTU and DIPEA, yielded the bisamide **71**. Treatment of intermediate **71** with sodium thiophenolate in DMF resulted in the removal of the nosyl-protecting groups yielding the partially protected PhTX-433 analog **72** in 74% yield for the last three steps. Finally, the ^t^Bu- and the Trt-protecting groups were removed upon treatment with 50% TFA in DCM, in the presence of PhSH, yielding the PhTX-433 analog **8**, in the form of its corresponding tetratrifluoroacetate salt, in 83% yield.

### 2.2. Biological Evaluation

#### 2.2.1. Antiproliferative Activity on Breast Cancer Cells

The antiproliferative activity of the synthesized compounds was evaluated using MCF-7 and ΜDA-MB-231 breast carcinoma cells. MCF-7 cells are characterized by epithelial morphology, low metastatic potential, and low aggressiveness, and are estrogen receptor alpha (ERα)-positive, whereas MDA-MB-231cells are characterized by mesenchymal morphology, high metastatic potential, and high aggressiveness, and are ERβ-positive. The cells were treated for 24 h with compounds **1**–**10** in a range of concentrations, from 0 to 200 or 400 μM. In addition, the cells were treated with acid **21** alone and an equimolar mixture of acid **21** and Spd (the PA substructure of conjugate **2**), to determine the effect of conjugation on the antiproliferative activity. The estimated IC_50_ values for these compounds are provided in Table 1 (see also Supplementary Material, Table S1). For the sake of comparison, the ΙC_50_ values of PATs **HO-416b** and **Agel 416**, determined previously [6], are included in Table 1. Calculated LogD values at pH 7.4, the physiological pH of blood serum, using ChemAxon’s Chemicalize platform are also included in Table 1.

#### 2.2.2. Cytotoxicity

**Figure 5:**
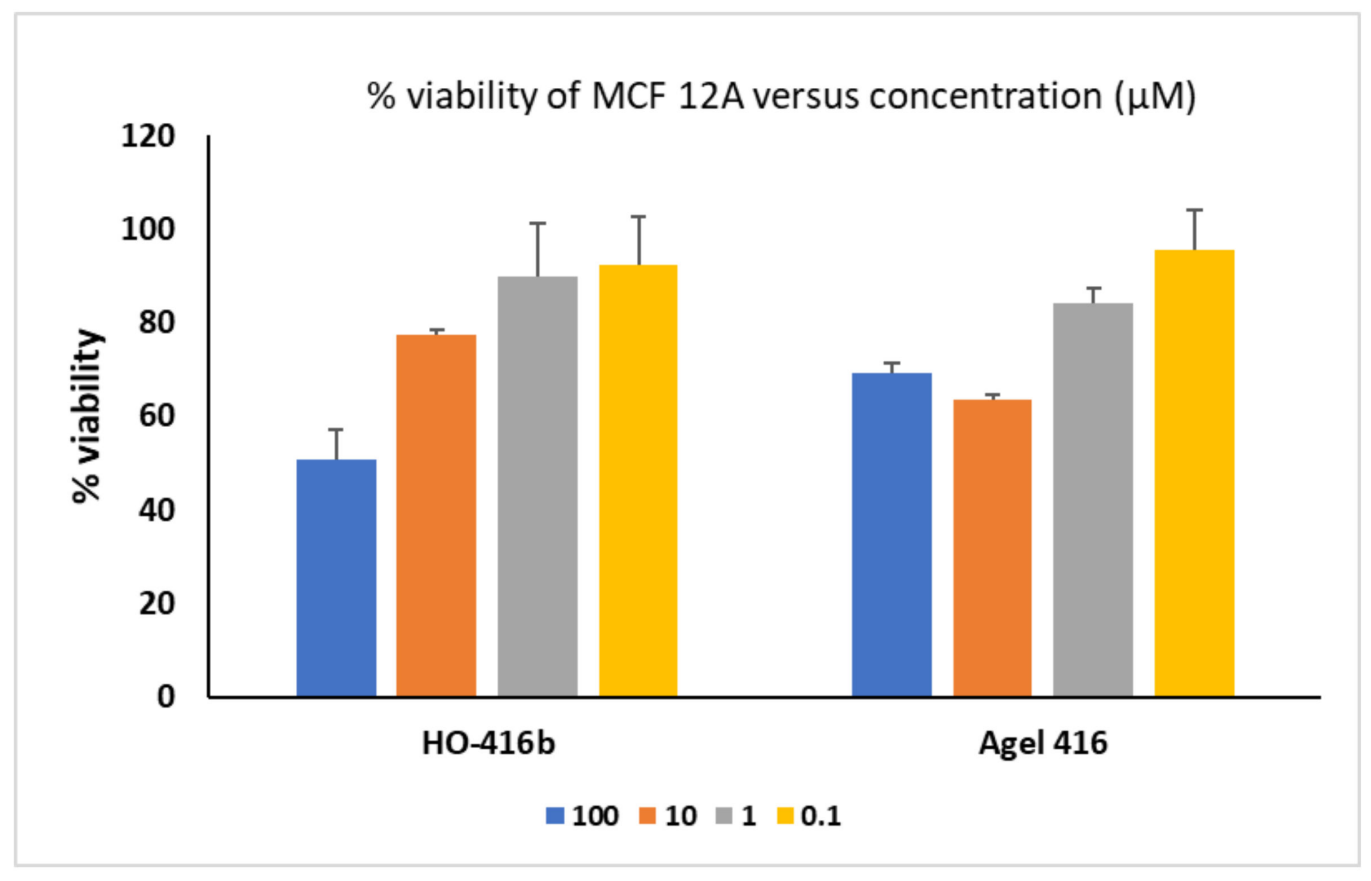
Viability of MCF-12A cells following treatments with PATs **HO-416b** and **Agel 416**.

The cytotoxity of the two most potent antiproliferative agents in this series of compounds, namely PATs **Agel 416** and **HO-416b**, was studied using the normal epithelial mammary cells MCF-12A, and the results are presented in Figure 5 (see also Supplementary Material, Figure S1). The IC_50_ values of the tested compounds are summarized in Table 2 (see also Supplementary Material, Table S2). 

#### 2.2.3. Structure–Activity Relationships

##### Antiproliferative Activity on the MCF-7 Cells

The synthesized PATs and analogs can be divided into three subgroups according to the lipophilic head group they are incorporating. The first subgroup consisted of spider toxins **Agel 416** and **HO-416b** and analogs thereof and is characterized by the presence of the indole nucleus as the head-group attached to a PA skeleton of variable length and number of nitrogen atoms. The two most potent compounds in this cohort are the PATs **HO-416b** and **Agel 416**, with IC_50_ values of 0.09 and 0.55 μM, incorporating either the 4-3-3-3 or the 3-3-4-3 pentaamine skeleton with the former being *ca* 6 times more cytotoxic. From the other three PAT analogs, incorporating the common PAs Put (a 4 diamine), Spd (a 4-3 triamine), and Spm (a 3-4-3 tetraamine), the most potent compound was analog **2**, incorporating Spd with an IC_50_ value of 3.15 μM. On the other hand, the analogues **1**, incorporating the PA Spm, and **3**, incorporating the PA Put, exhibited very low activity (IC_50_ > 200 μM and 272 μM, respectively). Analog **2**, sharing the 4-3 PA fragment of the most toxic PAT **HO-416b**, is 35 times less active than **HO-416b**. Therefore, we suggest that the pentaamine skeleton secures the highest possible activity followed by the 4-3 triamine skeleton provided by the PA Spd. Notably, (indol-3-yl)acetic acid (**21**) also had a very low antiproliferative activity (IC_50_ = 365.9 μM), which was improved (IC_50_ = 145.6 μM) when it was used in combination with an equimolar quantity of free Spd. It is apparent, however, that the conjugation of the two molecules created the PAT analog **2**, which was significantly more active (*ca* 42 times) than their mere combination.

The second subgroup involved analogs of PAT PhTX-433, isolated from wasps, with changes in the lipophilic head-group (analog **4**) or the PA chain (analogs **6**–**8**) or both (analog **5**). However, all these PhTX-433 analogs had very low antiproliferative activity (IC_50_ > 400 μM), even the analog **8**, which incorporated the 4-3-3-3 pentaamine skeleton seen in the very active PAT **HO-416b**. These results verify the importance of the structure of the head group on the antiproliferative activity of these compounds noticed earlier [5]. In that study, a 4-hydroxyphenyl ring in the lipophilic head group seems to be more important than an indole ring for the antiproliferative activity. However, concerning our compounds it seems that it is the indole ring of the head group that provides the highest activity (e.g., PAT **HO-416b**) and not the 4-hydroxyphenyl ring (e.g., PhTX-433 analog **8**). Interestingly, by comparing the dose-dependent response (DDR) diagrams of these compounds it is evident that the 4-4-4 PA skeleton secures higher activity than the 4-3-3-3 PA skeleton and that replacement of the aromatic Tyr residue by the aliphatic Thr residue leads to higher activity when combined with the 4-3-3 PA (Tsm) skeleton.

The third subgroup includes the compounds **9** and **10** in which the lipophilic head group is attached to either the N1 or the N12 amino function of the natural PA Tsm. The lipophilic head group consisted of a phenyl ring, substituted at position 4 by the photoactivatable 3-trifluoromethyldiazirin-3-yl ring, connected to the Tsm tetraamine chain through the clickable amino acid *S*-propargylglycine. Interestingly, both compounds showed strong antiproliferative activity with IC_50_ values of 2.63 and 2.81 μM, respectively, which makes them interesting tools for identifying potential macromolecular targets responsible for their activity. It seems that the position of the attachment of the lipophilic group on the PA chain is not especially important as both 3-3-4 and 4-3-3 PA arrangements in the conjugates lead to comparable activity, with the latter being slightly more potent than the former. 

It should be noted that the observed strength of the antiproliferative activity of PATs **Agel 416** and **HO-416b** and the synthesized analogs **1**–**10** on the MCF-7 cells could not be correlated to their lipophilicity, a key property in drug action and safety [22]. For the present set of compounds, LogD is the preferred descriptor for lipophilicity due to the ionizable nature of their PA chains [22]. All compounds presented low lipophilicity ranging from -1.49 (analog **3**) to −8.16 (analog **5**). This is, of course, due to the fact that PAs at physiological pH exist in their corresponding polycationic form [2,23], and therefore it seems that the hydrophilicity of the PA chains surpasses the lipophilicity of the head groups, especially with the increase in the number of N atoms in the PA chain. From the five most active compounds, namely **HO-416b**, **Agel 416**, **9**, **10**, and **2**, the first two are characterized by very low LogD values (−6.25 and −7.54) whereas the LogD values from the remaining three analogs range from −2.84 to −5.34. On the other hand, the least active analogs with tetraamine or pentaamine chains (analogs **1**, **4**, **5**, and **6**–**8**) are also characterized by very low LogD values ranging from −5.97 to −8.16 whereas analog **3**, with the shortest PA chain (Put) and the highest lipophilicity (LogD −1.49), presented very low antiproliferative activity (272 μM). However, the low lipophilicities of the PAs, PATs, and analogs do not hinder these molecules from passing the cellular membranes with the aid of polyamine uptake systems [23] and interacting effectively with their targets, such as nucleic acids, proteins, ion channels, and phospholipids [2]. It should be noted that the distance between the amino functionalities within a PA chain plays an important role in their recognition by the PA uptake systems [2]. In an attempt to correlate the lipophilicity of the head groups of the PATs and analogs to the observed antiproliferative activity, we calculated the LogP values, using the Chemicalize platform, of the neutral carboxylic acids *N*-(4-(3-(trifluoromethyl)-3*H*-diazirin-3-yl)benzoyl)-l-propargylglycine (LogP = 2.545), *N*-butanoyl-L-Tyr (LogP = 1.738), (indol-3-yl)acetic acid (Log P = 1.71), and *N*-butanoyl-L-Thr (LogP −0.245). Although the lipophilicity of the head groups would then be correlated to the strong antiproliferative activity of **Agel 416**, **HO-416b**, and analogs **2**, **9**, and **10**, compared for example to analogs **4** and **5**, the correlation fails with analogs **6**–**8** whose lipophilic head group is of comparable lipophilicity to that of **Agel 416**, **HO-416b**, and analog **2**. The same applies for analogs **1** and **3** bearing the (indol-3-yl)acetate head group. 

##### Antiproliferative Activity on the MDA-MB-231 Cells

The two most potent compounds in the first subgroup are, again, the PATs **HO-416b** and **Agel 416**, with comparable IC_50_ values of 3.98 and 3.31 μM, incorporating either the 4-3-3-3 or the 3-3-4-3 pentaamine skeleton. Compared to **Agel 416**, which is only 6 times less cytotoxic to the MDA-MB-231 cells than the MCF-7 cells, **HO-416b** is *ca* 44 times less cytotoxic. From the other three PAT analogs, incorporating the PAs Put, Spd, and Spm, the most potent compound was, again, analog **2** incorporating the PA Spd with an IC_50_ value of 12.6 μM. The other two analogs with the PA Spm or Put skeleton were either of too low (analog **1**, IC_50_ > 200 μM) or low activity (analog **3**, IC_50_ = 170.2 μM). Analog **2** shares the 4-3 PA fragment of the toxic PAT **HO-416b** and is 4 times less active than **HO-416b**. Similarly, for the MDA-MB-231 cells, the pentaamine skeleton secures the highest possible activity followed by the 4-3 triamine skeleton provided by the PA Spd. It should be noted that the (indol-3-yl)acetic acid (**21**) alone had low antiproliferative activity (IC_50_ = 262.2 μM), which, in contrast to the MCF-7 cells, was not improved (IC_50_ = 282.5 μM) when it was used in combination with an equimolar quantity of free Spd. It is apparent here, too, that conjugation of the two molecules creating the PAT analog **2** had a significant effect on the antiproliferative activity (*ca* 22 times more active) than their mere combination.

All analogs of PAT PhTX-433, consisting the second subgroup, had very low antiproliferative activity (IC_50_ > 400 μM), even analog **8**, which incorporates the 4-3-3-3 pentaamine skeleton seen in the very active PAT **HO-416b**. These results again verify the importance of the structure of the head group on the antiproliferative activity of these compounds noticed earlier [5] and that for the present cohort of compounds, the indole ring of the head group provides the highest activity (e.g., PATs **HO-416b** and **Agel 416**) and not the 4-hydroxyphenyl ring (e.g., PhTX-433 analog **8**). Interestingly, by comparing the DDR diagrams of these compounds, it is evident that the 4-4-4 PA skeleton secures higher activity than the 4-3-3-3 PA skeleton and that replacement of the aromatic Tyr residue by the aliphatic Thr residue leads to higher activity when combined with the 4-3-3 PA skeleton.

Finally, both compounds **9** and **10** of the third subgroup showed low antiproliferative activity with IC_50_ > 200 μM and are the only compounds in the present series of compounds that show specificity for the MCF-7 cells. All other compounds showed either comparable activity for the two types of cells or a lack of significant activity for both types of cells. Furthermore, upon comparing the DDR diagrams of these two compounds it is evident that compound **10**, in which the lipophilic head group is attached to the N1 position of the Tsm skeleton (a 3-3-4 PA arrangement), is slightly more active than compound **9**, in which the lipophilic head-group is attached to position N12 of the Tsm skeleton (a 4-3-3 PA arrangement). 

As it is the case with the MCF-7 cells, the observed strength of antiproliferative activity of PATs **Agel 416** and **HO-416b** and the synthesized analogs **1**–**10** on the MDA-MB-231 cells could not be correlated to their lipophilicity. From the three most active compounds, namely **HO-416b**, **Agel 416**, and **2**, the first two are characterized by very low LogD values (−6.25 and −7.54) whereas the LogD value for analog **2** was -2.84. On the other hand, the least active analogs with tetraamine or pentaamine chains (analogs **1** and **4**–**10**) are also characterized by low or very low LogD values ranging from -3.87 to -8.16, whereas analog **3**, with the shortest PA chain (Put) and the highest lipophilicity (LogD −1.49), presented very low antiproliferative activity (107 μM).

##### Cytotoxicity on MCF-12A Noncancerous Cells

From the IC_50_ values given in Table 2, it is apparent that the two most potent antiproliferative compounds, namely PATs **Agel 416** and **HO-416b**, are cytotoxic in normal mammary MCF-12A cells only in high concentrations and that **HO-416b** is *ca* 1.9 times more cytotoxic than **Agel 416**. Taking into consideration these values and the IC_50_ values for the cytotoxicity of the compounds for the cancerous MCF-7 and MDA-MB-231 cells (Table 1), it is estimated that the selectivity index (SI) for PAT **Agel 416** is *ca* 335 for the MCF-7 cells and *ca* 56 for the MDA-MB-231 cells. The corresponding values for PAT **HO-416b** are *ca* 1080 and 24. 

## 3. Materials and Methods

### 3.1. General Information 

Melting points were determined with an Electrothermal apparatus and are uncorrected. ^1^H-NMR and ^13^C-NMR spectra were obtained at 600 and 150 MHz, respectively, on the Bruker AvanceIII HD spectrometer. Chemical shifts (δ) are reported for CDCl_3_ solutions in parts per million (ppm) downfield from tetramethylsilane (TMS), used as an internal standard, or for (CD_3_)_2_SO solutions. Electron-spray ionization (ESI) mass spectra were recorded at 30 eV, on a Waters Micromass ZQ spectrometer using HPLC grade MeOH or MeCN as a solvent, or MeCN/H_2_O as a solvent system. Elemental analyses for solid compounds were determined on a Carlo Erba EA 1108 CHNS elemental analyzer.

Analytical reversed-phase high-performance liquid chromatography (RP-HPLC) for compounds **1**–**3** was performed on an Agilent Technologies, 1260 Affinity, Quaternary Pump VL system equipped with a photodiode array detector. The purity of the final conjugates was determined using UV detection (λ = 254 nm). The chromatographic method employed was the following: Column, LiChrosorb RP18 (25 cm × 4.6 mm, 5 μm); mobile phase I, 0.08% TFA in water; mobile phase II, acetonitrile with 0.08% TFA; flow rate, 1.0 mL/min; elution profile, gradient elution from 5 to 50% II over 30 min. Analytical RP-HPLC for compounds **6**–**10** and the tetrabenzoylated derivatives of compounds **4** and **5** was performed on an Agilent Technologies 1100 system. The purity of the conjugates was determined using UV detection (λ = 220 nm). The chromatographic method employed was the following: Column, Luna C18 (100 × 4.6 mm, 3 μm); temperature, 40 °C; flow rate, 1.25 mL/min; elution time, 7 min; elution profile, gradient elution as shown in Table 3.

Compounds **4** and **5** were converted, prior to HPLC determination, to their corresponding tetrabenzoylated derivatives using a slight modification of the method described for the RP-HPLC determination of PAs [24]. Thus, to a solution of 5 μmole of conjugate **4** or **5** in 0.1 mL H_2_O, 1 mL of 2N aqueous NaOH and 5 μL of benzoyl chloride were added, and the resulting solution was shaken for 3 min with the aid of a vortex mixer and then allowed to stand for 20 min at ambient temperature. An additional 5 μL portion of benzoyl chloride was added, and shaking was repeated for 3 min. Following 20 min of standing, the solution was diluted with 2 mL brine and extracted twice with 2 mL diethyl ether. The organic layers were combined, dried (anhydrous Na_2_SO_4_), and evaporated to dryness under reduced pressure. The residue was diluted in 0.5 mL H_2_O and aliquots examined with RP-HPLC. The retention times and the ESI-MS spectra for these derivatives were as follows: Tetrabenzoylated **4** (C_46_H_55_N_5_O_7_, exact mass: 789.41): RP-HPLC: *t*_R_ = 4.128 min; MS (ESI, 30 eV): *m/z* 812.72 [M+Na]^+^, 790.76 [M+H]^+^. Tetrabenzoylated **5** (C_46_H_55_N_5_O_7_, exact mass: 789.41): RP-HPLC: *t*_R_ = 4.160 min; MS (ESI, 30 eV): *m/z* 812.79 [M+Na]^+^, 790.76 [M+H]^+^. All synthesized PAT analogs (compounds **1**–**10**) subjected to biological evaluation were >95% pure by RP-HPLC.

Flash column chromatography (FCC) was performed on Acros Organics silica gel 0.035–0.070 mm, 60 Å, and TLC on Merck silica gel 60 F_254_ films (0.2 mm) precoated on aluminum foil. Spots were visualized with UV light at 254 nm and by spraying with a ninhydrine solution (0.3 g ninhydrin, 3 mL gl. acetic acid, 97 mL 1-butanol). The solvent systems used for the development of TLC or FCC are the following: (A) CHCl_3_/MeOH (95:5), (B) CHCl_3_/MeOH (9:1), (C) CHCl_3_/MeOH (8:2), (D) CHCl_3_/MeOH/conc. NH_3_ (99:1:0.1), (E) CHCl_3_/MeOH/conc. NH_3_ (95:5:0.5), (F) CHCl_3_/MeOH/conc. NH_3_ (9:1:0.1), (G) CHCl_3_/MeOH/conc. NH_3_ (8:2:0.2), (H) CHCl_3_/MeOH/conc.NH_3_ (7:3:0.3), (I) CHCl_3_/MeOH/conc. NH_3_ (6:4:0.4), (J) PhMe/EtOAc (95:5), (K) PhMe/EtOAc (9:1), (L) PhMe/EtOAc (8:2), (M) PhMe/EtOAc (7:3), (N) PhMe/EtOAc (6:4), (O) PhMe/EtOAc (1:1), (P) EtOAc/PhMe (8:2), (Q) EtOAc/PhMe (7:3), (R) EtOAc (100%).

All solvents were dried and/or purified according to standard procedures prior to use. Solvents were routinely removed at *ca* 40 °C under reduced pressure on Büchi Rotavapor RE 111 apparatus. Air-sensitive reagents were handled under inert atmosphere (Ar). All reagents employed in the present work were purchased from either Sigma-Aldrich or Alfa Aesar or Merck or Acros Organics and were used without further purification. DIAD was used in the context of this work as a commercially available safer alternative to DEAD. The photo-affinity labeling (PAL) reagent 4-(3-trifluoromethy-3*H*-diaziridin-3-l)benzoic acid (**28**) was purchased from TCI and the amino acid derivatives Fmoc-L-Thr(^t^Bu)-OH (**25**), Fmoc-L-Tyr(^t^Bu)-OH (**26**), and Fmoc-propargylglycine (**27**) were purchased from Bachem or Iris Biotech. Succinimidyl 4-(3-(trifluoromethyl)-3*H*-diazirin-3-yl)benzoate (**29**) (CAS 87736-89-8) is a commercially available, very expensive compound, which was prepared as described in the Supplementary Materials for the needs of the present work. The intermediates *N*^4^,*N*^8^-dinosyl-N^1^-tritylspermidine (**13**), *N*^4^-nosyl-*N*^1^-tritylnorspermidine, *N*^4^,*N*^7^-dinosyl-*N*^1^-tritylnorspermidine, *N*^4^-nosyl-*N*^1^-tritylspermidine (**32**), and *N*^4^,*N*^9^-dinosyl-*N*^1^-tritylspermine (**53**), were synthesized, for the needs of the present work, according to procedures described in ref. [13]. Experimental details for the synthesis and characterization of intermediates and final products (PAT analogs **1**–**10**) of the present work are provided in the Supplementary Materials. 

### 3.2. Biological Evaluation

#### 3.2.1. Cell Cultures and Conditions

##### Breast Cancer Cells

MCF-7 and MDA-MB-231 breast cancer cell lines were purchased from the American Type Culture Collection (ATCC). All cells were routinely cultured in a humidified 95% air/5% CO_2_ incubator at 37 °C in complete medium Dulbecco’s Modified Eagle’s Medium (DMEM) supplemented with 10% (*v*/*v*) fetal bovine serum (FBS) 1.0 mM sodium pyruvate, 2 mM l-glutamine, and 100 IU/mL penicillin. The synthesized compounds were dissolved in ddH_2_O (or DMSO for compound **27** and the equimolar mixture of compounds **27** and Spd) at a final concentration of 10 mM and further dilutions were conducted with culture medium.

**Determination of IC50 values**

MCF-7 and ΜDA-MB-231 breast cancer cells were seeded in 12-well plates and were allowed to grow up to 60–70% confluence. After renewing the culture medium, cancer cells were treated for 24 h in serum-containing culture medium with compounds **1**–**10** in a range of concentrations, from 0 to 200 or 400 μM. In addition, cells were treated with acid **21** alone and an equimolar mixture of acid **21** and Spd in order to determine the effect of conjugation on the antiproliferative activity. Then, the surviving breast cancer cells were measured, either manually (for compounds **Agel 416**, **HO-416b**, **1**-**3**, **6**-**7**) or by measuring the absorbance following an MTT assay (for compounds **4**-**5**, **8**-**10**, **21** and **21**+Spd), as described below in detail. The percentage of live cells was plotted against the log values of compound concentrations, and a non-linear fit plot was used to estimate the IC_50_ value for each compound.

**Cytotoxicity Evaluation in Normal Mammary Cells**

Normal epithelial mammary cells MCF-12A were obtained from ATCC. The cells were cultured in DMEM-F12 containing 1% Penicillin/Streptomycin solution, 7% equine serum, 30 ng/mL epidermal growth factor, 0.5 μg/mL hydrocortisone, 0.1 μg/mL cholera toxin, and 10 μg/mL insulin, and passaged twice a week. The chemical compounds were reconstituted in DMSO.

For the MTT [3-(4,5-dimethylthiazol-2-yl)-2,5-diphenyltetrazolium bromide] assay, cells were seeded in a 96-well plate (26.000 cells/well) for 48 h. Then, after overnight starvation through serum deprivation, cells were incubated for 24 h, with the chemical compounds at 100, 10, 1, and 0.1 μM concentrations in the MCF-12A culture medium deprived of serum. Next, MTT (5 mg/mL in sterile PBS) was added in an amount of 10% in the culture media volume for 4-h cell incubation at 37 °C. Subsequently, an MTT solubilization solution was added, in an amount equal to the original culture media volume (100 μL/well), and the formazan crystals were dissolved through thorough pipetting. The spectrophotometric measure of cells’ viability was performed in a Tecan (SPARK) spectrophotometer at an absorbance wavelength of 570 nm. Incubation with H_2_O_2_ was established as cytotoxicity positive control. The percentage of viability for each sample was calculated with respect to cells incubated with MCF-12A culture media (without serum) containing 2% DMSO (negative cytotoxicity control).

## 4. Conclusions

Using the recently reported general approach for the liquid-phase fragment synthesis of orthogonally protected naturally occurring PAs [13], a collection of orthogonally protected PAs was synthesized and subsequently successfully applied to prepare ten analogs of the naturally occurring PATs **Agel 416**, **HO-416b**, and JSTX-3, isolated from spider venoms, and PhTX-433, isolated from wasp venoms. The antiproliferative activity of the thus-synthesized conjugates was evaluated in MCF-7 and the MDA-MB-231 breast cancer cell lines, using the PATs **Agel 416** and **HO-416b** as reference compounds. 

PATs **Agel-416**, **HO-416b**, and analog **2** thereof, with Spd as the PA chain, presented potent antiproliferative activity for both cancerous cell lines with IC_50_ values ranging from 0.09–3.15 μM (ΜCF-7) and 3.31–12.6 μM (MDA-MB-231). Two other compounds, namely the JSTX-3 analogs **9** and **10**, incorporating photoactivatable and clickable groups in their head group, were also potent inhibitors but only for the MCF-7 cell line (IC_50_ = 2.63–2.81 μM). All other analogs presented either low or very low antiproliferative activity. 

The present study reveals that both the structure of the lipophilic head group and the polyamine chain are of significant importance for the strength of the antiproliferative potency of the PATs and their selectivity towards different types of cancerous cells. On the other hand, the potent antiproliferative activity of JSTX-3 analogs for the MCF-7 cells make them attractive tools for identifying potential cellular macromolecular targets, which will help design more potent PAT-based antiproliferative agents.

The two most potent antiproliferative agents, **Agel 416** and **HO-416b**, in this cohort of compounds presented very low cytotoxicity, in the range of 97–184 μΜ, on the MCF-12A normal epithelial mammary cell line, with selectivity indexes (SI) in the ranges 335–1080 and 24–56 for the MCF-7 and the MDA-MB-231 cells, respectively. Therefore, both compounds, and especially **HO-416b**, can be safely used for further evaluation studies as potential PAT-based anticancer drugs. 

## Figures and Tables

**Table 1:** Antiproliferative activity of synthesized PATs and analogs in the MCF-7 and MDA-MB-231 breast cancer cell lines. SD values are also provided for each IC_50_ value.

		IC_50_ (μM)	
Compound	LogD ^1^	MCF-7	MDA-MB-231
**Agel 416**	−7.54	0.55 ± 0.02	3.31 ± 0.08
**HO-416b**	−6.25	0.09 ± 0.012	3.98 ± 0.064
**1**	−6.21	>200	>200
**2**	−2.84	3.15 ± 0.25	12.6 ± 0.57
**3**	−1.49	272.4 ± 11.2	107.2 ± 10.7
**4**	−6.66	>400	>400
**5**	−8.16	>400	>400
**6**	−5.97	>400	>400
**7**	−6.81	>400	>400
**8**	−6.05	>400	>400
**9**	−3.87	2.63 ± 1.58	>200
**10**	−5.34	2.81 ± 1.64	>200
**21**	−0.96	365.9 ± 73	262.2 ± 42
**21**+ Spd [**21**:Spd = 1:1]		145.6 ± 96	282.5 ± 55

^1^ LogD values were calculated using ChemAxon’s Chemicalize platform on 27 December 2021; https://chemicalize.com (accessed on 3 January 2022).

**Table 2:** The IC_50_ values of PATs **HO-416b** and **Agel 416** in MCF-12A normal epithelial mammary cells.

Compound	IC_50_ (μM)
**Agel 416**	184.14
**HO-416b**	97.24

**Table 3:** Gradient elution profile.

Time (min)	% MeCN/H_2_O
0.00	7.0
3.50	95.0
6.00	95.0
6.50	100.0
7.00	7.0

## Data Availability

Data not contained within this article can be found in the attached Supplementary Materials.

## Supplementary Materials

The following supporting information can be downloaded online, Copies of selected ^1^H- and ^13^C-NMR spectra, Diagrams of dose-dependent responses (DDR) of the tested compounds on MCF-7 and MDA-MB-231 breast cancer cells, Experimental protocols, Figure S1: Viability of MCF-12A cells after treatment with PATs **HO-416b** and **Agel 416**, Table S1: Structures and antiproliferative activity (IC_50_ values) for tested compounds, Table S2: Structures and IC_50_ values for the effect of PATs **Agel 416** and **HO-416b** on the viability of MCF-12A cells.
