# Supplementary material for: New Analogs of Polyamine Toxins from Spiders and Wasps: Liquid Phase Fragment Synthesis and Evaluation of Antiproliferative Activity
Source: Molecules. 2022 Jan 10;27(2):447. doi: 10.3390/molecules27020447 (PMC8777946; doi:10.3390/molecules27020447)
Supplement: Supplementary file 1 [file molecules-27-00447-s001.zip › molecules-1527612-supplementary.pdf]

Supplementary Material

New Analogs of Polyamine Toxins from Spiders and Wasps: Liquid Phase Fragment Synthesis and Evaluation of Antiproliferative Activity

Table of Contents

1. Copies of selected ^{1}H- and ^{13}C-NMR spectra. S5

1.1 Copy of ^{1}H-NMR spectrum of N^4-nosyl-N^1-tritylspermidine……………………S5

1.2 Copy of ^{13}C-NMR spectrum of N^4-nosyl-N^1-tritylspermidine..............................S5

1.3 Copy of ^{1}H-NMR spectrum of compound **13**……………………………………S6

1.4 Copy of ^{13}C-NMR spectrum of compound **13**…………………………………...S6

1.5 Copy of ^{1}H-NMR spectrum of N^4-Nosyl-N^1-tritylnorspermidine.........................S7

1.6 Copy of ^{13}C-NMR spectrum of N^4-Nosyl-N^1-tritylnorspermidine.......................S7

1.7 Copy of ^{1}H-NMR spectrum of N^4,N^7-Dinosyl-N^1-tritylnorspermidine …………S8

1.8 Copy of ^{13}C-NMR spectrum of N^4,N^7-Dinosyl-N^1-tritylnorspermidine ………...S8

1.9 Copy of ^{1}H-NMR spectrum of compound **15**…………………….....................S9

1.10 Copy of ^{1}H-NMR spectrum of compound **23**…………………………………S10

1.11 Copy of ^{13}C-NMR spectrum of compound **23**………………………………..S10

1.12 Copy of ^{1}H-NMR spectrum of compound **29**..S11

1.13 Copy of ^{13}C-NMR spectrum of compound **29**...S11

1.14 Copy of ^{1}H-NMR spectrum of N-(4-bromobutyl)phthalimide………………..S12

1.15 Copy of ^{13}C-NMR spectrum of N-(4-bromobutyl)phthalimide ………………S12

1.16 Copy of ^{1}H-NMR spectrum of compound **31**..S13

1.17 Copy of ^{13}C-NMR spectrum of compound **31**………………………………...S13

1.18 Copy of ^{1}H-NMR spectrum of **PAT** analog **1**………………………………...S14

1.19 Copy of ^{13}C-NMR spectrum of **PAT** analog **1**..S14

1.20 Copy of ^{1}H-NMR spectrum of compound **32**..S15

1.21 Copy of ^{13}C-NMR spectrum of compound **32**………………………………...S15

1.22 Copy of ^{1}H-NMR spectrum of compound **33**……………………………………S16
1.23 Copy of ^{1}H-NMR spectrum of compound **34**……………………………………S17
1.24 Copy of ^{13}C-NMR spectrum of compound **34**…………………………………...S17
1.25 Copy of ^{1}H-NMR spectrum of **PAT** analog **2**...S18
1.26 Copy of ^{13}C-NMR spectrum of **PAT** analog **2**..S18
1.27 Copy of ^{1}H-NMR spectrum of compound **35**……………………………………S19
1.28 Copy of ^{13}C-NMR spectrum of **35**…………………………………………………S19
1.29 Copy of ^{1}H-NMR spectrum of **PAT** analog **3**...S20
1.30 Copy of ^{13}C-NMR spectrum of **PAT** analog **3**………………………………...S20
1.31 Copy of^{1}H-NMR spectrum of compound **36**..S21
1.32 Copy of ^{1}H-NMR spectrum of compound **37**……………………………………S22
1.33 Copy of ^{13}C-NMR spectrum of compound **37**...S22
1.34 Copy of ^{1}H -NMR spectrum of compound **39**…………………………………...S23
1.35 Copy of ^{13}C-NMR spectrum of compound **39**…………………………………...S23
1.36 Copy of ^{1}H-NMR spectrum of compound **53**...S24
1.37 Copy of ^{13}C-NMR spectrum of compound **53**…………………………………...S24
1.38 Copy of ^{1}H -NMR spectrum of compound **56**…………………………………...S25
1.39 Copy of ^{13}C-NMR spectrum of compound **56**…………………………………...S25
1.40 Copy of ^{1}H-NMR spectrum of compound **45**...S26
1.41 Copy of ^{13}C-NMR spectrum of compound **45**…………………………………...S26
1.42 Copy of ^{1}H -NMR spectrum of compound **48**…………………………………...S27
1.43 Copy of ^{13}C-NMR spectrum of compound **48**…………………………………...S27
1.44 Copy of ^{1}H-NMR spectrum of PAT analog **9**...S28
1.45 Copy of ^{13}C-NMR spectrum of PAT analog **9**…………………………………...S28
1.46 Copy of ^{1}H -NMR spectrum of compound **49**…………………………………...S29
1.47 Copy of ^{13}C-NMR spectrum of compound **49**…………………………………...S29
1.48 Copy of ^{1}H-NMR spectrum of compound **52**...S30
1.49 Copy of ^{13}C-NMR spectrum of compound **52**…………………………………...S30
1.50 Copy of ^{1}H -NMR spectrum of PAT analog **10**……………………………….S31
1.51 Copy of ^{13}C-NMR spectrum of PAT analog **10**……………………………….S31
1.52 Copy of ^{1}H -NMR spectrum of compound **61**…………………………………...S32
1.53 Copy of ^{13}C-NMR spectrum of compound **61**…………………………………...S32
1.54 Copy of ^{1}H-NMR spectrum of compound **66**...S33
1.55 Copy of ^{13}C-NMR spectrum of compound **66**…………………………………...S33

1.56 Copy of ^{1}H -NMR spectrum of compound **72**……………………………...S34

1.57 Copy of ^{13}C-NMR spectrum of compound **72**……………………………...S34

2. Dose-dependent responses (DDR) diagrams. S35

2.1 DDR diagram for compound Agel 416x5CF_3CO_2H for the MCF-7 cells……...S35

2.2 DDR diagram for compound Agel 416x5CF_3CO_2H for the MDA-MB-231 cells……………………………………………………………………..S35

2.3 DDR diagram for compound HO-416bx5CF_3CO_2H for the MCF-7 cells……...S36

2.4 DDR diagram for compound HO-416bx5CF_3CO_2H for the MDA-MB-231 cells…...S36

2.5 DDR diagram for compound **1**x4CF_3CO_2H for the MCF-7 cells………………S37

2.6 DDR diagram for compound **1**x4CF_3CO_2H for the MDA-MB-231 cells……...S37

2.7 DDR diagram for compound **2**x3CF_3CO_2H for the MCF-7 cells………………S38

2.8 DDR diagram for compound **2**x3CF_3CO_2H for the MDA-MB-231 cells……...S38

2.9 DDR diagram for compound **3**x2CF_3CO_2H for the MCF-7 cells………………S39

2.10 DDR diagram for compound **3**x2CF_3CO_2H for the MDA-MB-231 cells…….S39

2.11 DDR diagram for acid **21** for the MCF-7 cells……………………………… S40

2.12 DDR diagram for acid **21** for the MDA-MB-231 cells……………………… S40

2.13 DDR diagram for acid **21**+Spd (1:1) for the MCF-7 cells…………………....S41

2.14 DDR diagram for acid **21**+Spd (1:1) for the MDA-MB-231 cells……………S41

2.15 DDR diagram for compound **4**x3CF_3CO_2H for the MCF-7 cells……………..S42

2.16 DDR diagram for compound **4**x3CF_3CO_2H for the MDA-MB-231 cells…......S42

2.17 DDR diagram for compound **5**x3CF_3CO_2H for the MCF-7 cells……………..S43

2.18 DDR diagram for compound **5**x3CF_3CO_2H for the MDA-MB-231 cells…......S43

2.19 DDR diagram for compound **9**x3CF_3CO_2H for the MCF-7 cells……………..S44

2.20 DDR diagram for compound **9**x3CF_3CO_2H for the MDA-MB-231 cells…......S44

2.21 DDR diagram for compound **10**x3CF_3CO_2H for the MCF-7 cells……………S45

2.22 DDR diagram for compound **10**x3CF_3CO_2H for the MDA-MB-231 cells…...S45

2.23 DDR diagram for compound **6**x3CF_3CO_2H for the MCF-7 cells……………..S46

2.24 DDR diagram for compound **6**x3CF_3CO_2H for the MDA-MB-231 cells…......S46

2.25 DDR diagram for compound **7**x3CF_3CO_2H for the MCF-7 cells…………......S47

2.26 DDR diagram for compound **7**x3CF_3CO_2H for the MDA-MB-231 cells…......S47

2.27 DDR diagram for compound **8**x4CF_3CO_2H for the MCF-7 cells……………..S48

2.28 DDR diagram for compound **8**x4CF_3CO_2H for the MDA-MB-231 cells…......S48

3. Experimental protocols……………………………………………………………S49

4. Table S1. Structures and antiproliferative activity (IC_{50} values) for tested compounds……………………………………………………………………………S75

5. Figure S1: Viability of MCF-12A cells following treatment with PATs Agel 416 and HO-416b …….……………………………………………………………..S77

6. Table S2: Structures and IC_{50} values for the effect of PATs Agel 416 and HO-416b on the viability of MCF-12A cells………………………………………..S78

1. Copies of selected ^{1}H- and ^{13}C-NMR spectra

1.1 ^{1}H-NMR spectrum of *N*^{4}-nosyl-*N*^{1}-tritylspermidine

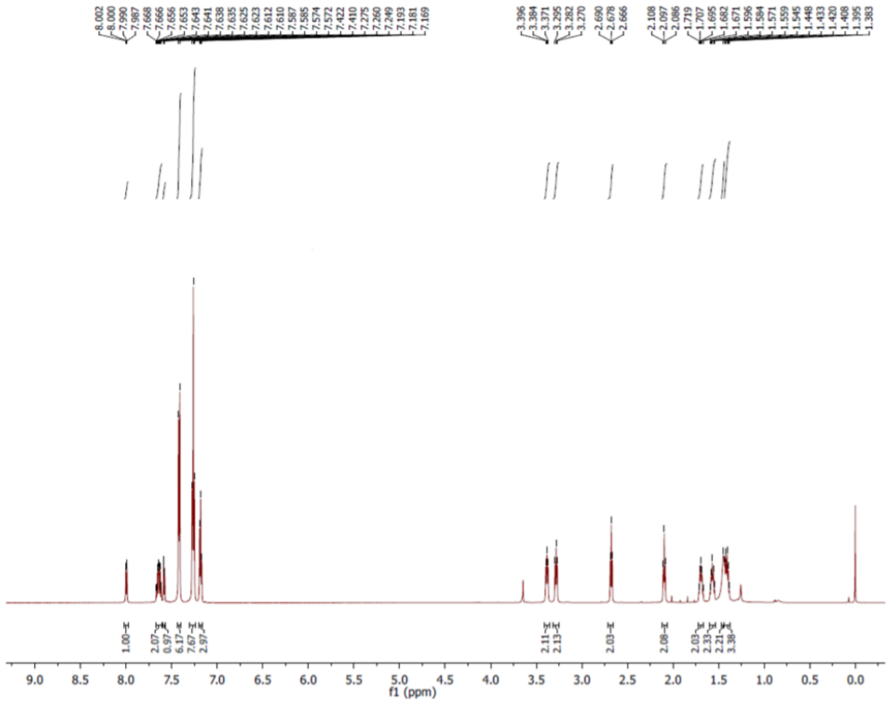

1.2 ^{13}C-NMR spectrum of *N*^{4}-nosyl-*N*^{1}-tritylspermidine

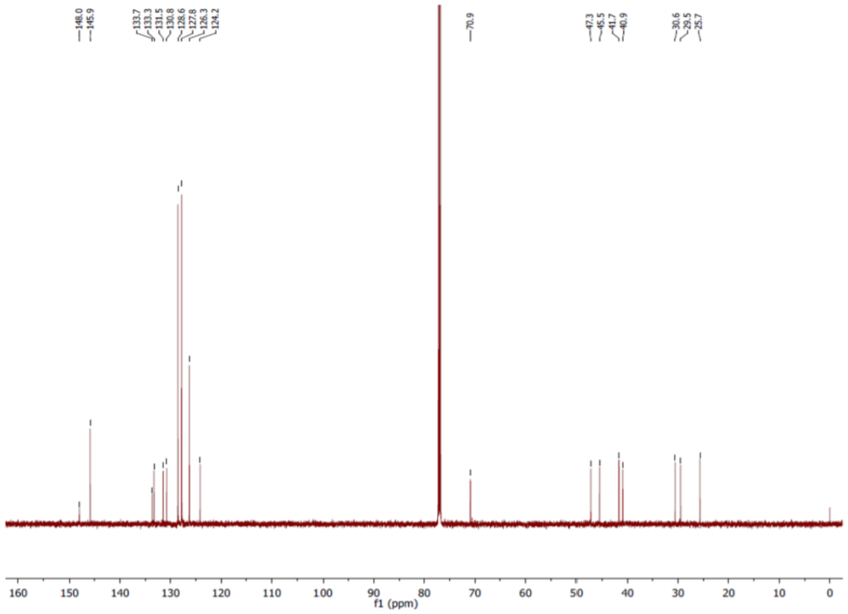

1.3 ^{1}H-NMR spectrum of compound 13

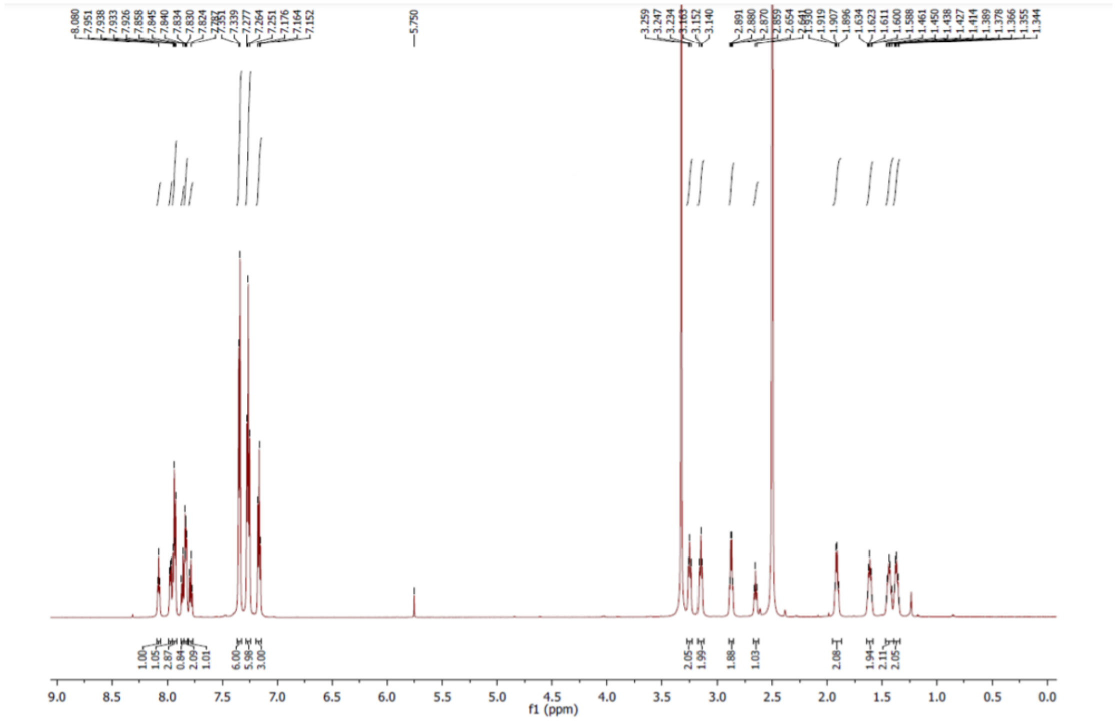

1.4 ^{13}C-NMR spectrum of compound 13

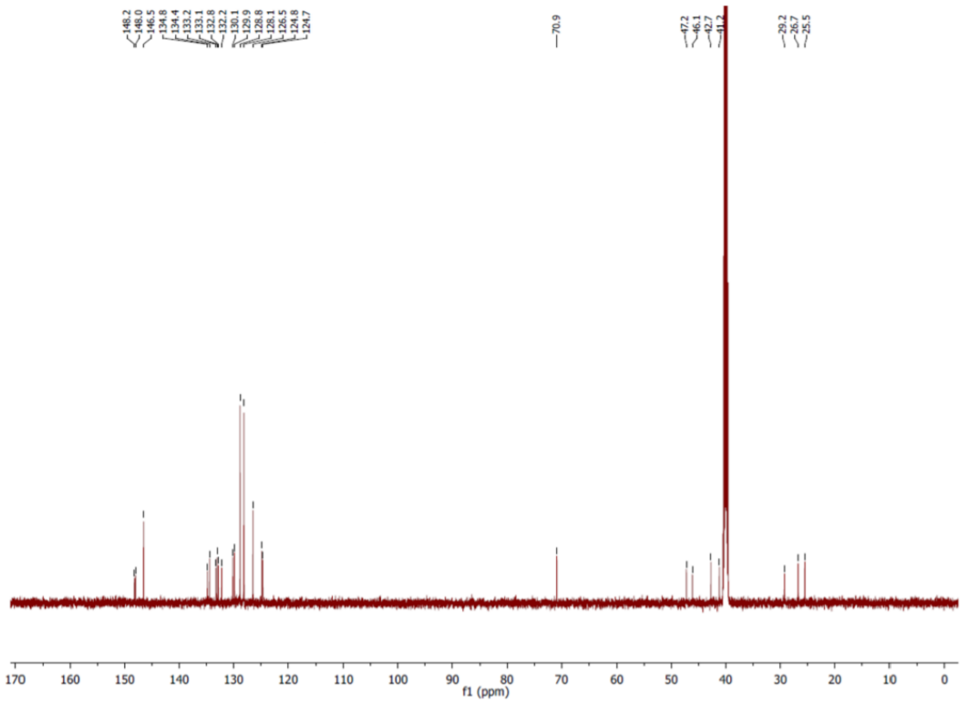

1.5 ^{1}H-NMR spectrum of *N*^{4}-Nosyl-*N*^{1}-tritylnorspermidine

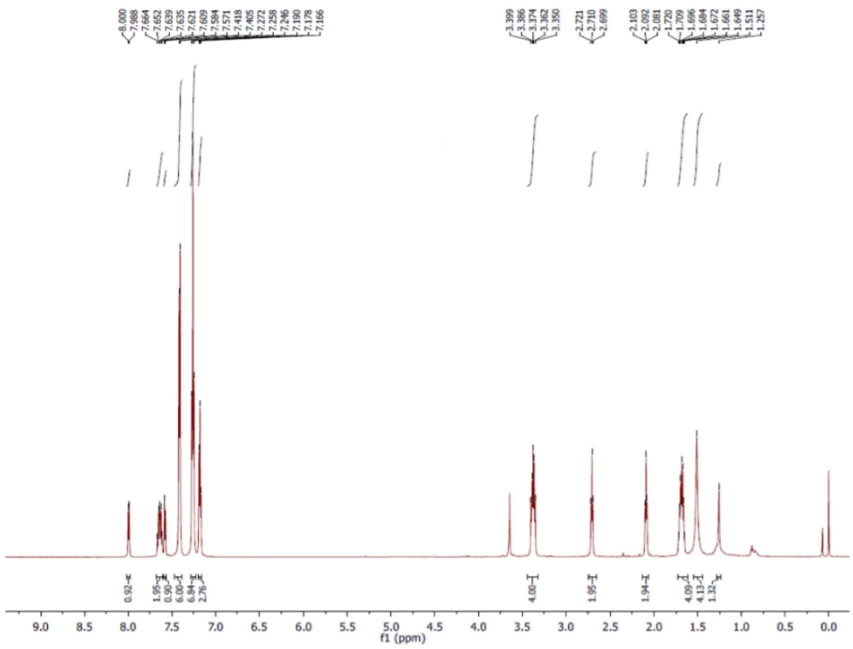

1.6 ^{13}C-NMR spectrum of *N*^{4}-Nosyl-*N*^{1}-tritylnorspermidine

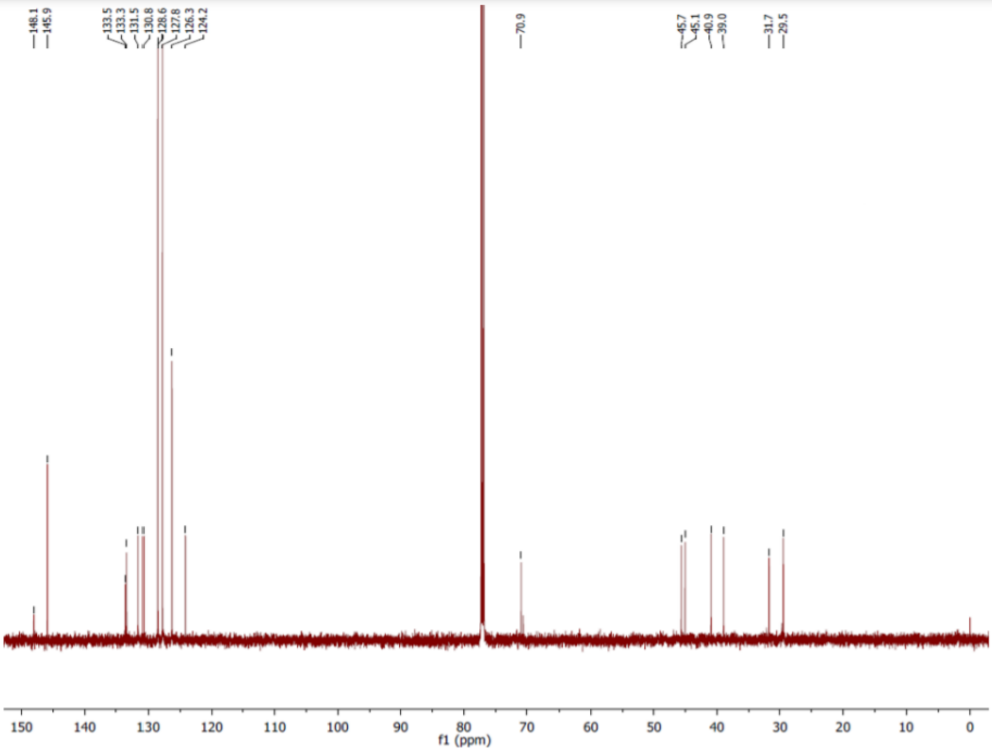

1.7 ^{1}H-NMR spectrum of *N*^{4},*N*^{7}-Dinosyl-*N*^{1}-tritylnorspermidine

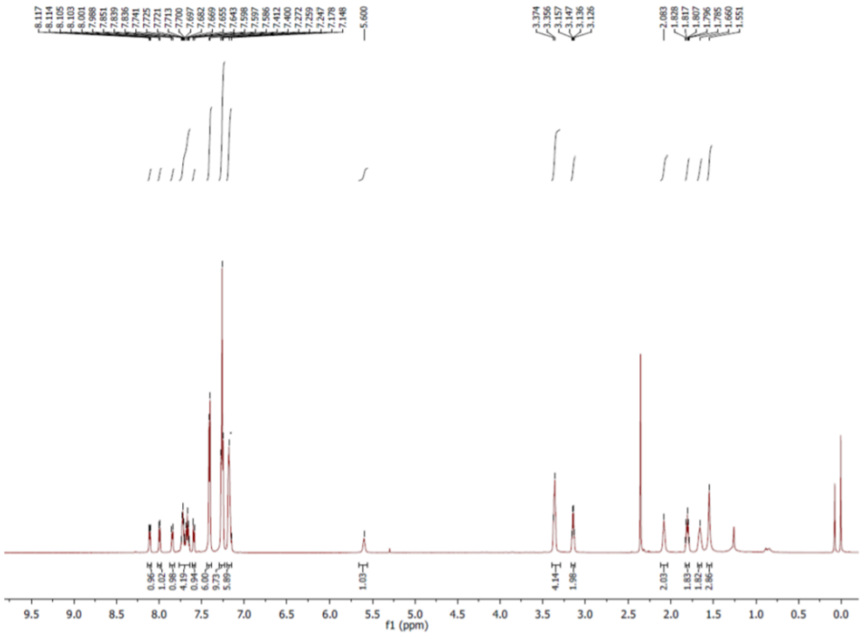

1.8 ^{13}C-NMR spectrum of *N*^{4},*N*^{7}-Dinosyl-*N*^{1}-tritylnorspermidine

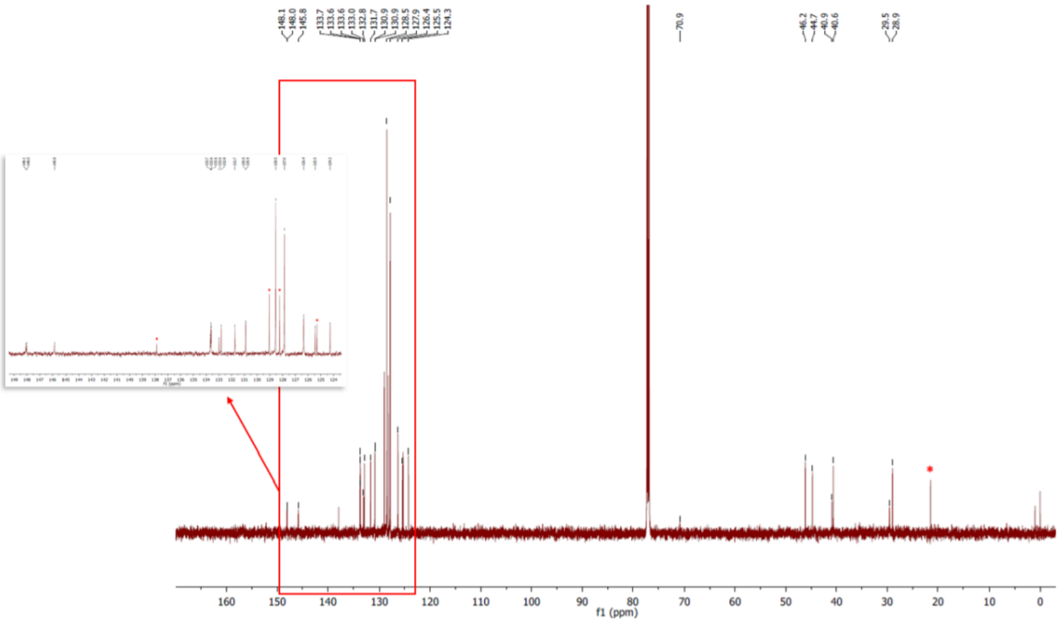

*Residual solvent

1.9 ^{1}H-NMR spectrum of compound 15

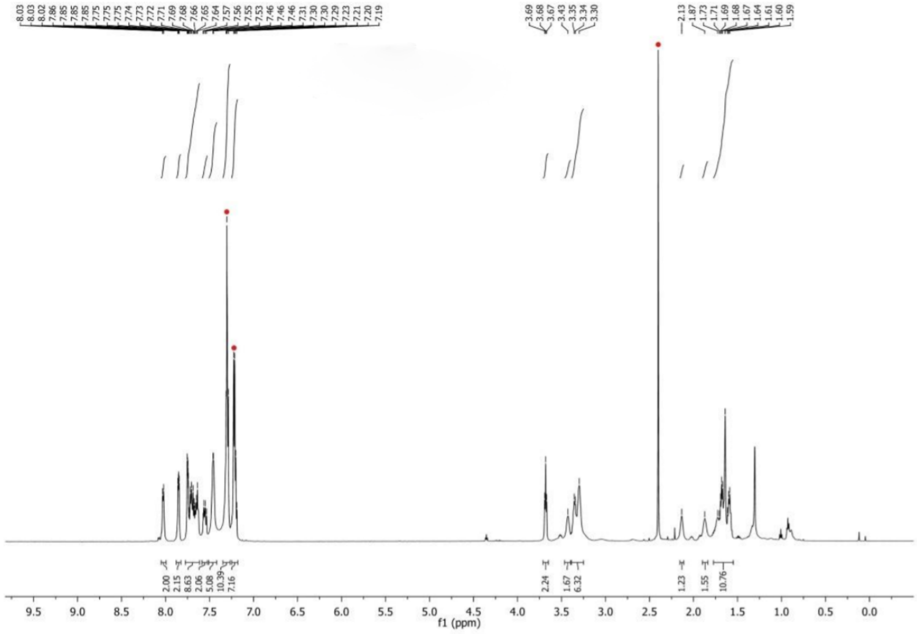

1.10 ^{1}H-NMR spectrum of compound 23

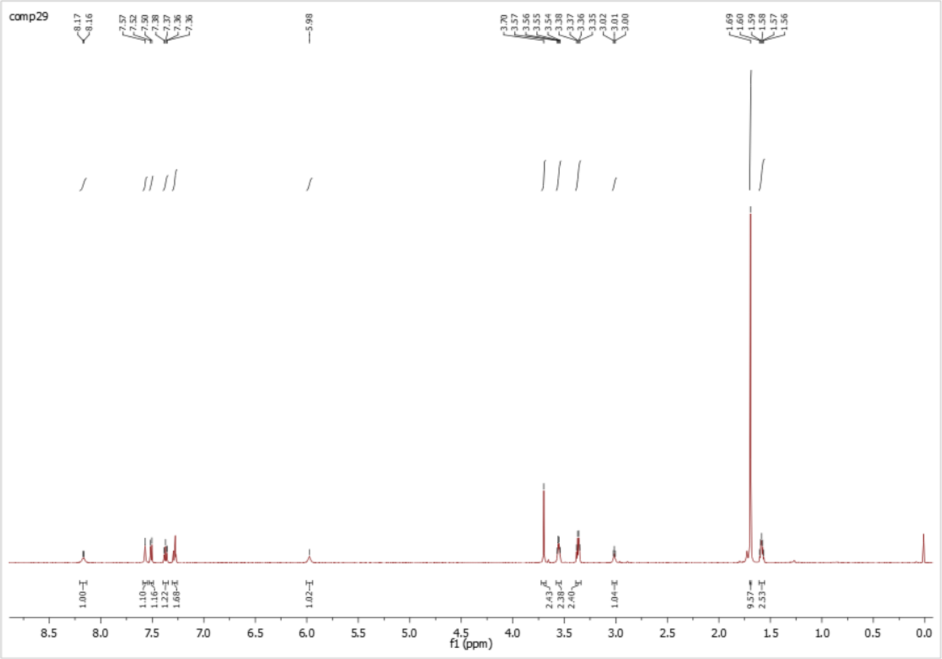

1.11 ^{13}C-NMR spectrum of compound 23

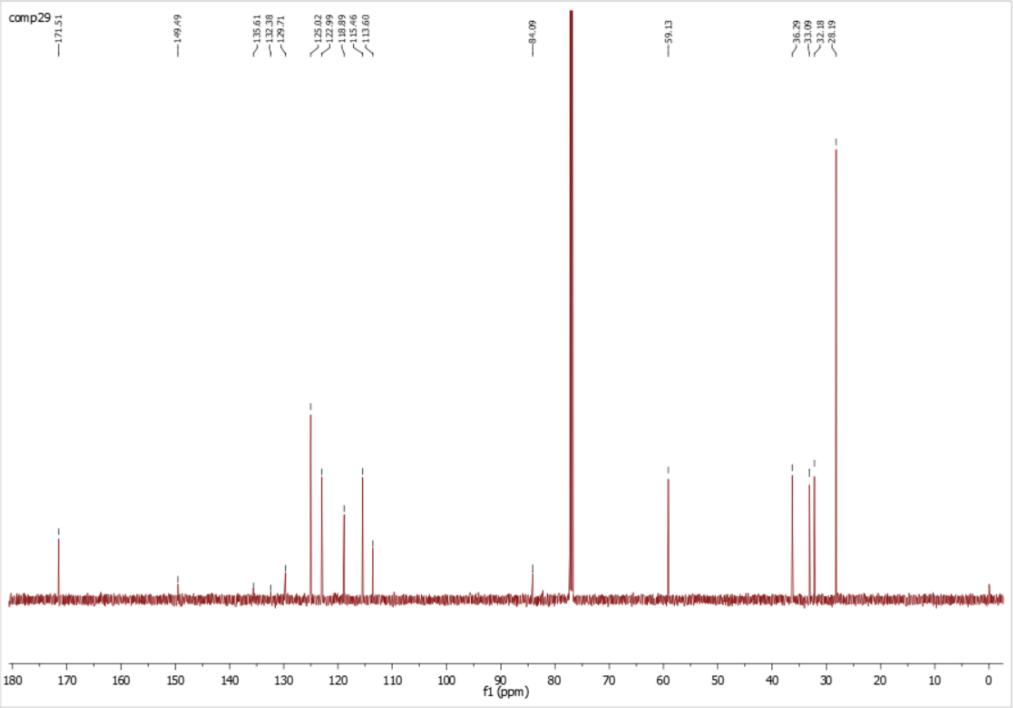

1.12 ^{1}H-NMR spectrum of compound 29

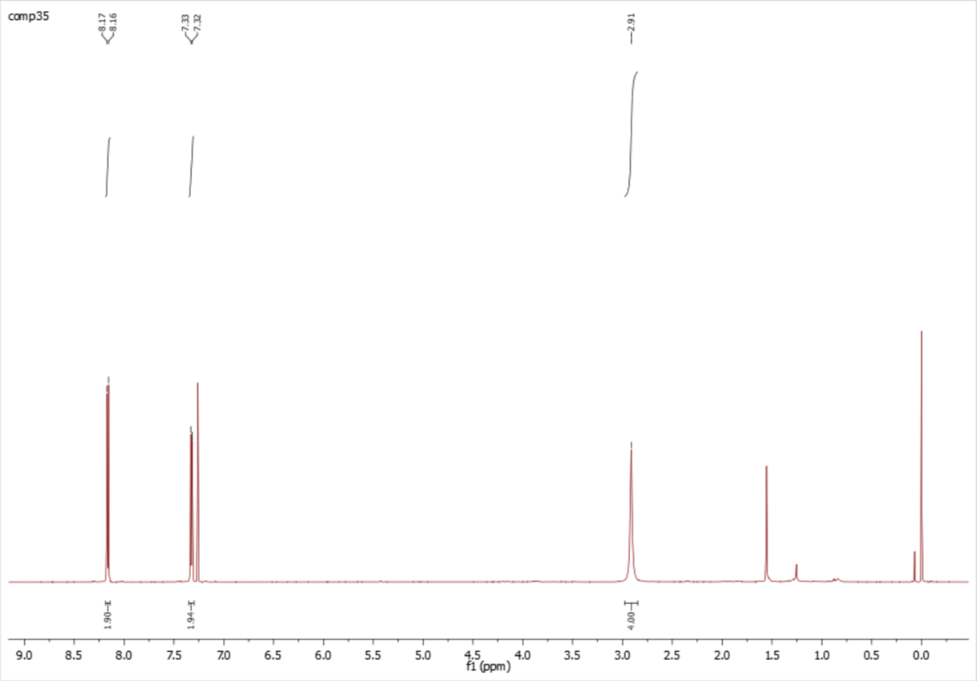

1.13 ^{13}C-NMR spectrum of compound 29

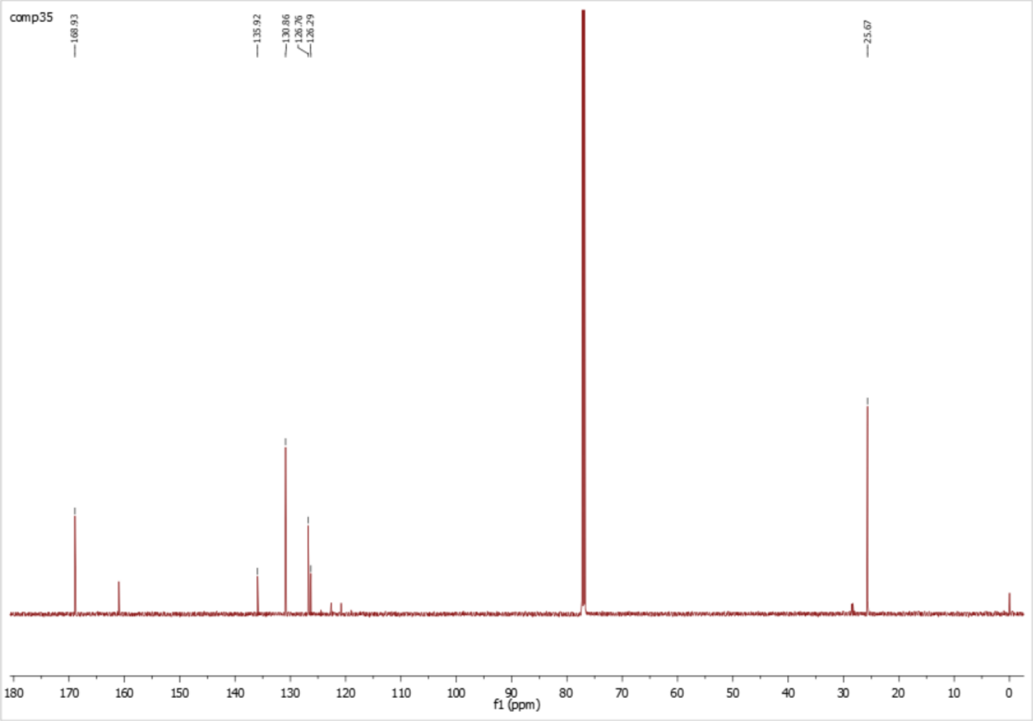

1.14 ^{1}H-NMR spectrum of *N*-(4-bromobutyl)phthalimide

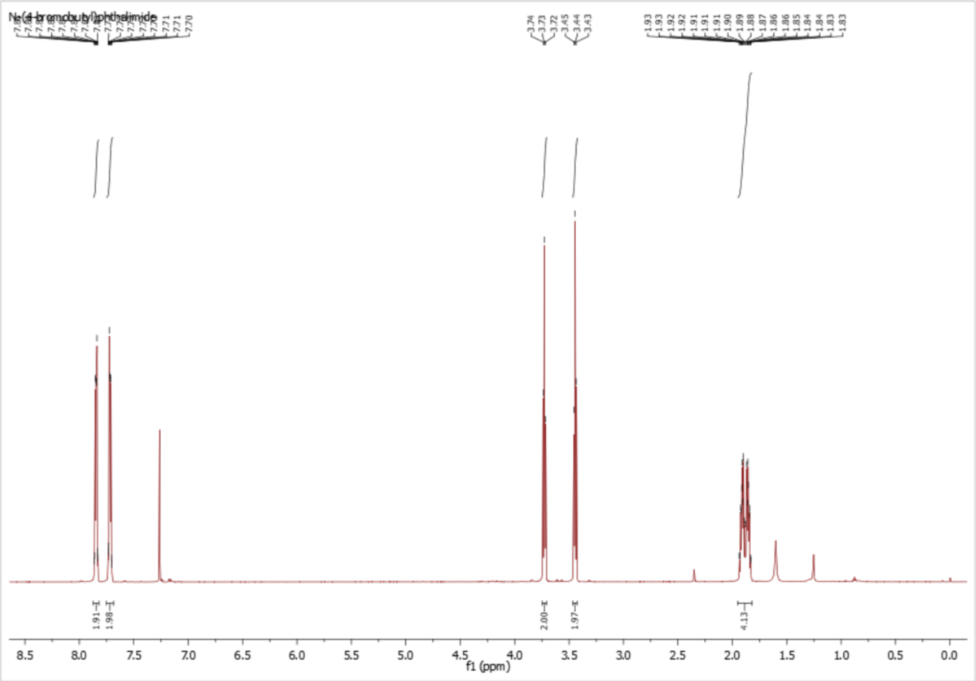

1.15 ^{13}C-NMR spectrum of *N*-(4-bromobutyl)phthalimide

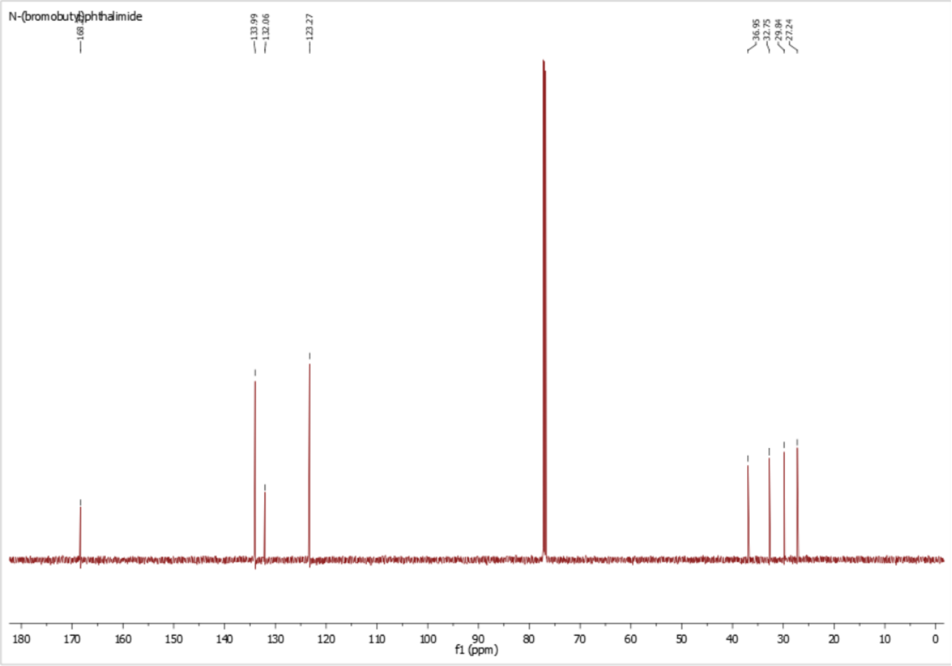

1.16 ^{1}H-NMR spectrum of compound 31

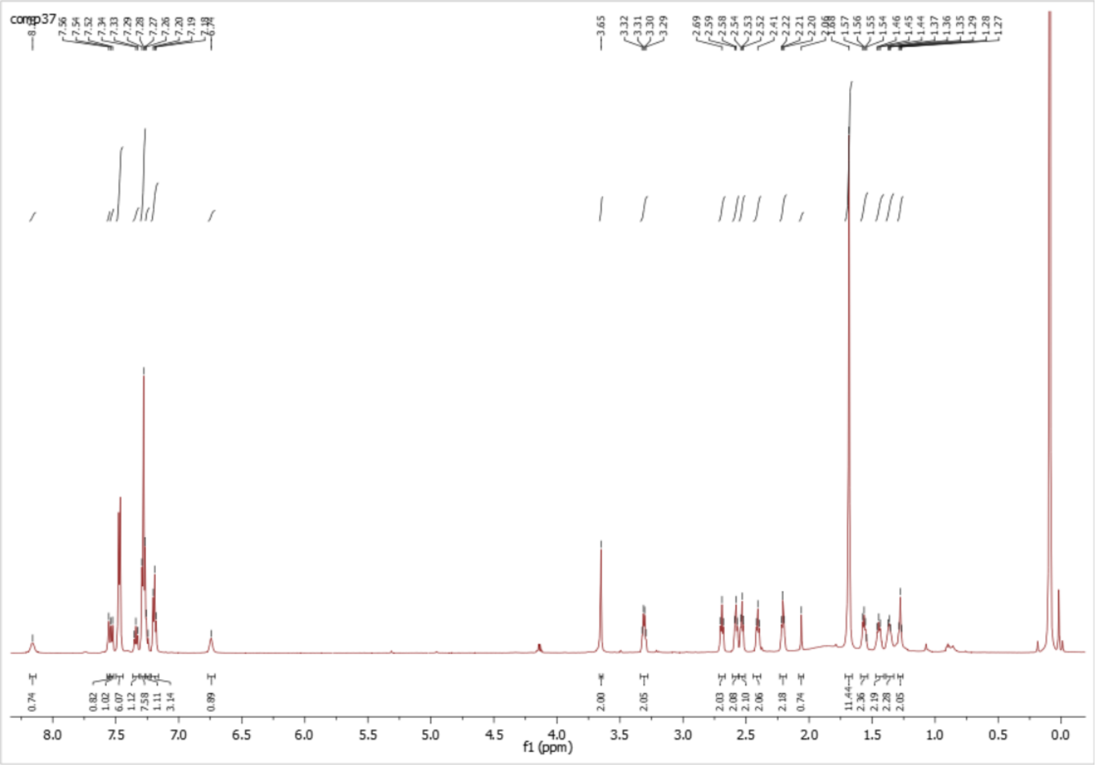

1.17 ^{13}C-NMR spectrum of compound 31

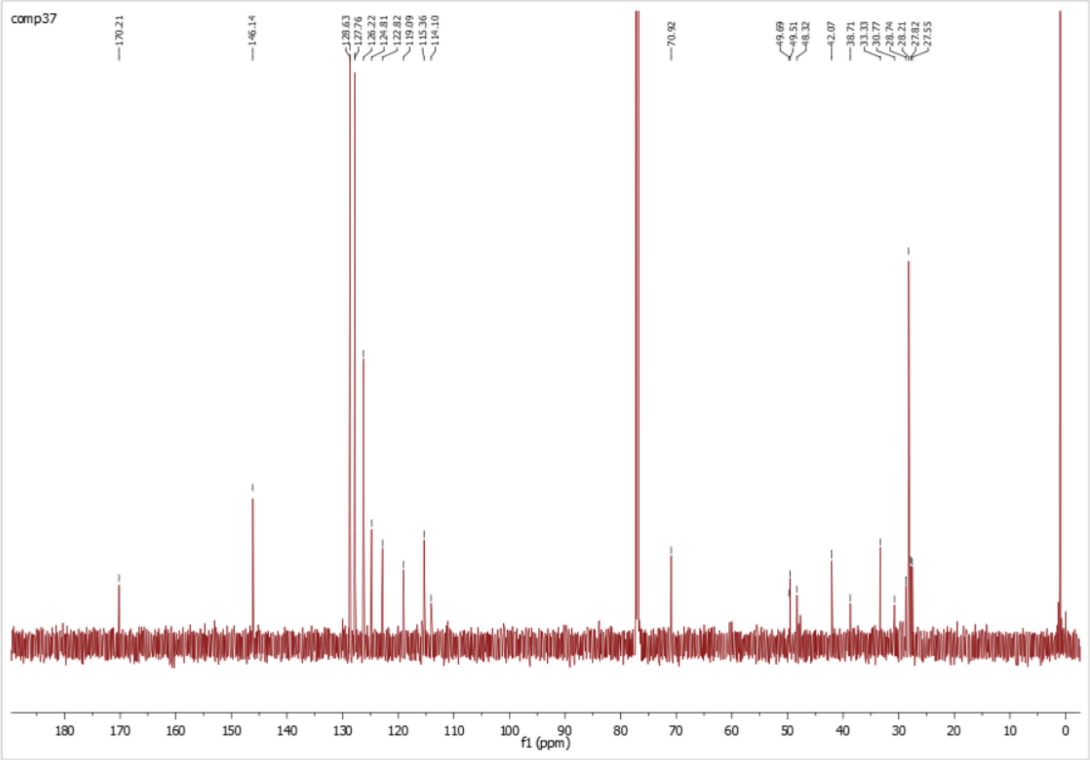

1.18 ^{1}H-NMR spectrum of PAT analog 1

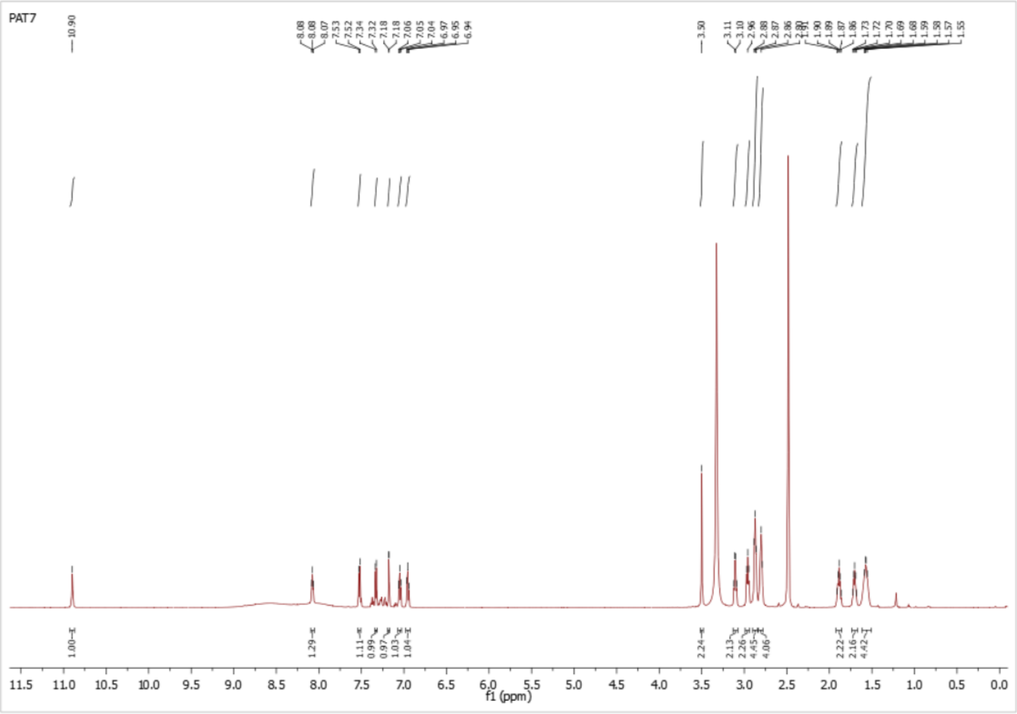

1.19 ^{13}C-NMR spectrum of PAT analog 1

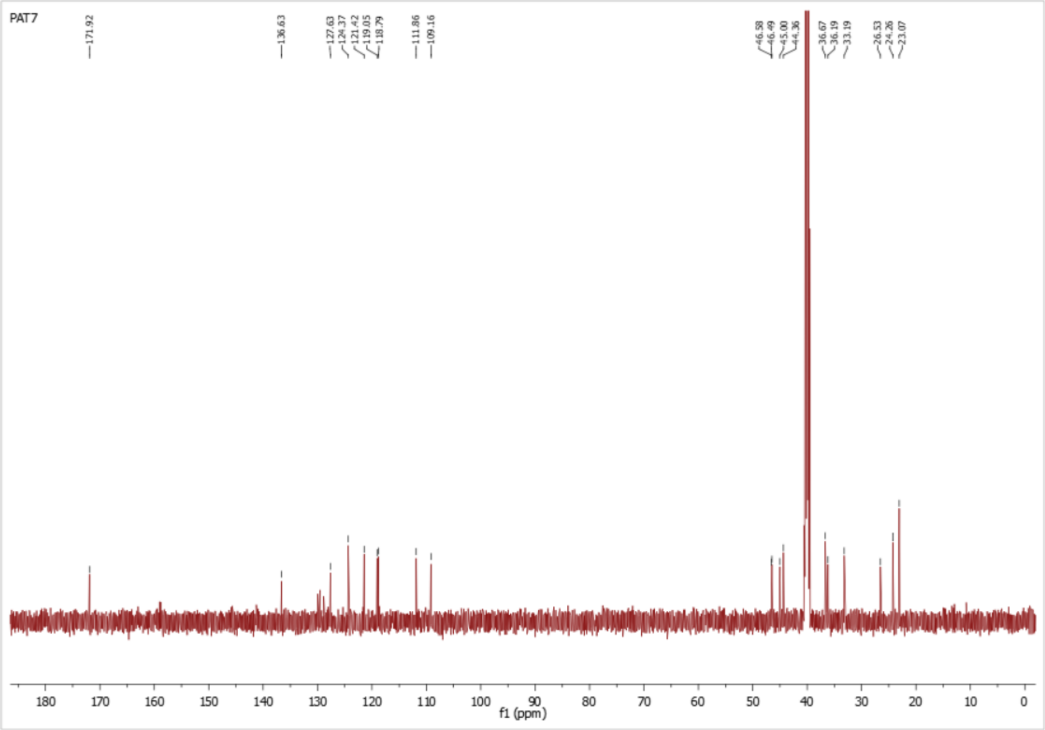

1.20 ^{1}H-NMR spectrum of compound 32

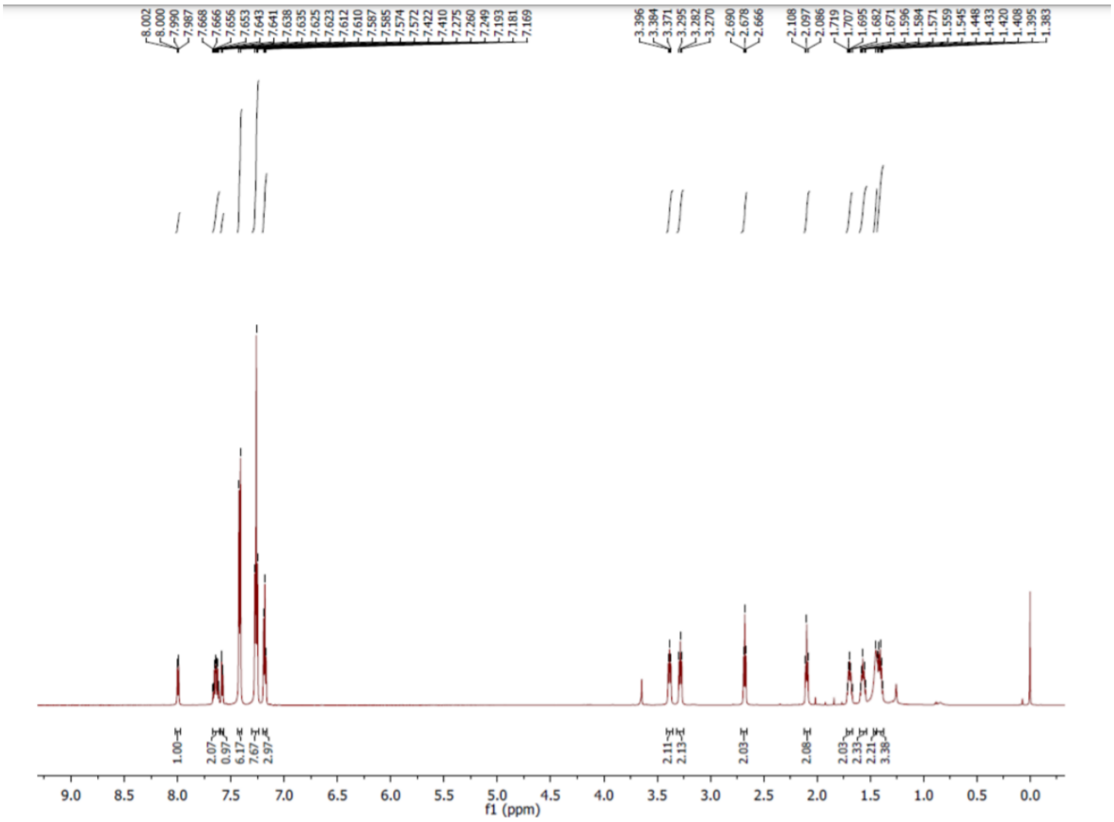

2.21 ^{13}C-NMR spectrum of compound 32

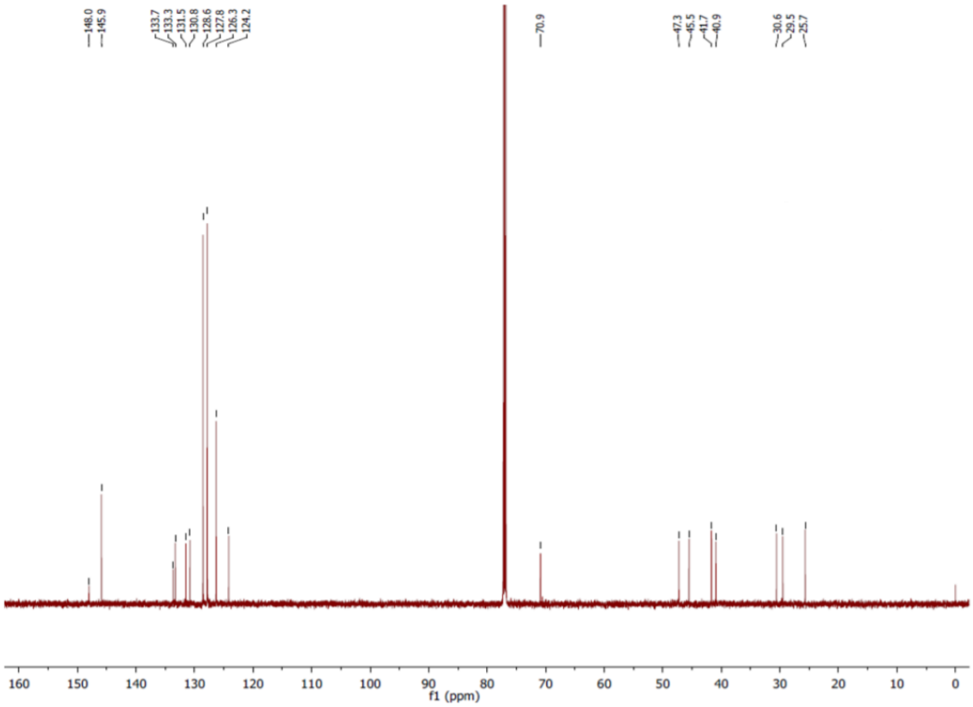

1.22 ^{1}H-NMR spectrum of compound 33

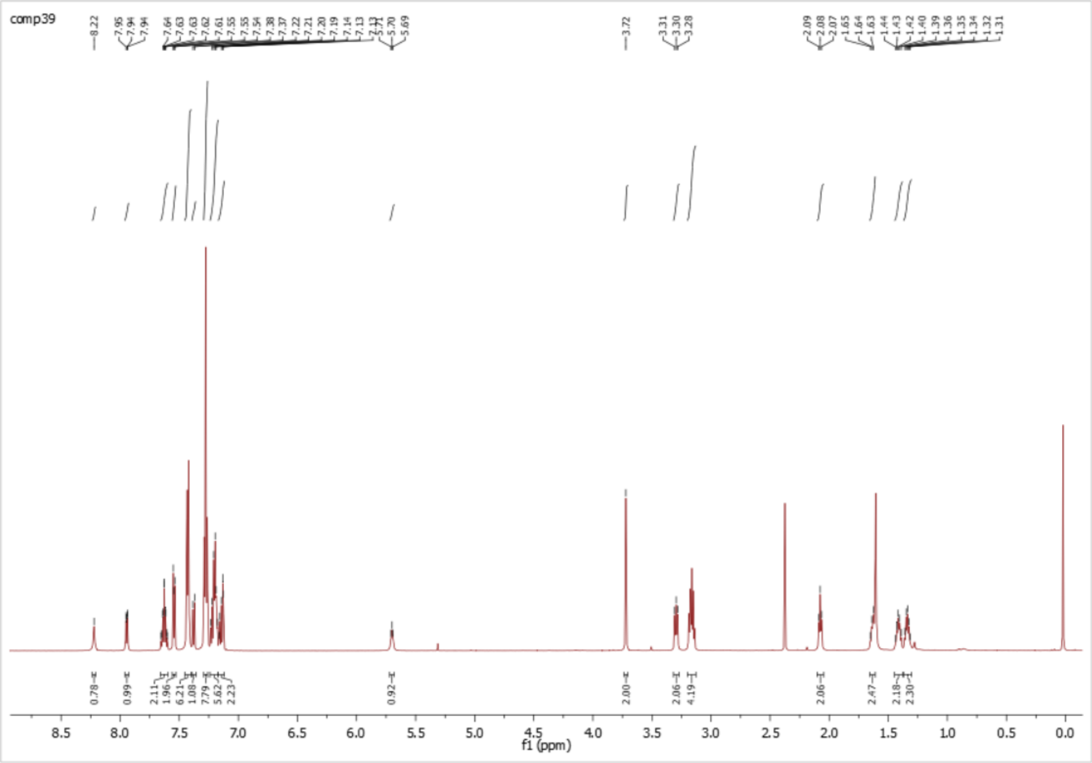

1.23 ^{1}H-NMR spectrum of compound 34

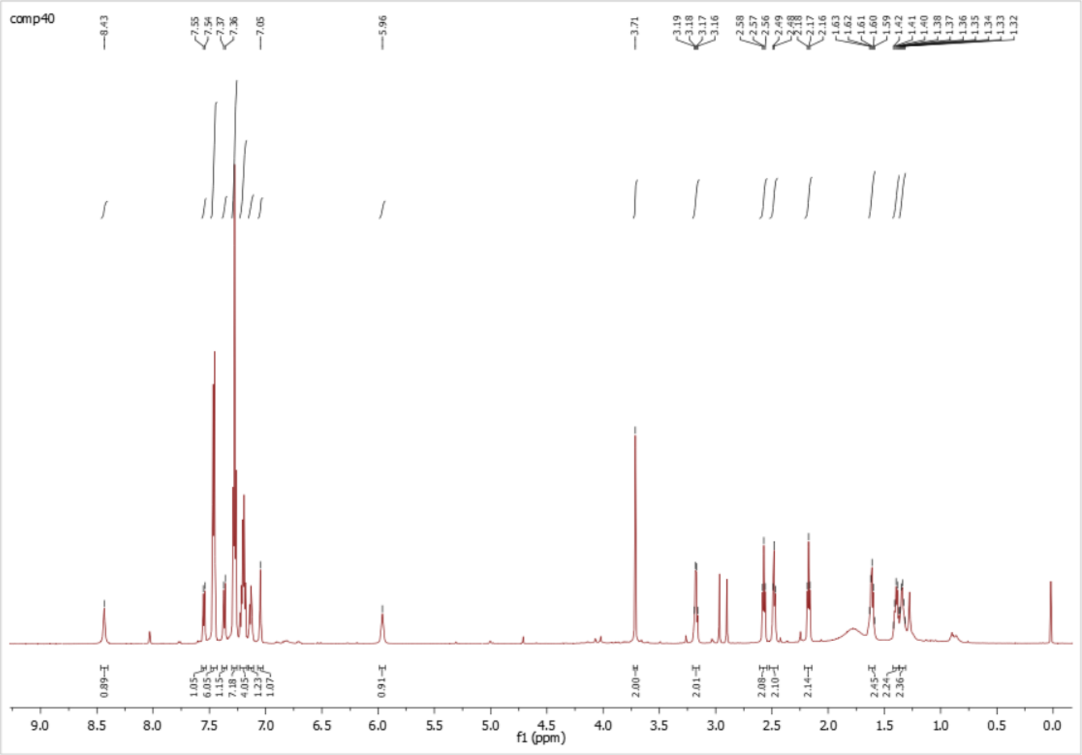

1.24 ^{13}C-NMR spectrum of compound 34

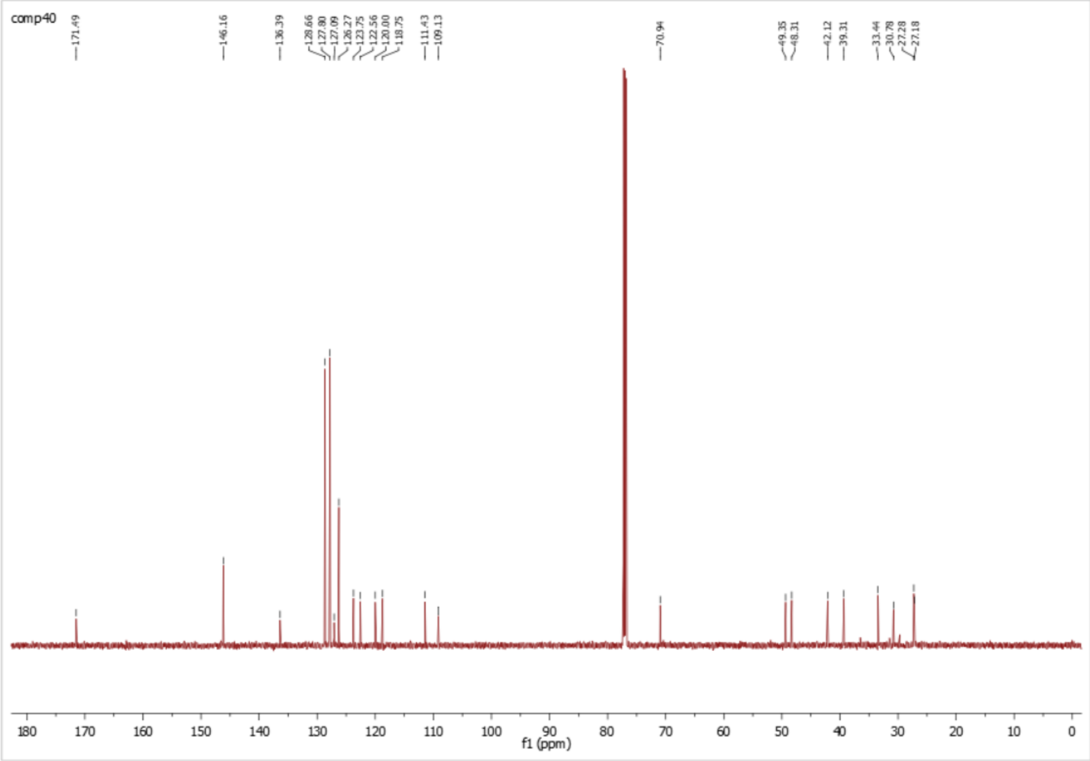

1.25 ^{1}H-NMR spectrum of PAT analog 2

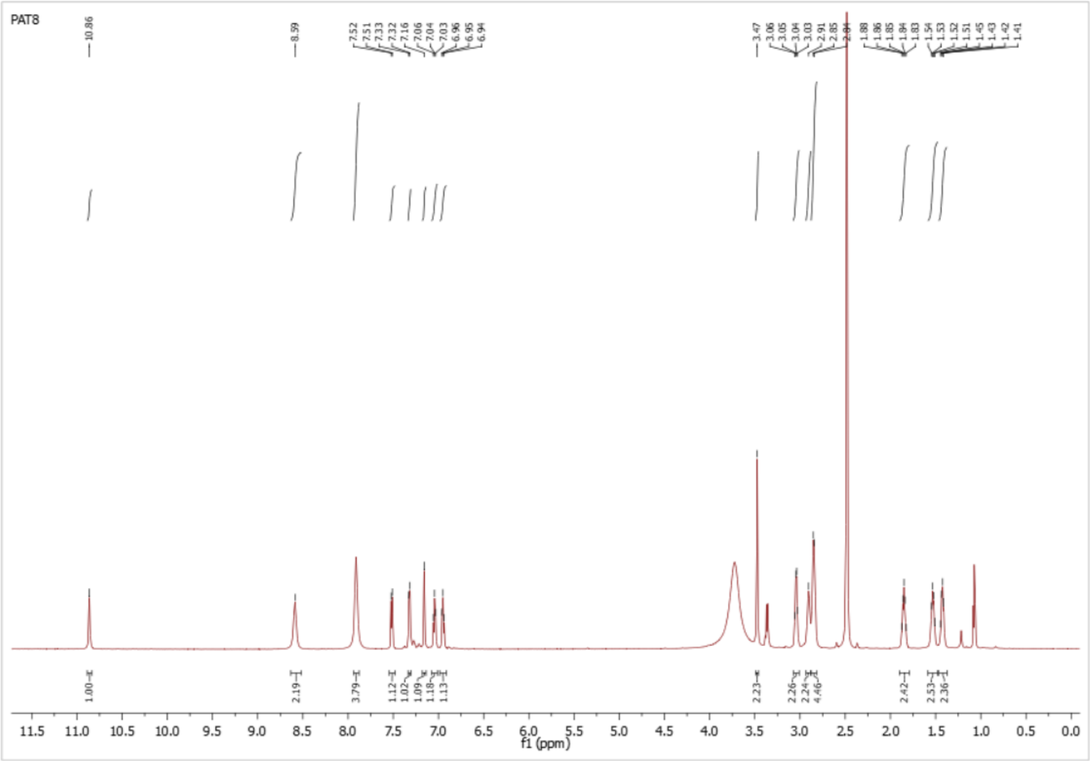

1.26 ^{13}C-NMR spectrum of PAT analog 2

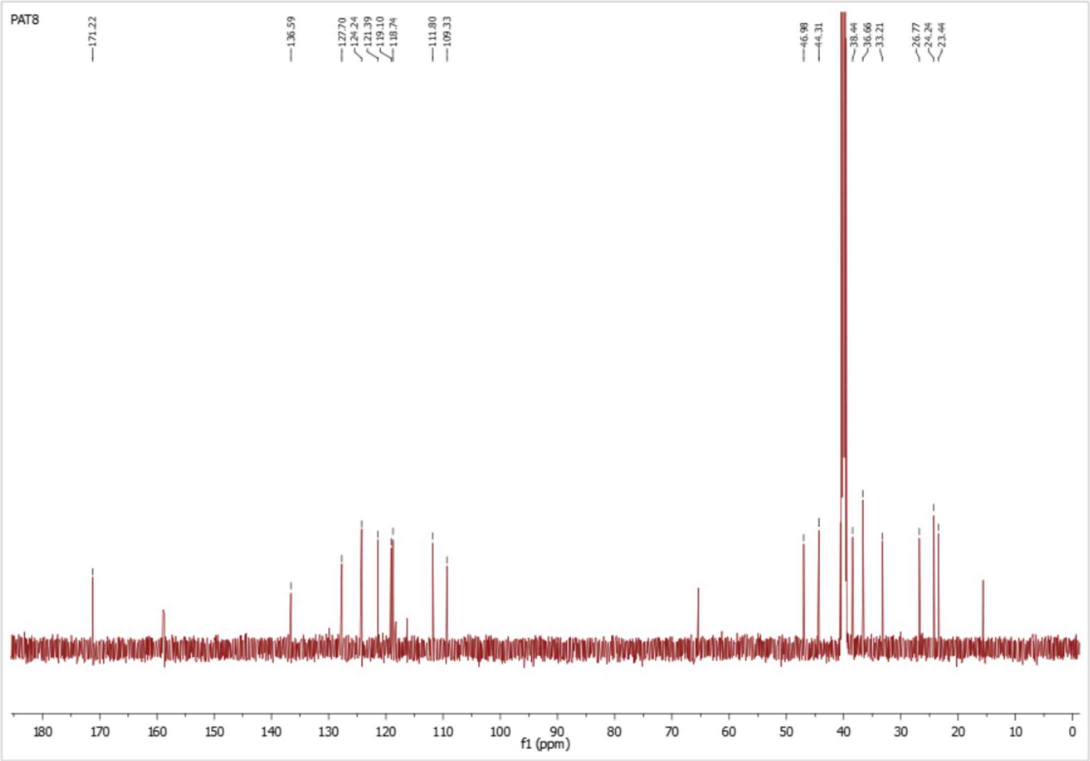

1.27 ^{1}H-NMR spectrum of compound 35

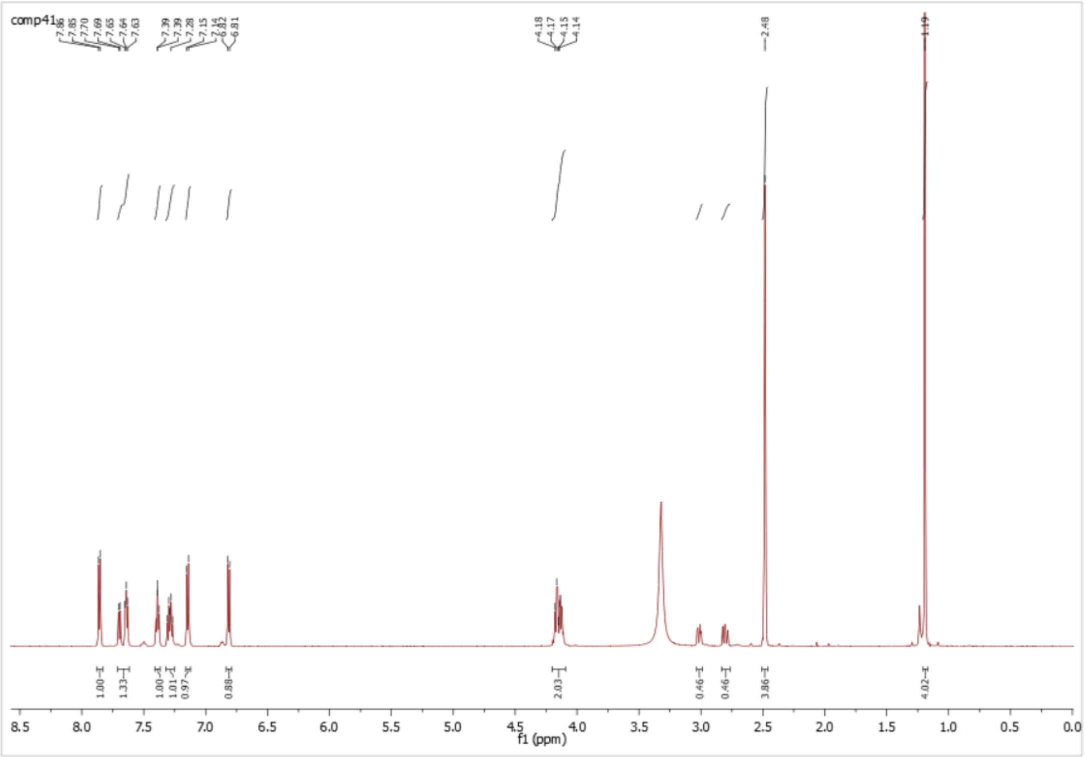

1.28 ^{13}C-NMR spectrum of 35

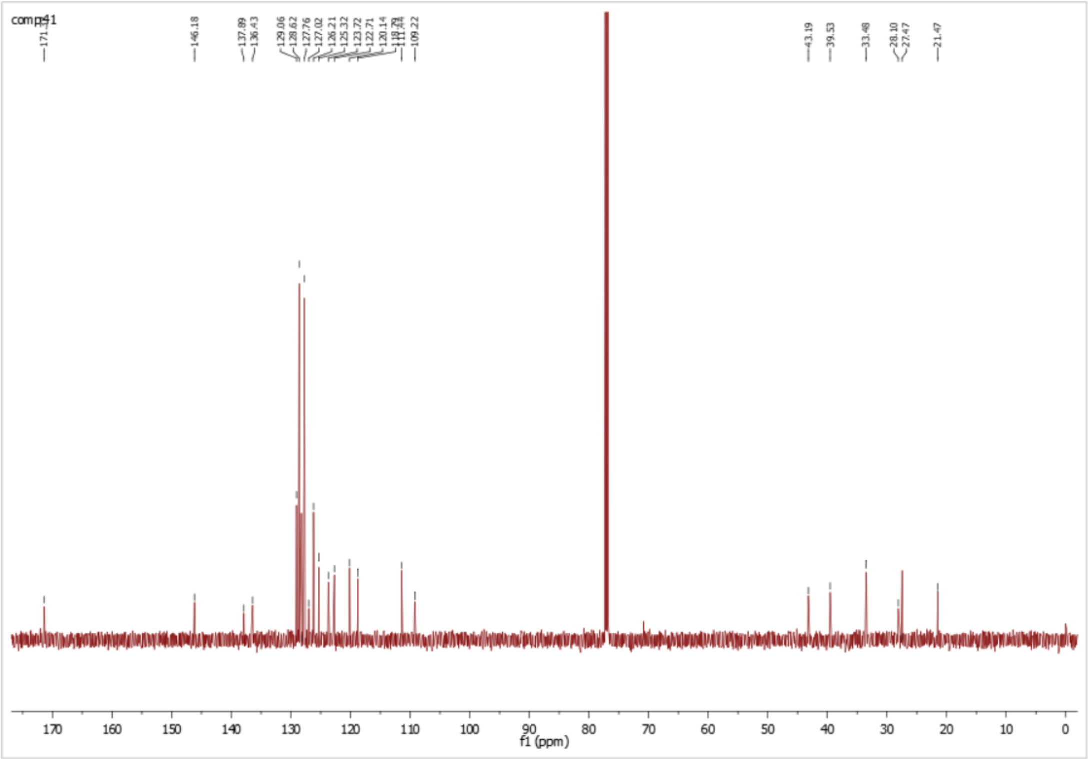

1.29 ^{1}H-NMR spectrum of PAT analog 3

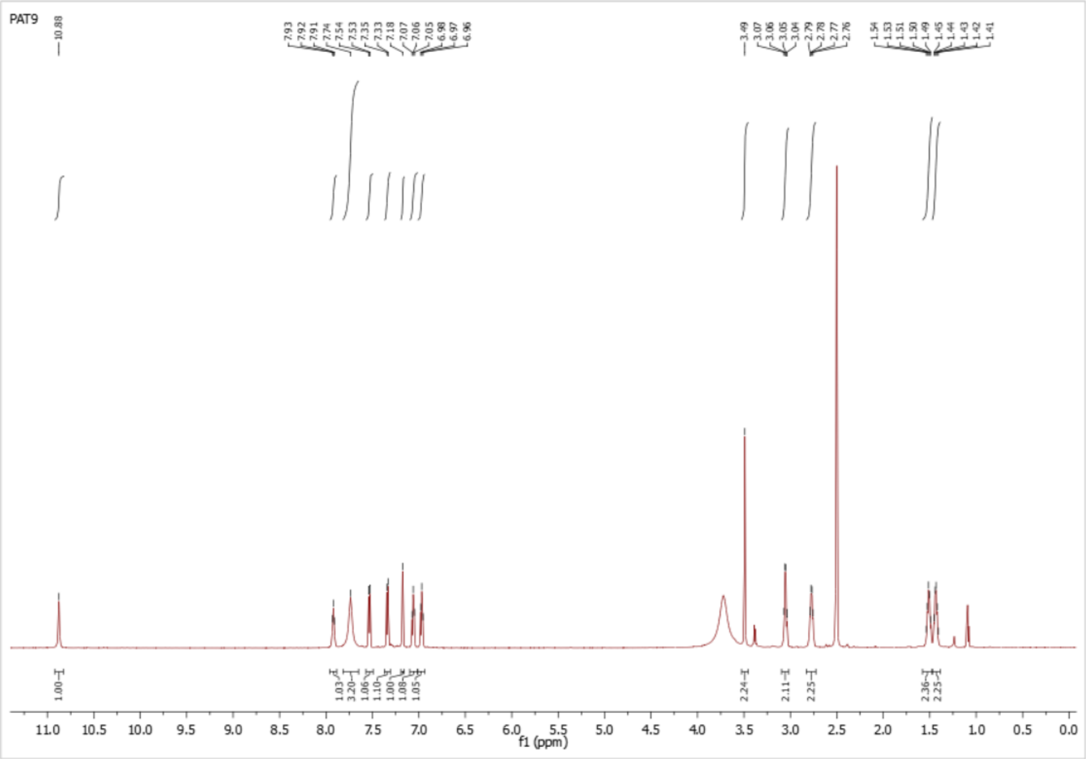

1.30 ^{13}C-NMR spectrum of PAT analog 3

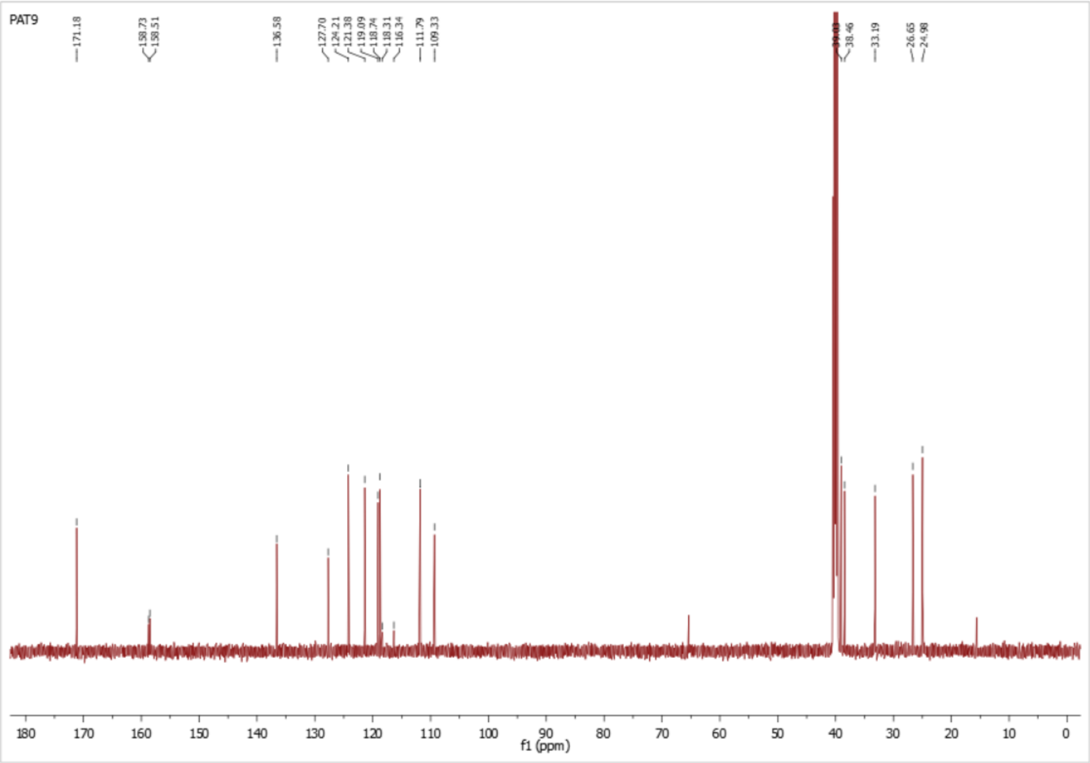

1.31 ^{1}H-NMR spectrum of compound 36

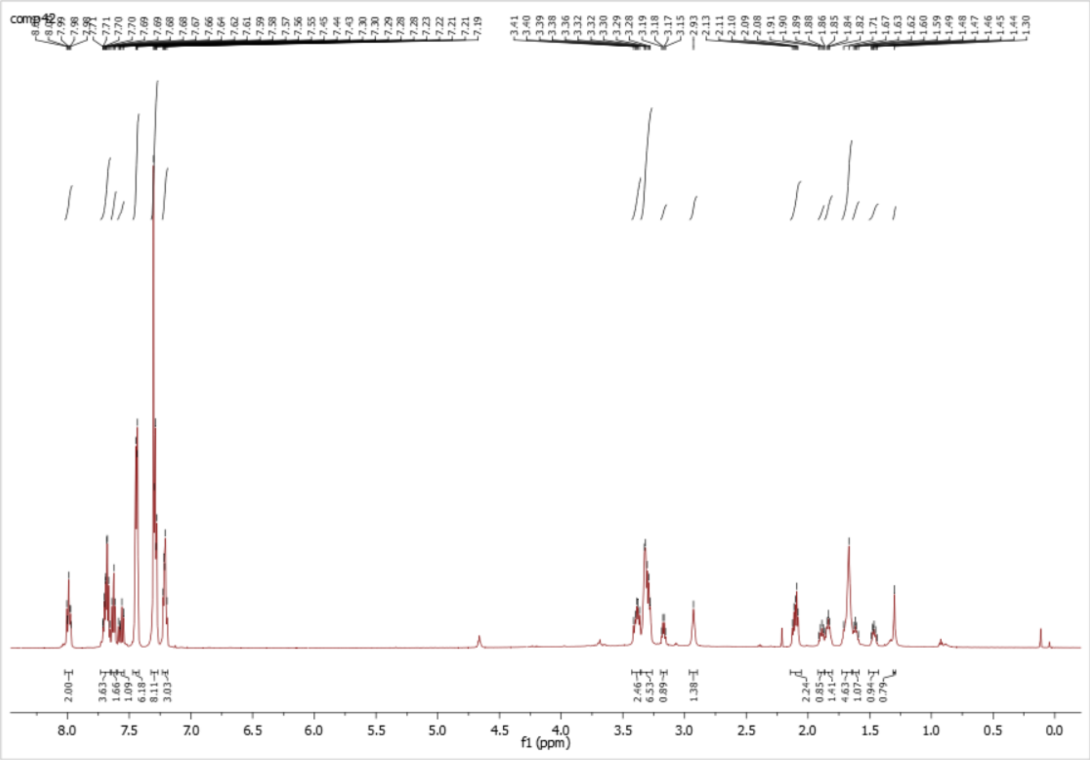

1.32 ^{1}H-NMR spectrum of compound 37

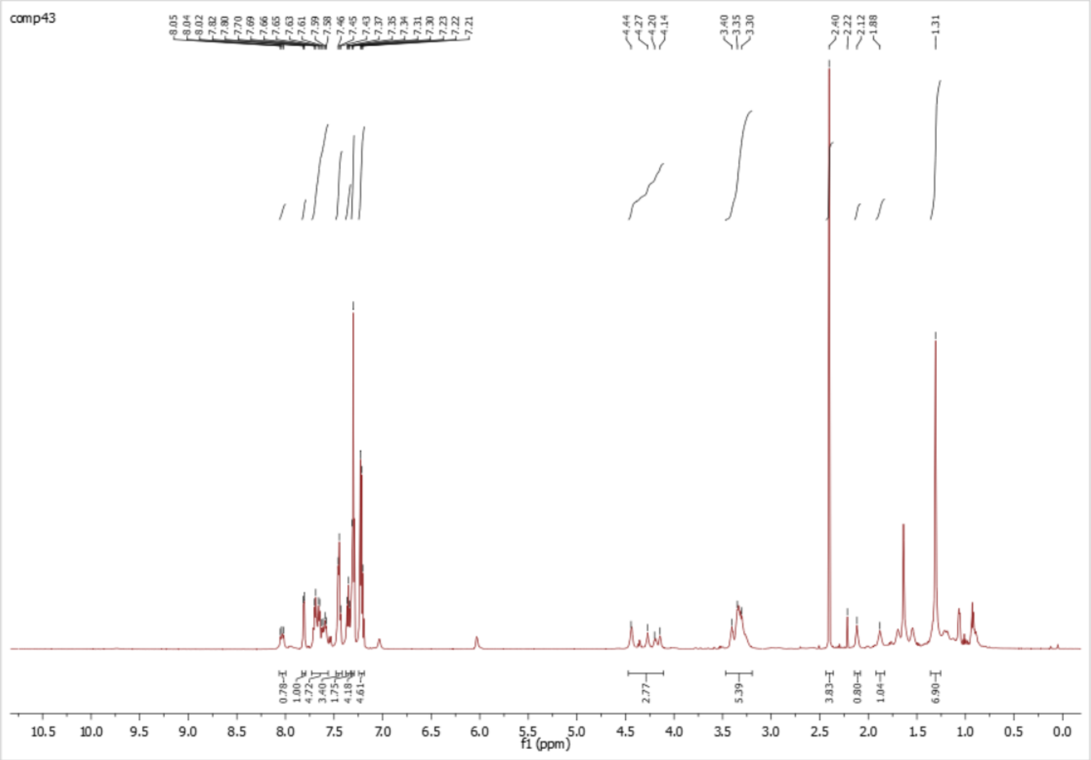

1.33 ^{13}C-NMR spectrum of compound 37

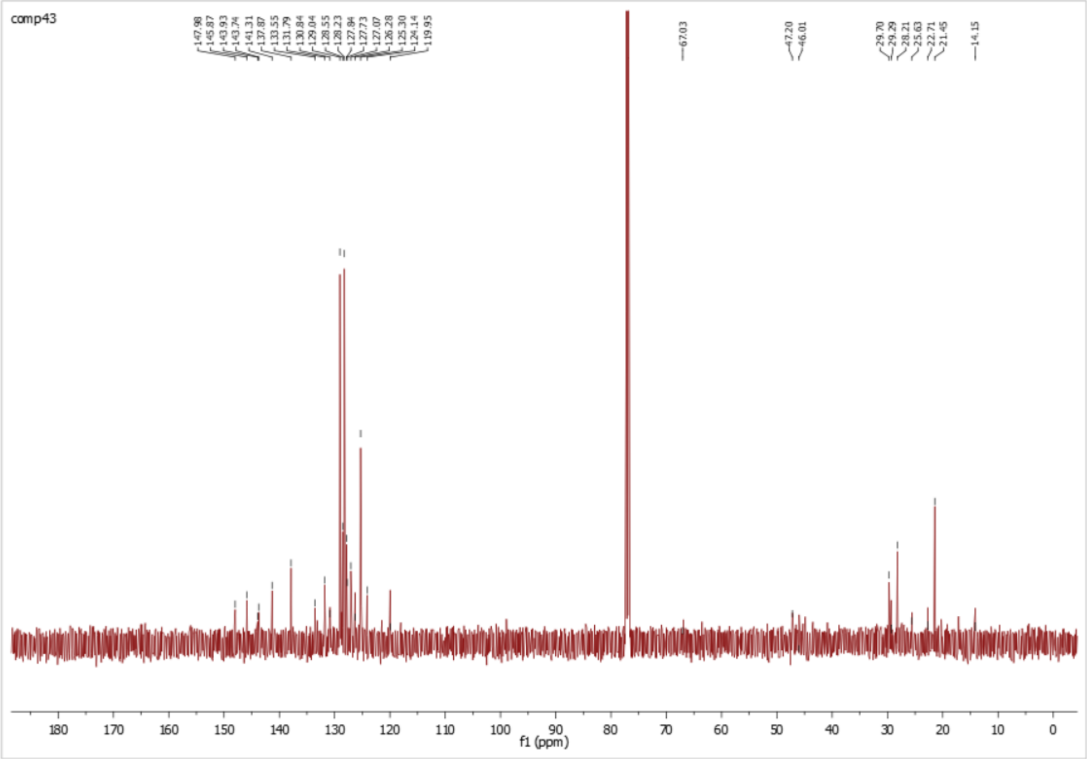

1.34 ^{1}H -NMR spectrum of compound 39

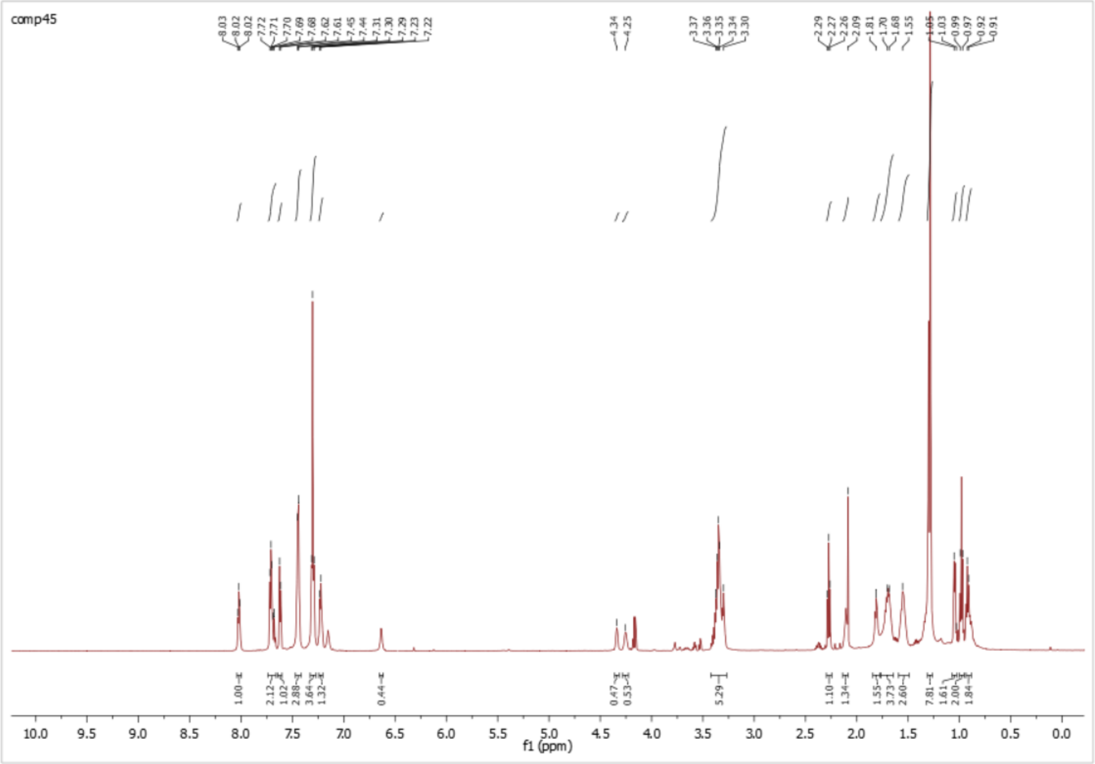

1.35 ^{13}C-NMR spectrum of compound 39

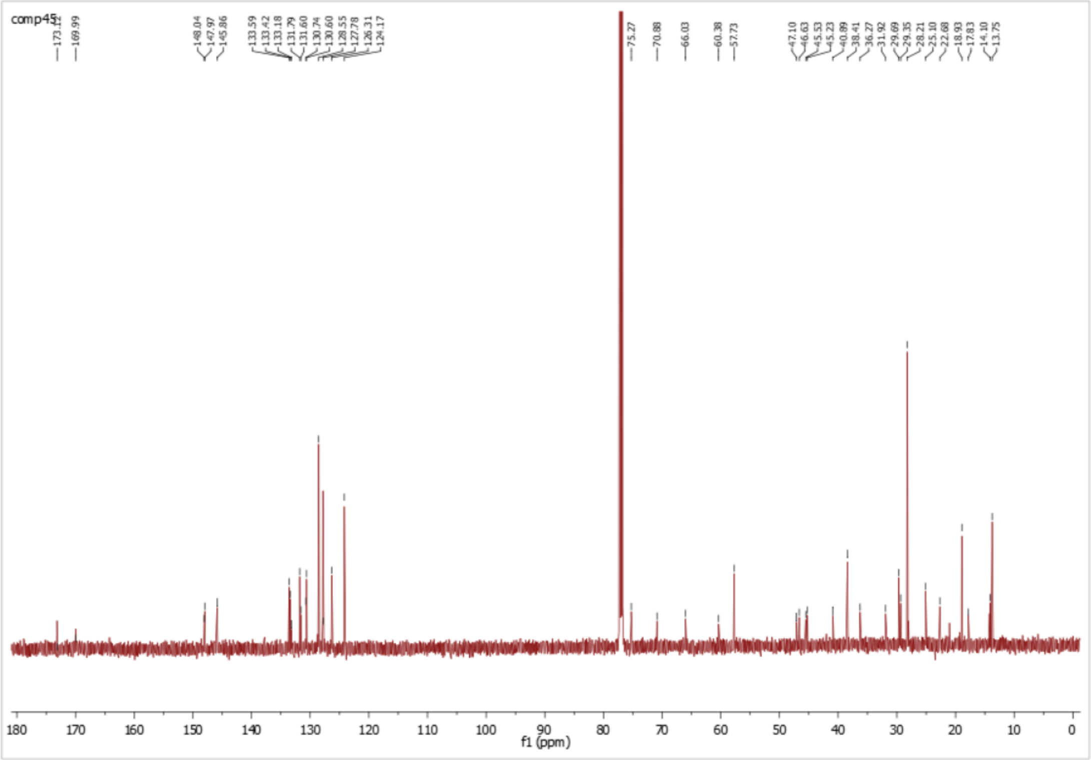

1.36 ^{1}H-NMR spectrum of compound 53

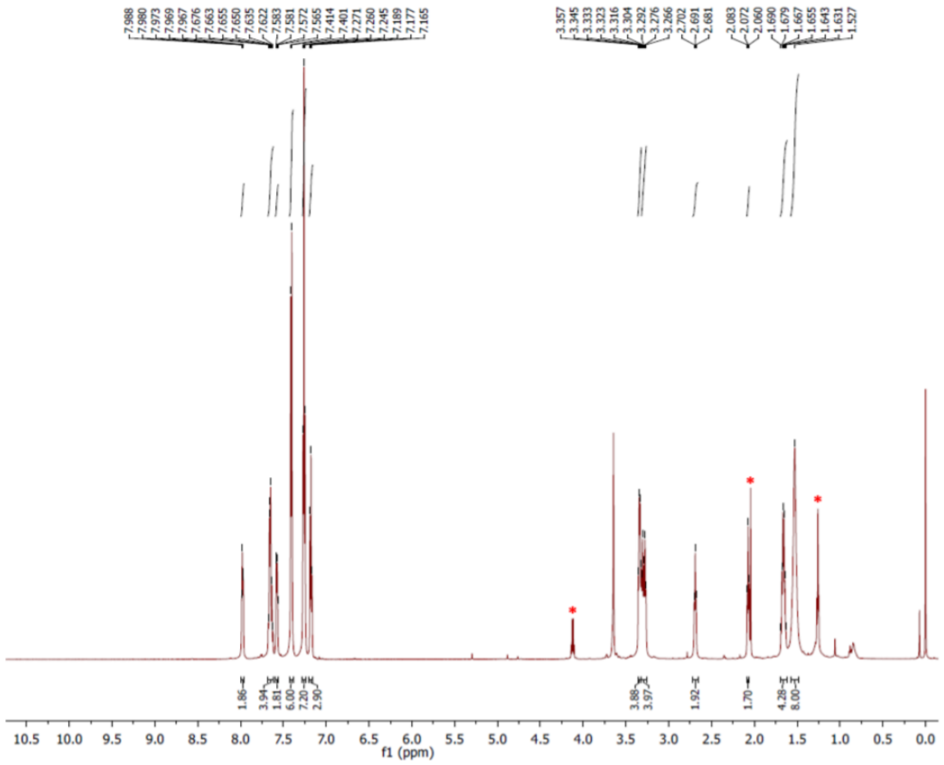

1.37 ^{13}C-NMR spectrum of compound 53

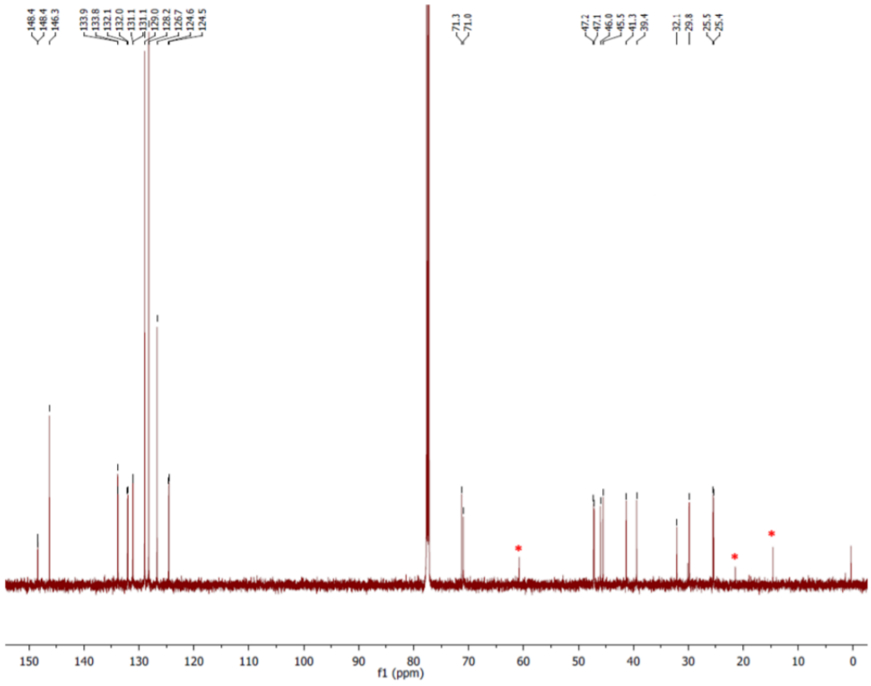

*Residual solvent

1.38 ^{1}H -NMR spectrum of compound 56

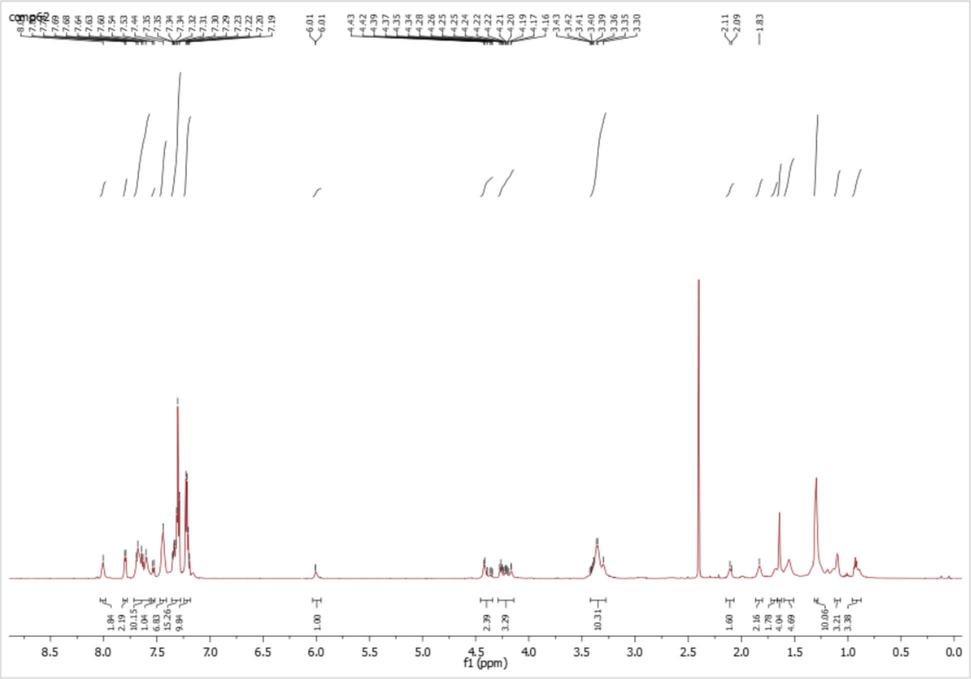

1.39 ^{13}C-NMR spectrum of compound 56

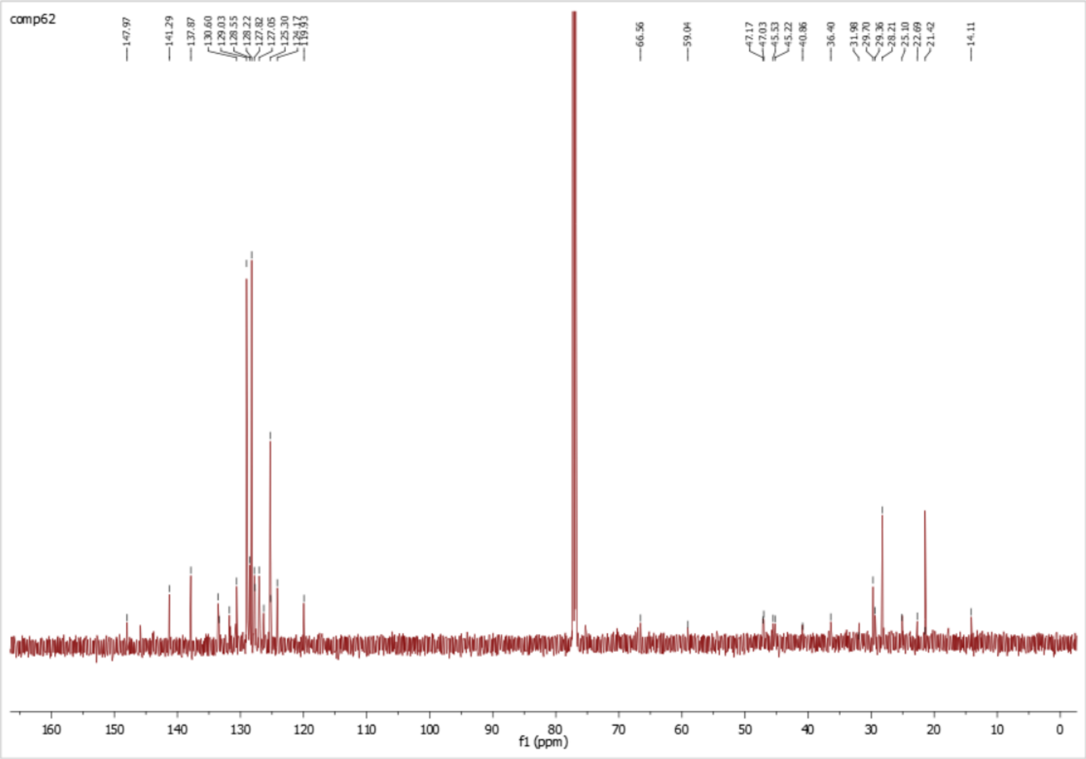

1.40 ^{1}H-NMR spectrum of compound 45

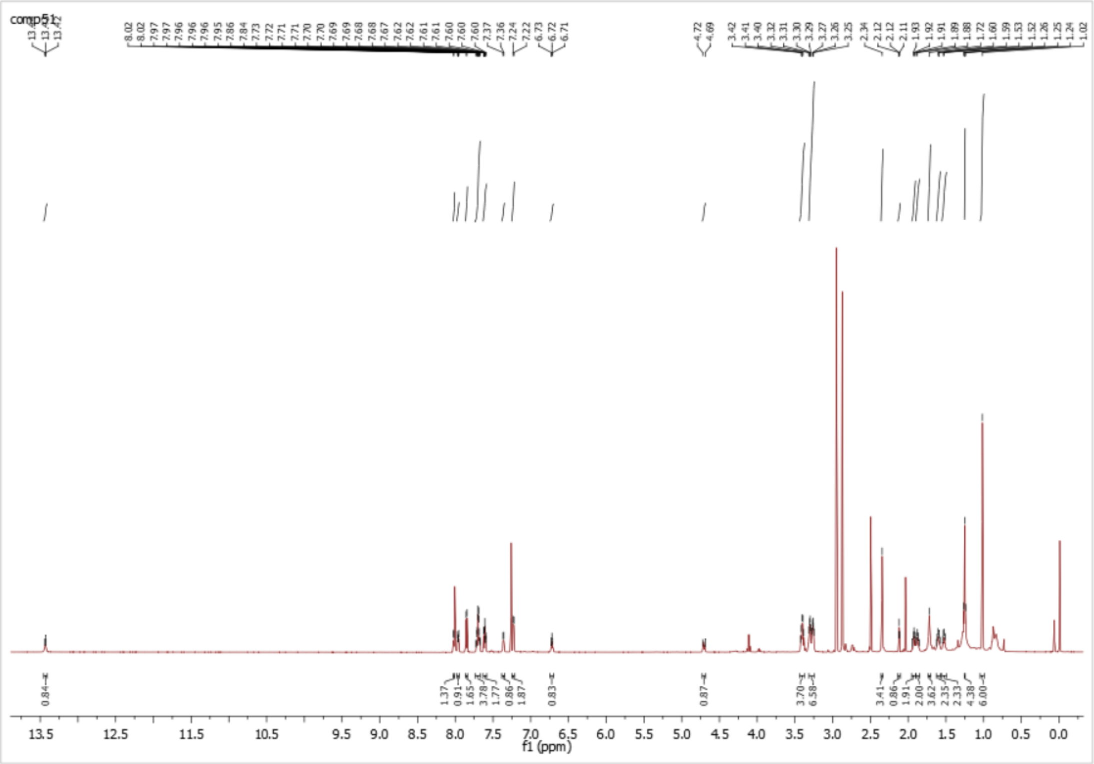

1.41 ^{13}C-NMR spectrum of compound 45

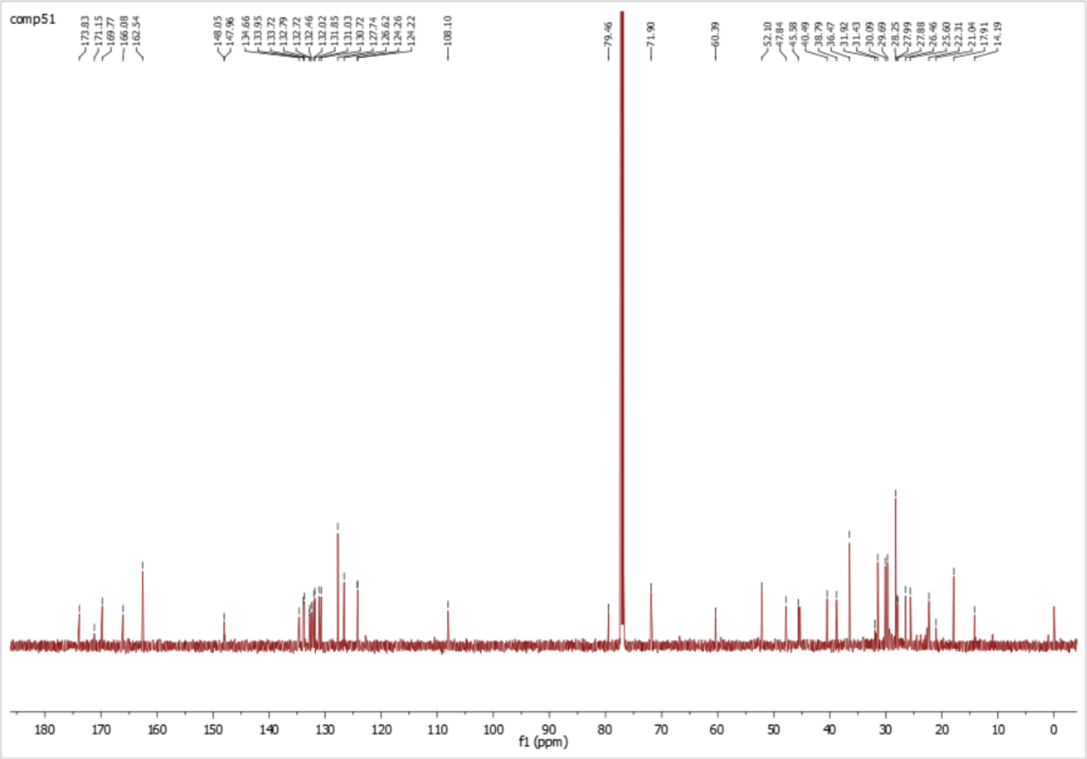

1.42 ^{1}H -NMR spectrum of compound 48

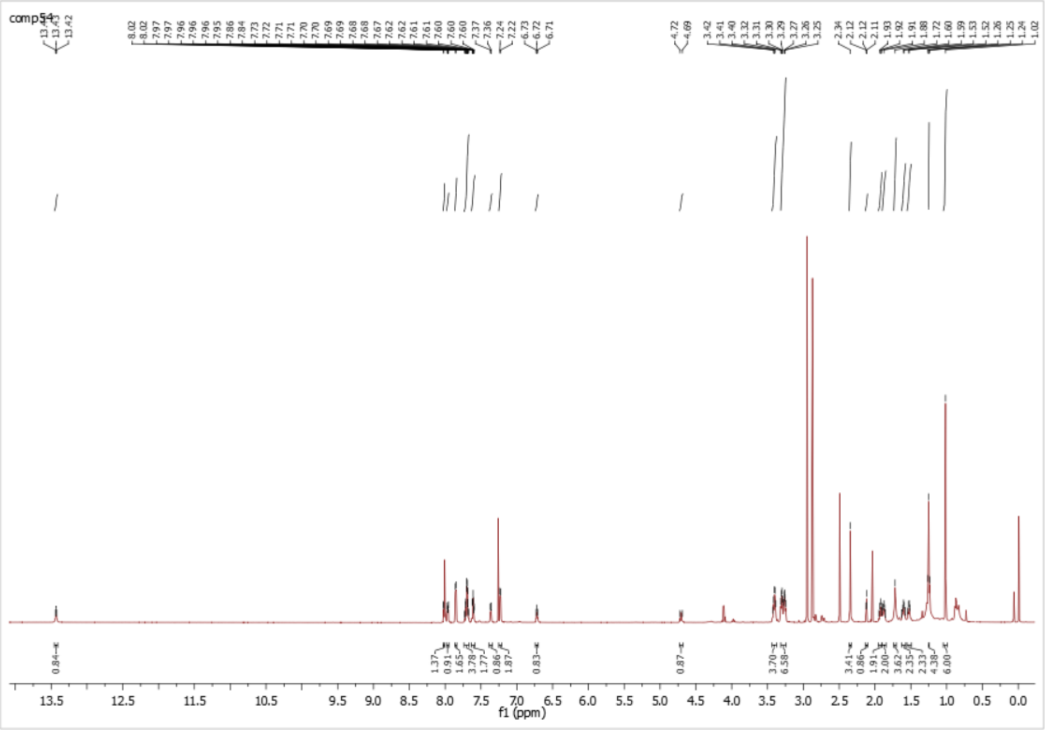

1.43 ^{13}C-NMR spectrum of compound 48

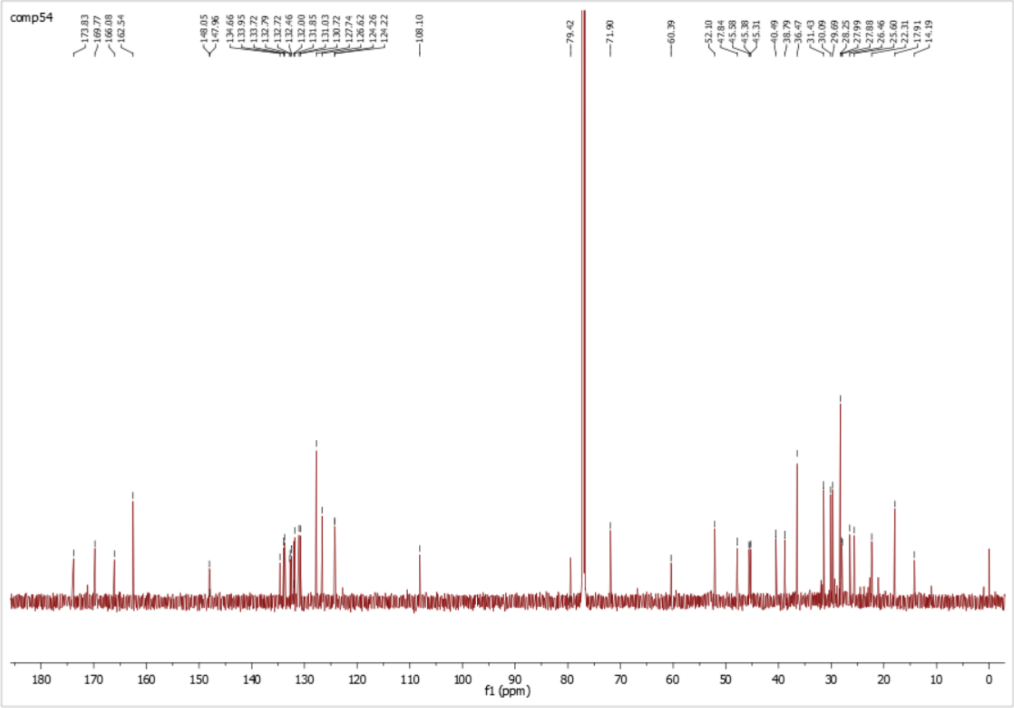

1.44 ^{1}H-NMR spectrum of PAT analog 9

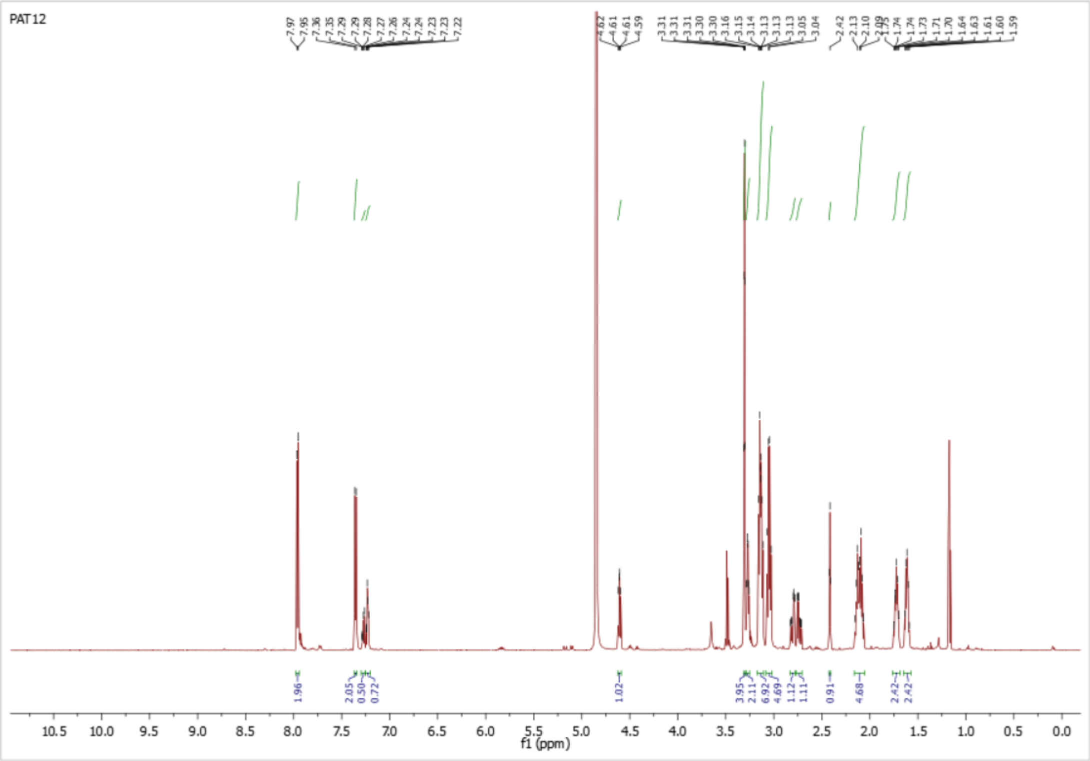

1.45 ^{13}C-NMR spectrum of PAT analog 9

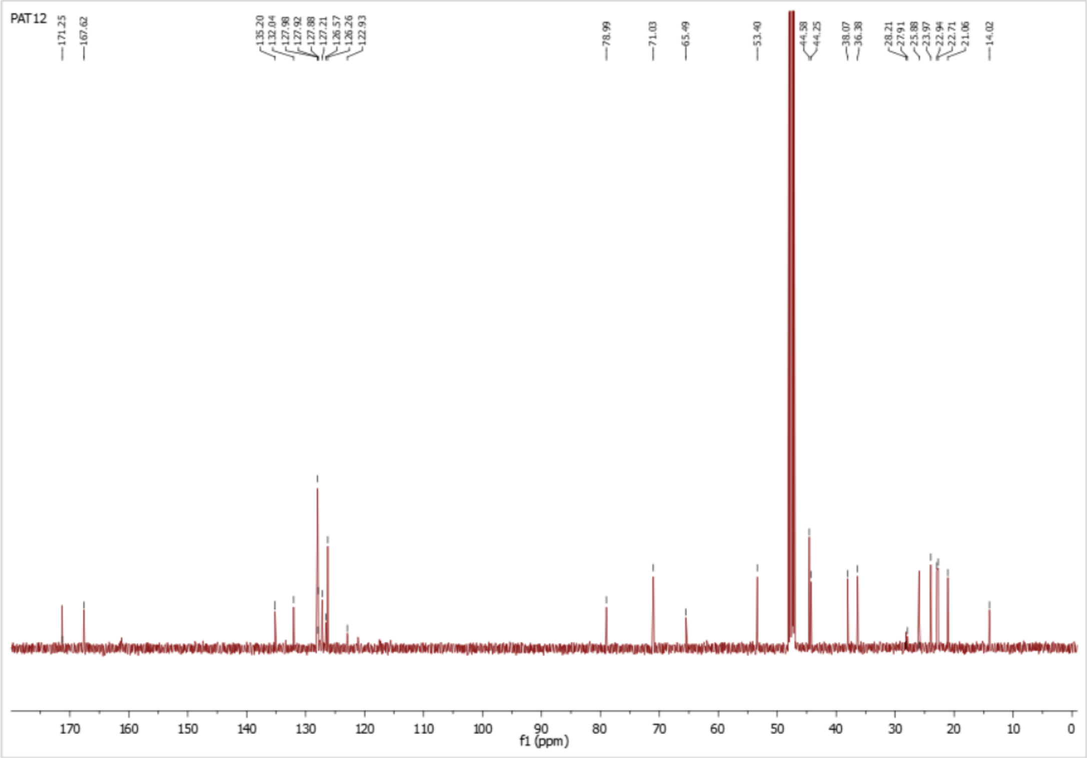

1.46 ^{1}H -NMR spectrum of compound 49

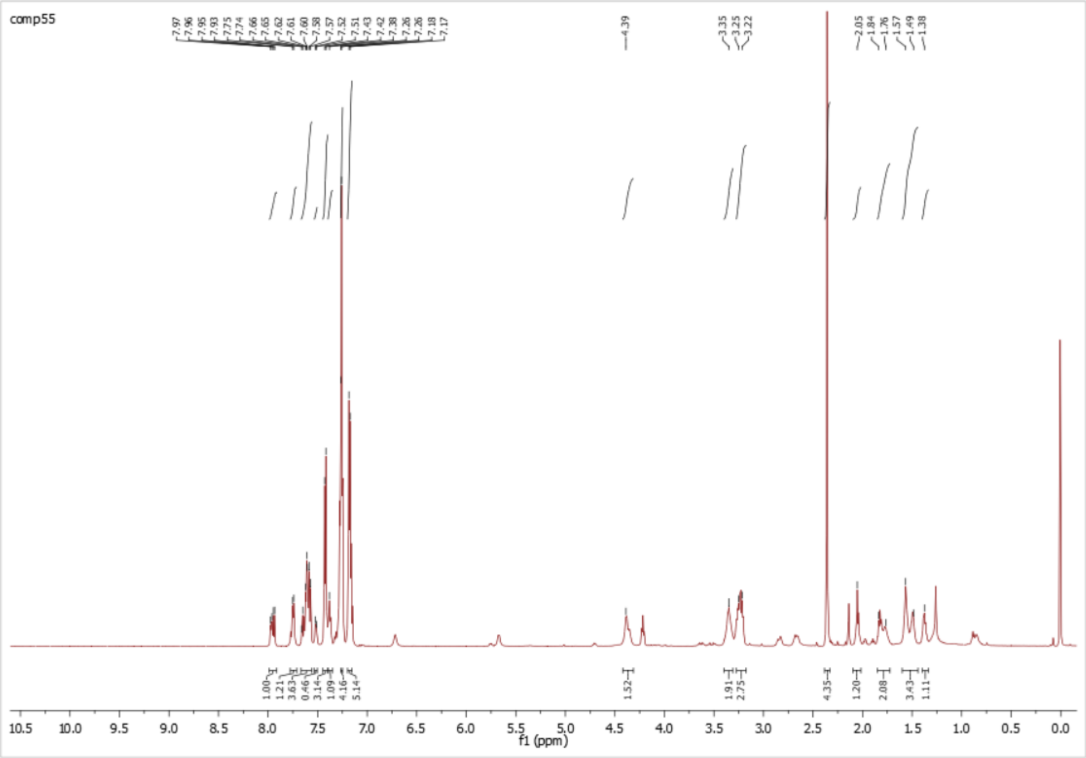

1.47 ^{13}C-NMR spectrum of compound 49

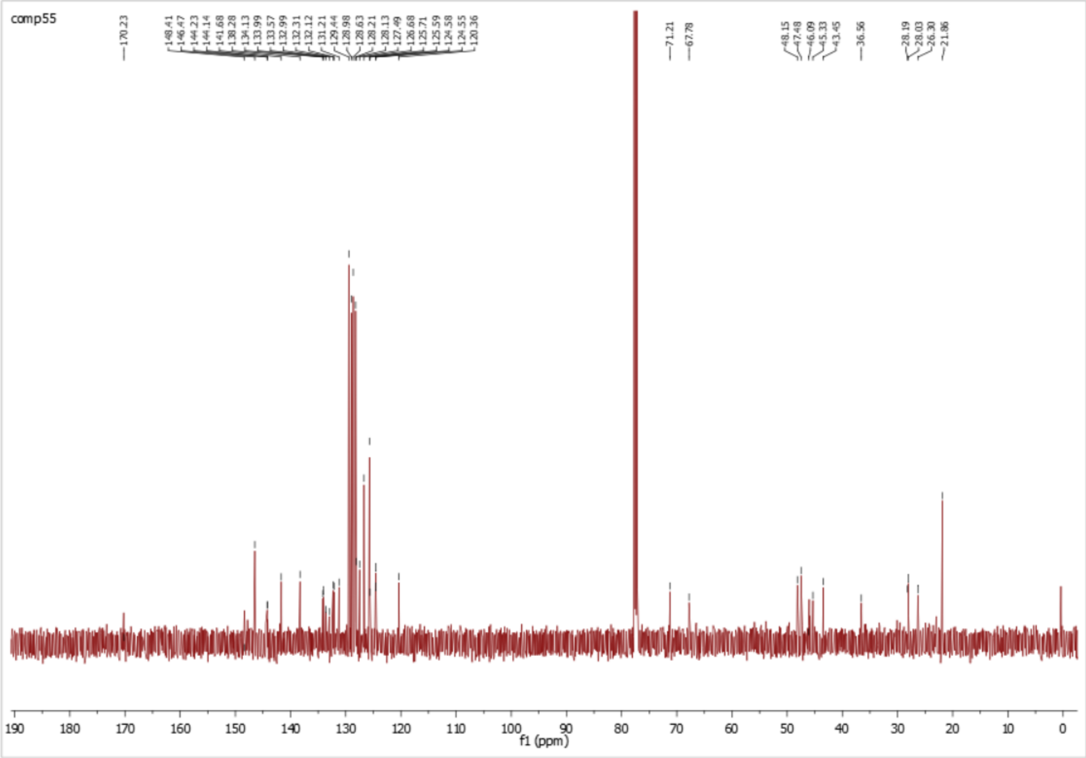

1.48 ^{1}H-NMR spectrum of compound 52

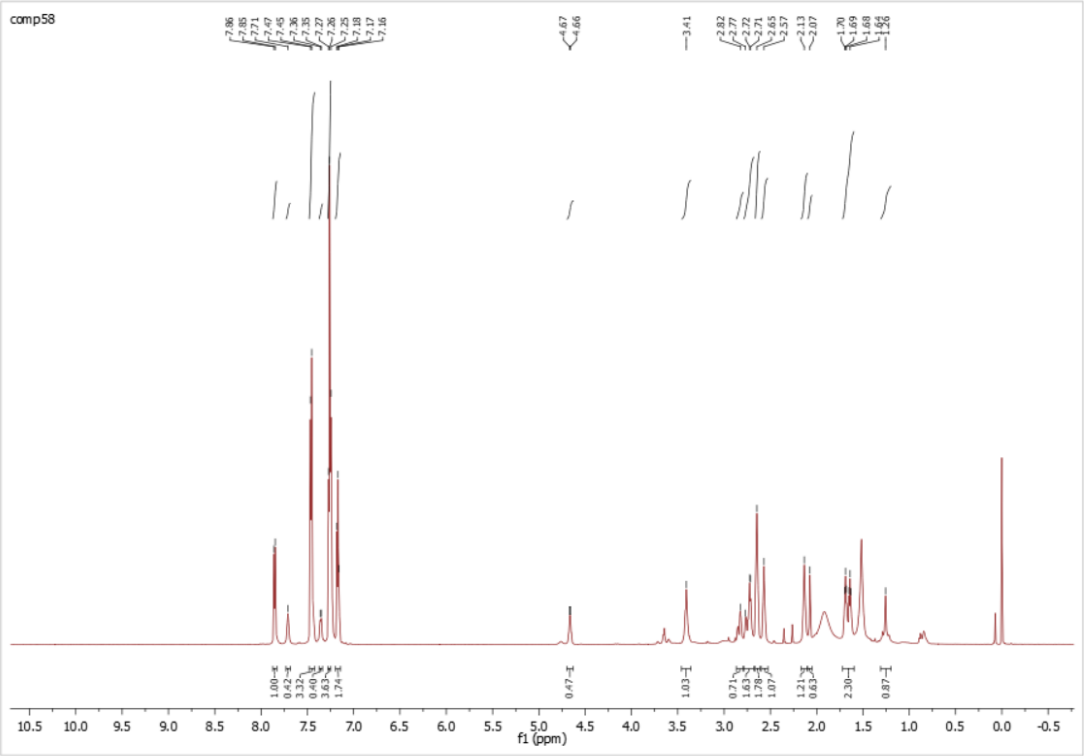

1.49 ^{13}C-NMR spectrum of compound 52

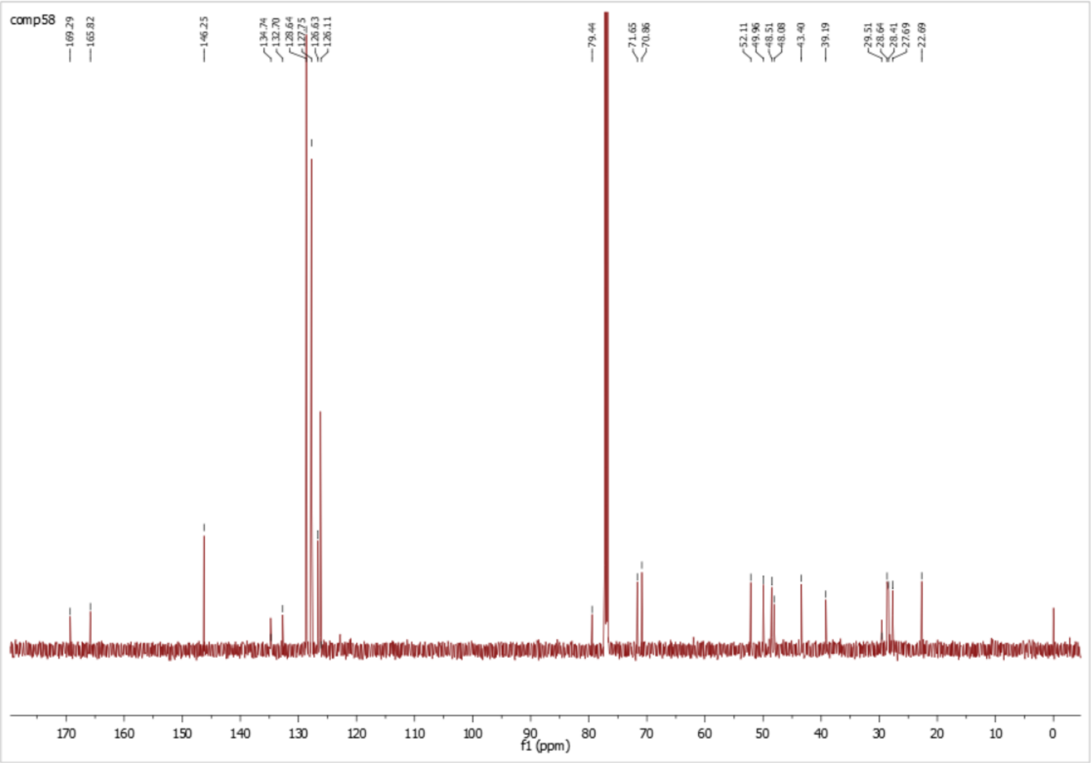

1.50 ^{1}H -NMR spectrum of PAT analog 10

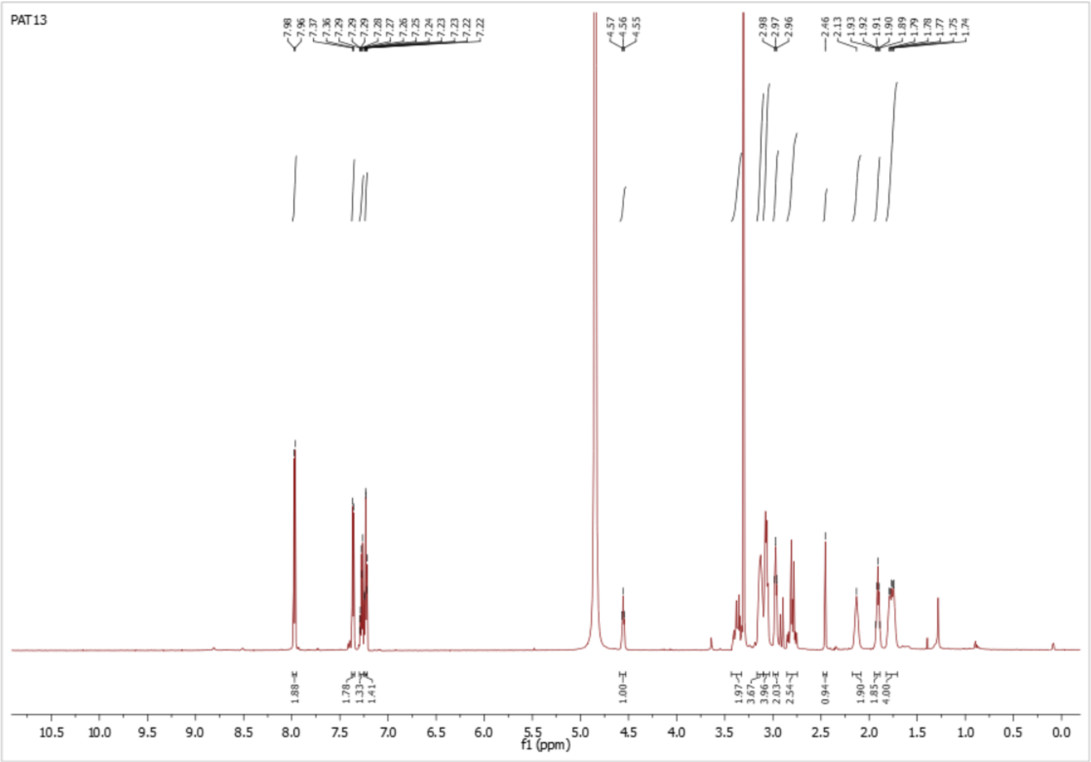

1.51 ^{13}C-NMR spectrum of PAT analog 10

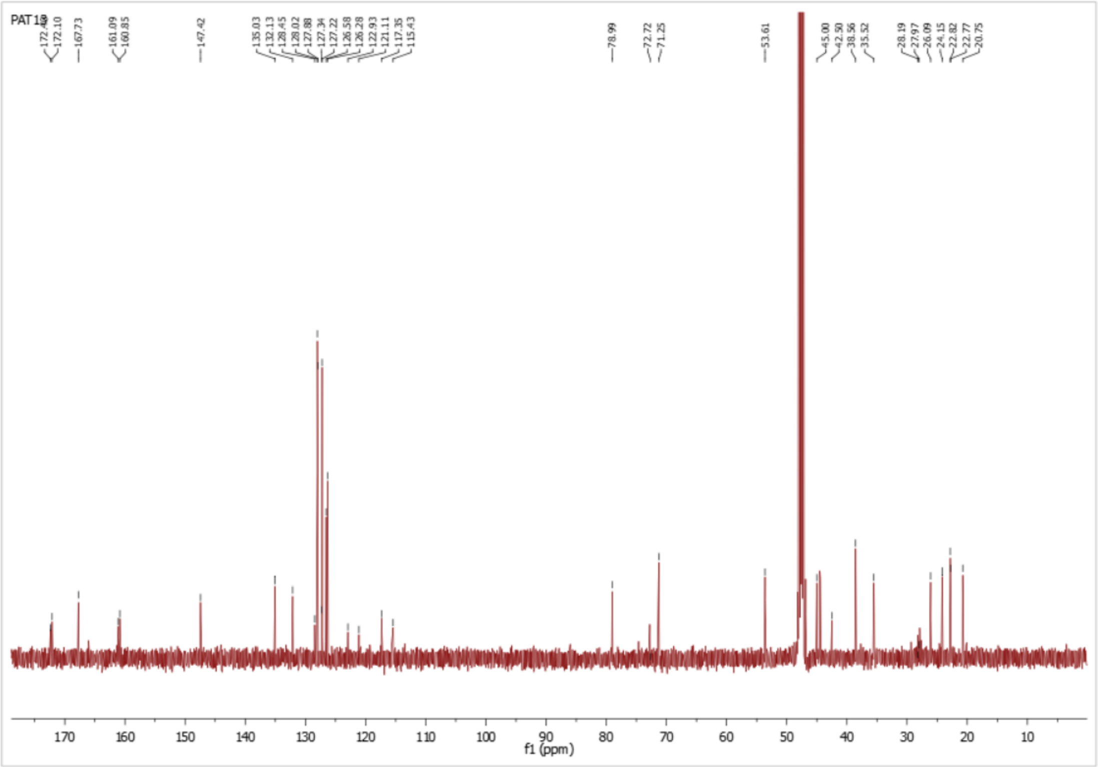

1.52 ^{1}H -NMR spectrum of compound 61

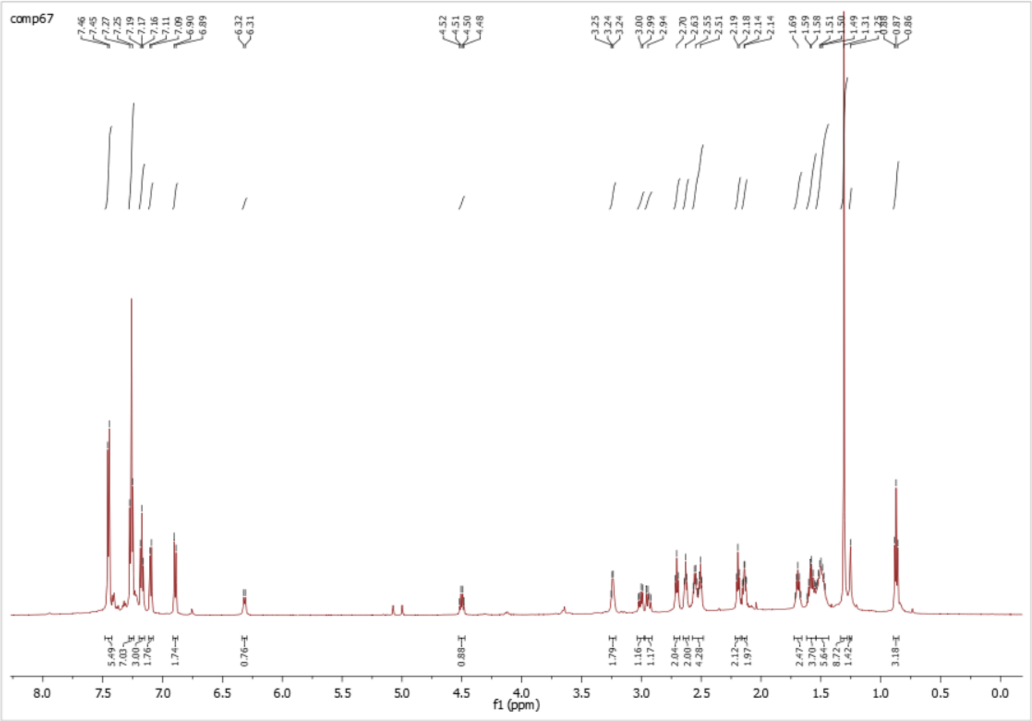

1.53 ^{13}C-NMR spectrum of compound 61

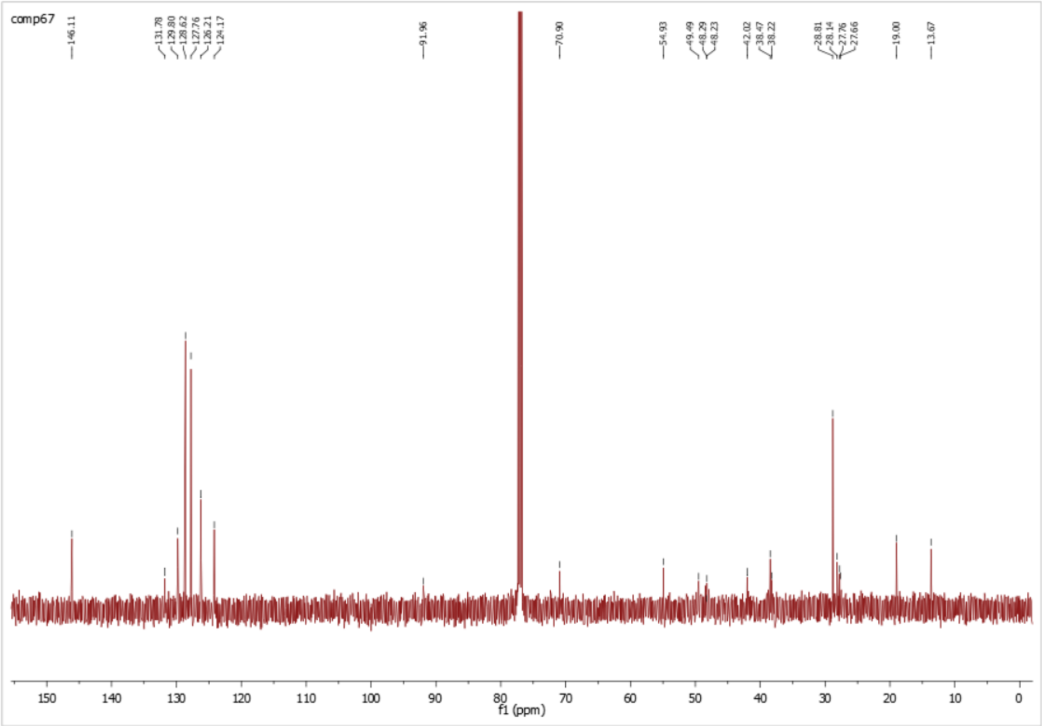

1.54 ^{1}H-NMR spectrum of compound 66

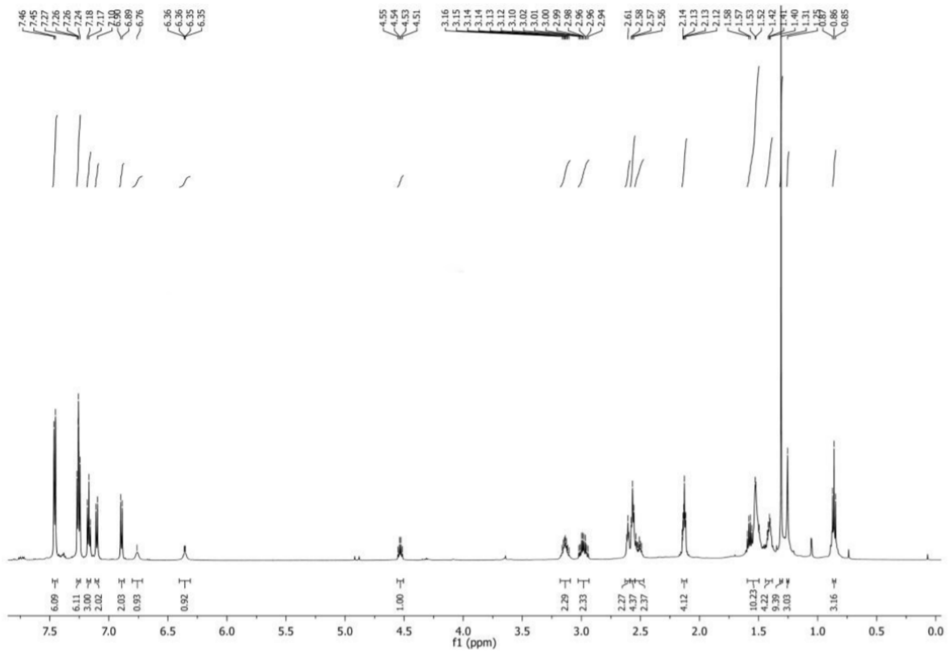

1.55 ^{13}C-NMR spectrum of compound 66

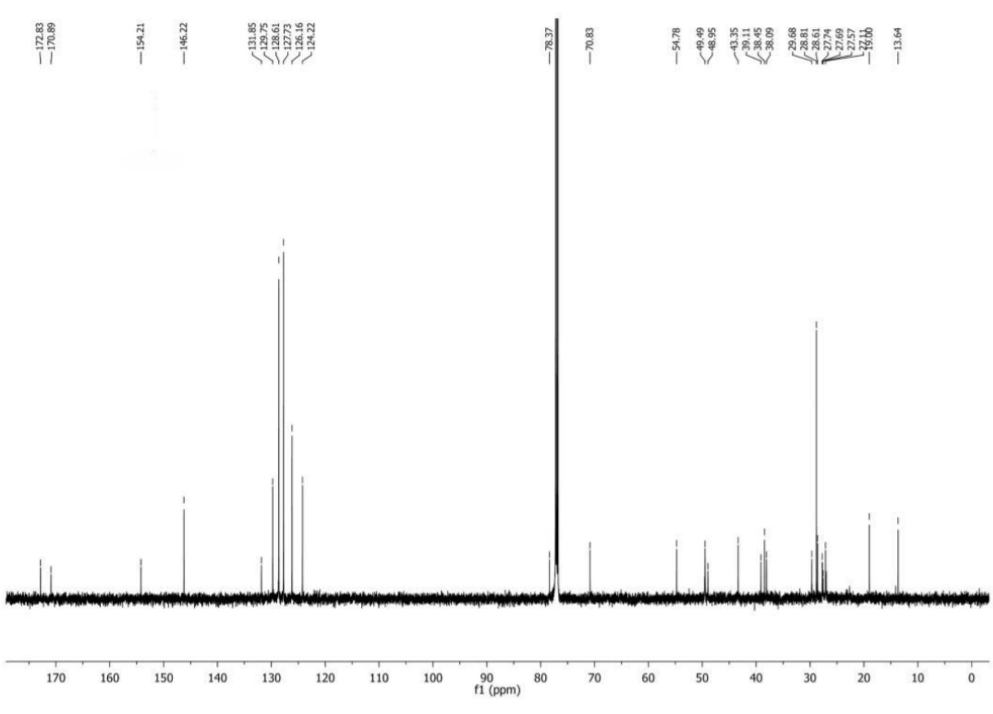

1.56 Copy of ^{1}H -NMR spectrum of compound 72

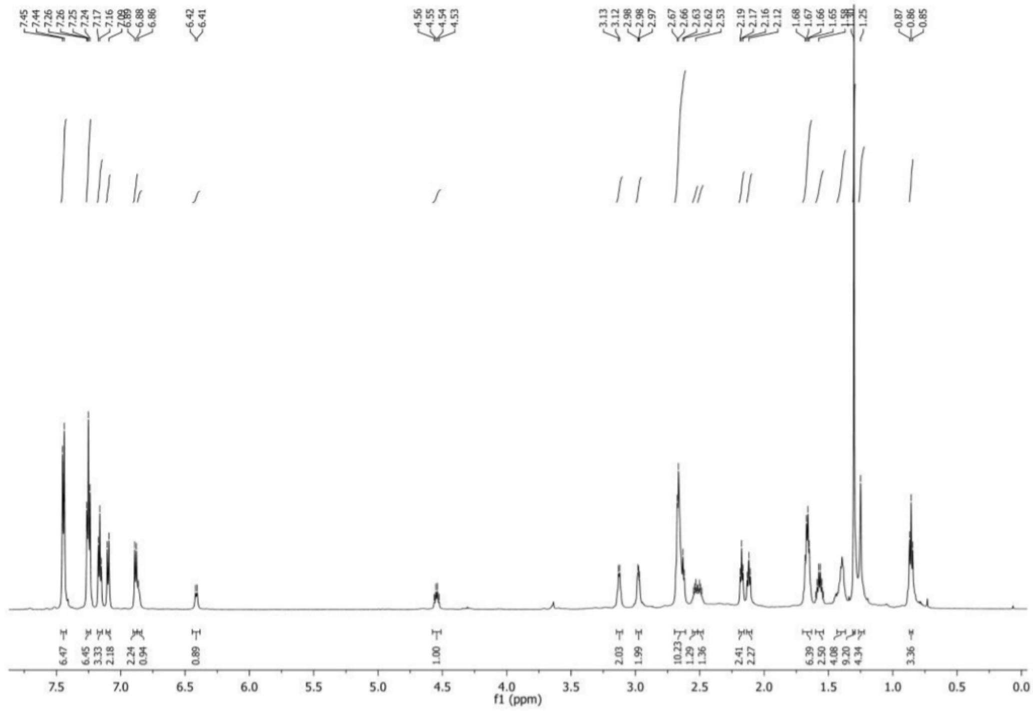

1.57 Copy of ^{13}C-NMR spectrum of compound 72

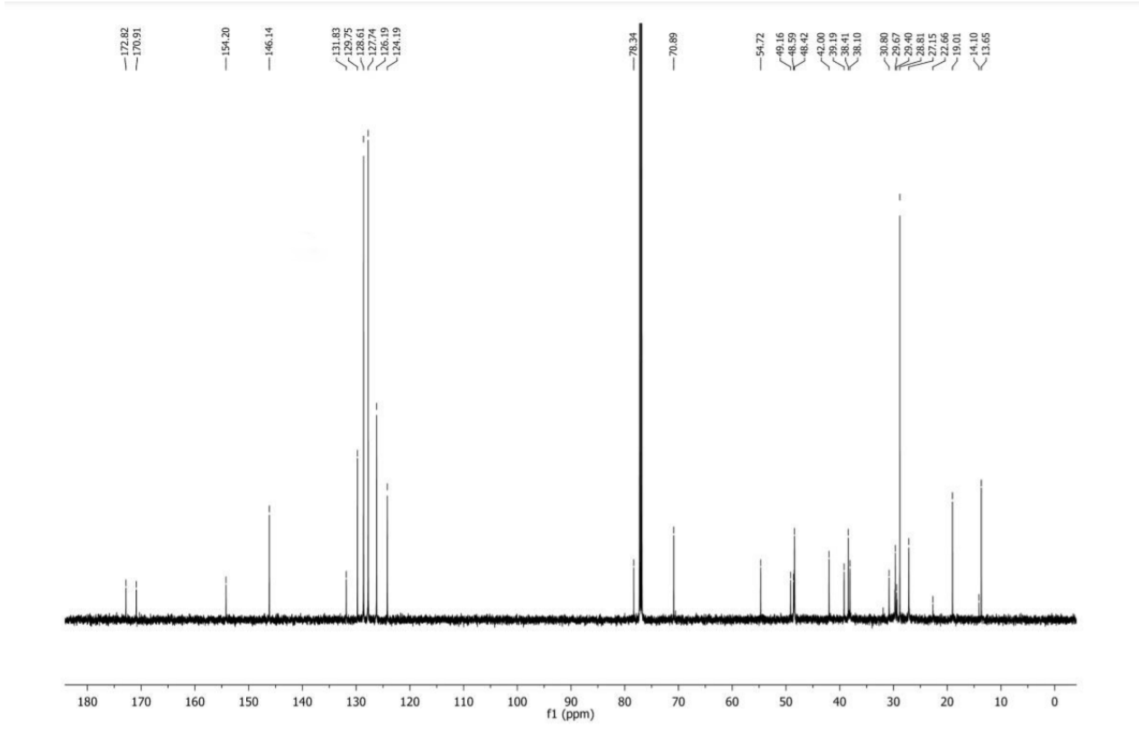

2. Diagrams of dose-dependent responses (DDR) of the tested compounds on MCF-7 and MDA-MB-231 breast cancer cells.

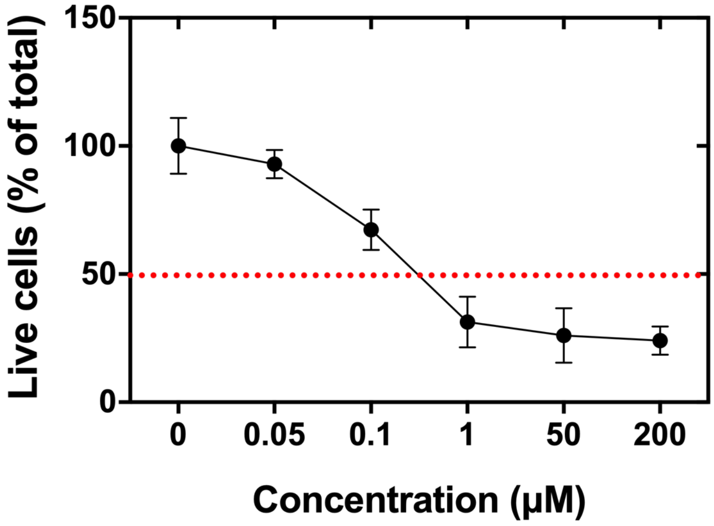

2.1 DDR diagram for compound Agel 416x5CF_3CO_2H for the MCF-7 cells

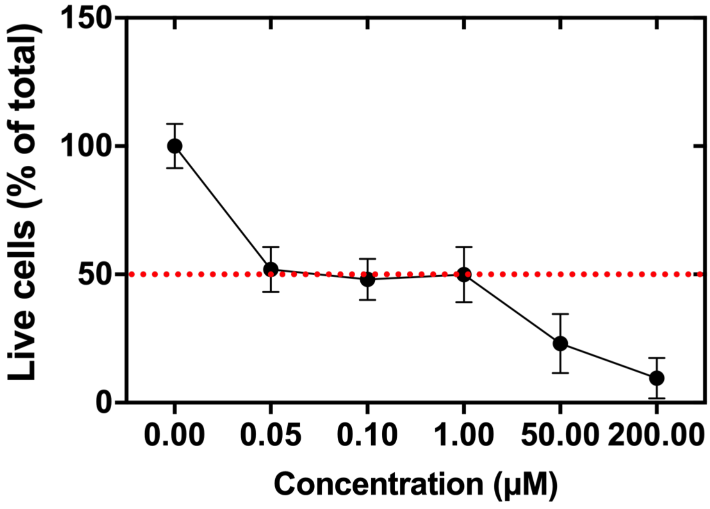

2.2 DDR diagram for compound Agel 416x5CF_3CO_2H for the MDA-MB-231 cells

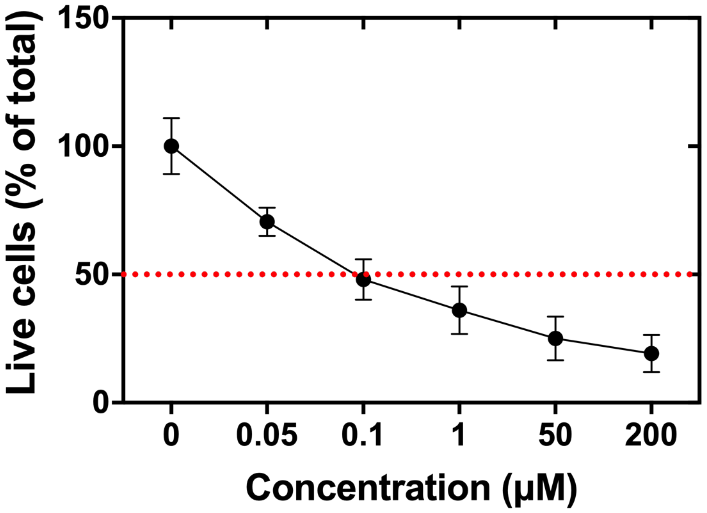

2.3 DDR diagram for compound HO-416bx5CF_3CO_2H for the MCF-7 cells

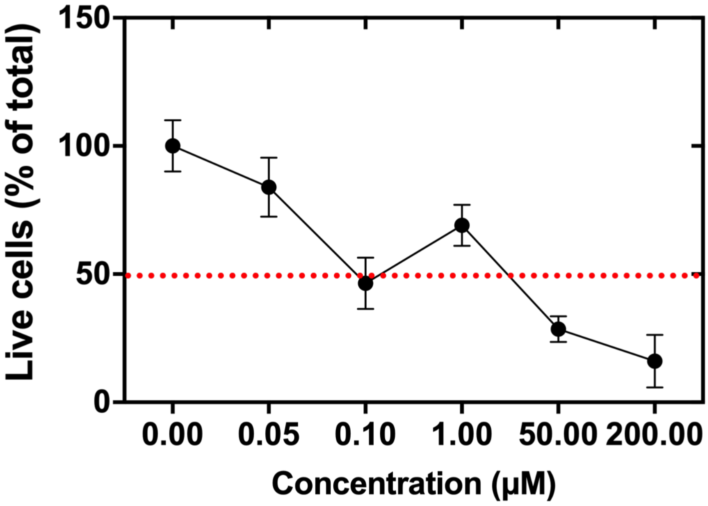

2.4 DDR diagram for compound HO-416bx5CF_3CO_2H for the MDA-MB-231 cells

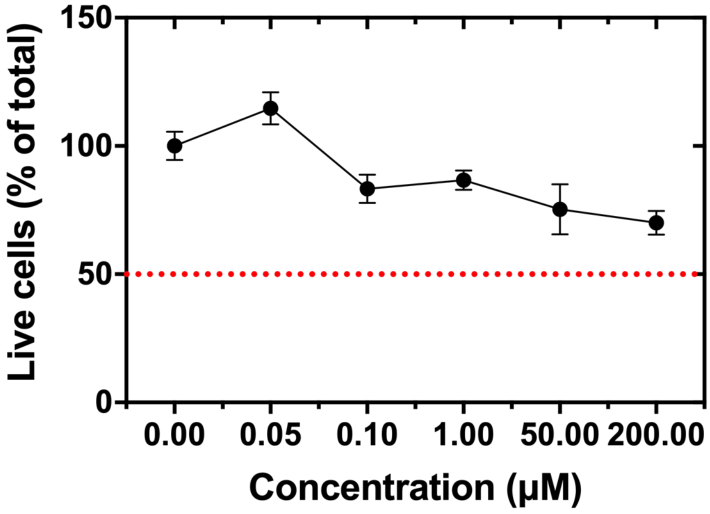

2.5 DDR diagram for compound **1**x4CF_3CO_2H for the MCF-7 cells

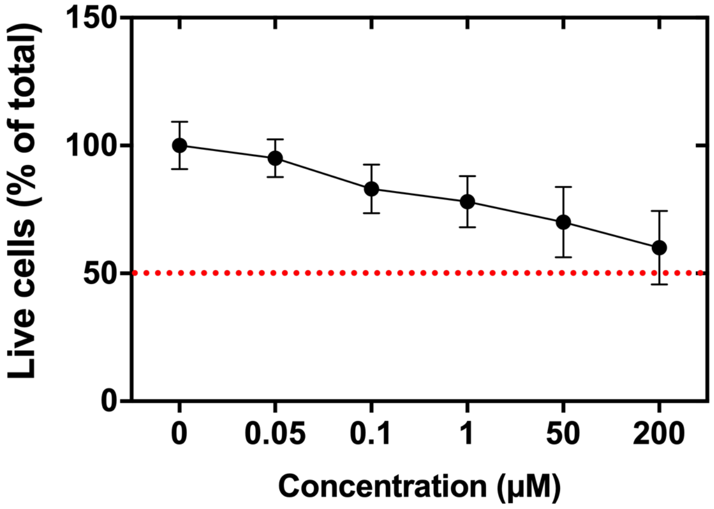

2.6 DDR diagram for compound **1**x4CF_3CO_2H for the MDA-MB-231 cells

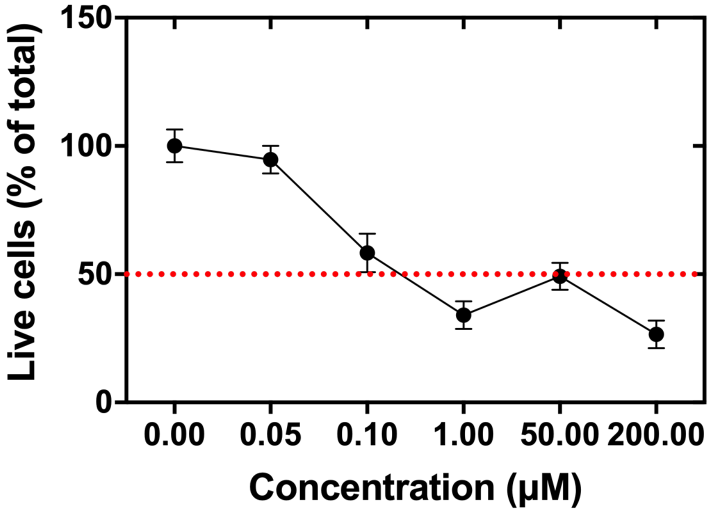

2.7 DDR diagram for compound **2**x3CF_3CO_2H for the MCF-7 cells

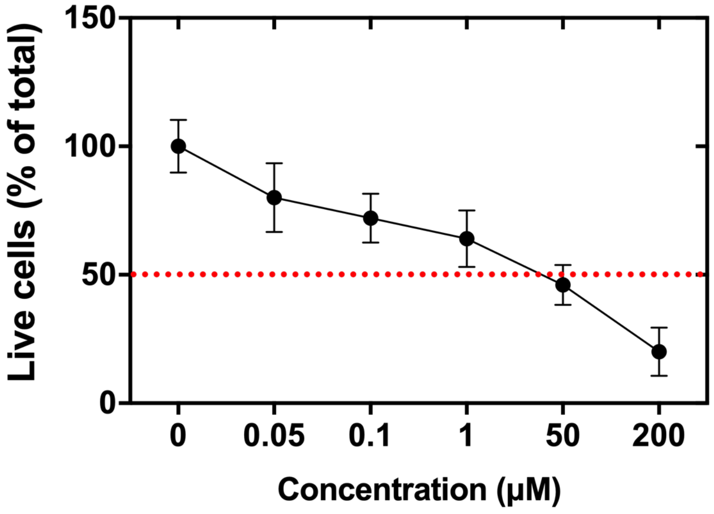

2.8 DDR diagram for compound **2**x3CF_3CO_2H for the MDA-MB-231 cells

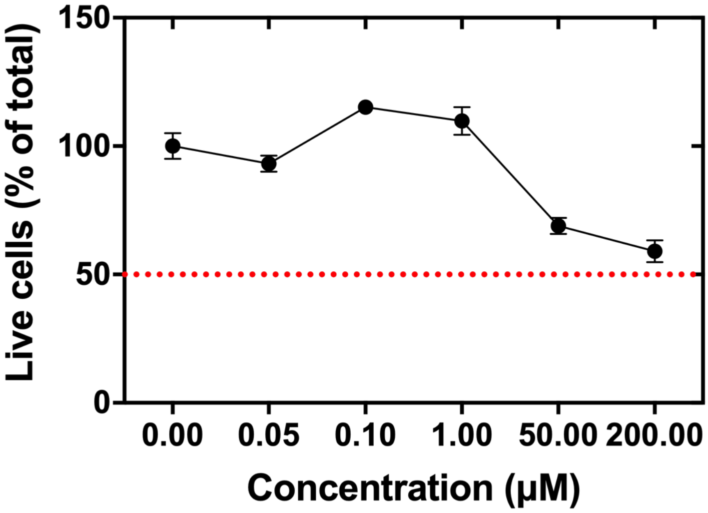

2.9 DDR diagram for compound **3**x2CF_3CO_2H for the MCF-7 cells

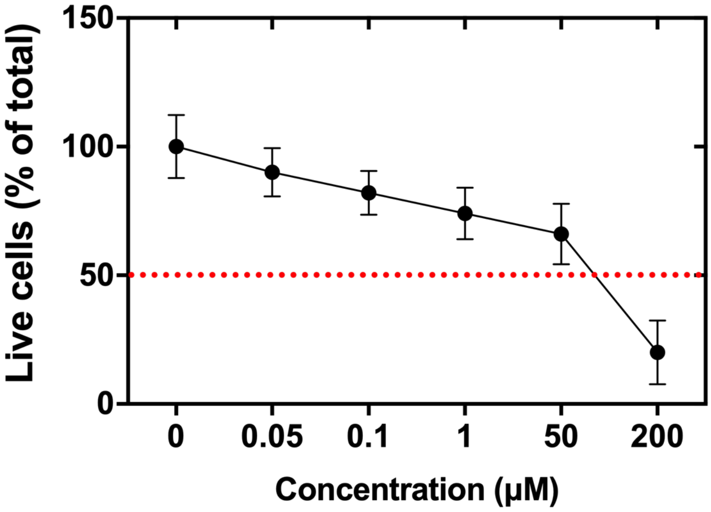

2.10 DDR diagram for compound **3**x2CF_3CO_2H for the MDA-MB-231 cells

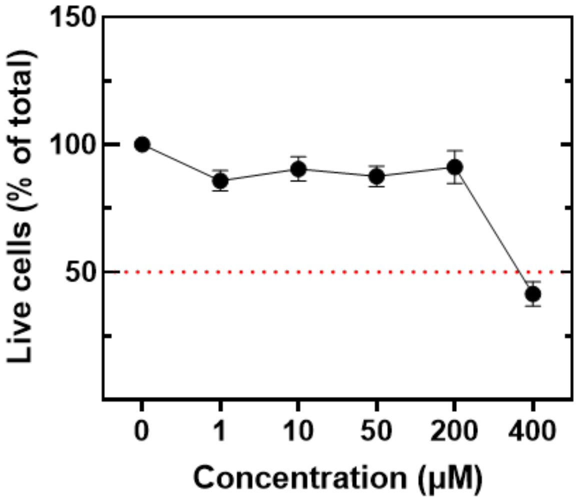

2.11 DDR diagram for acid **21** for the MCF-7 cells

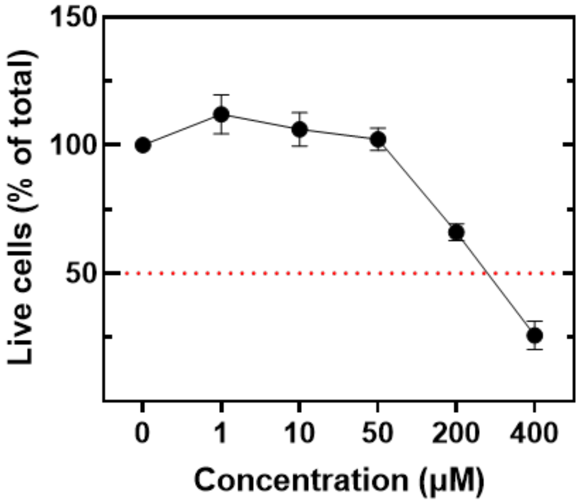

2.12 DDR diagram for acid **21** for the MDA-MB-231 cells

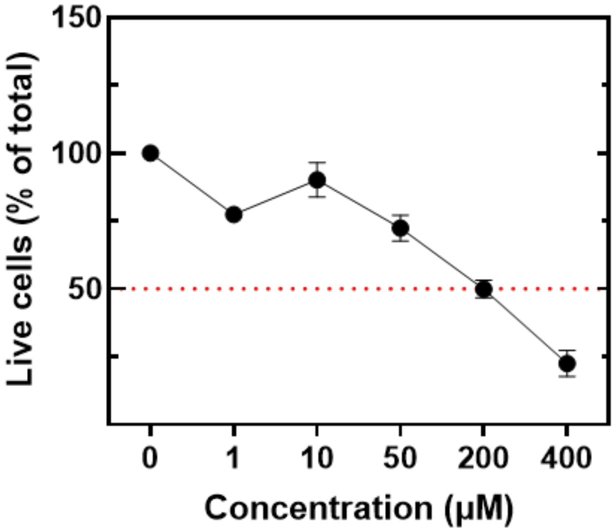

2.13 DDR diagram for the equimolar mixture **21**+Spd for the MCF-7 cells

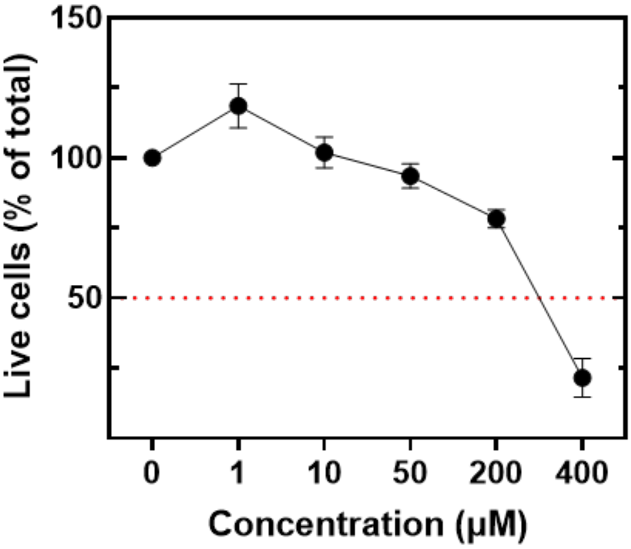

2.14 DDR diagram for the equimolar mixture **21**+Spd for the MDA-MB-231 cells

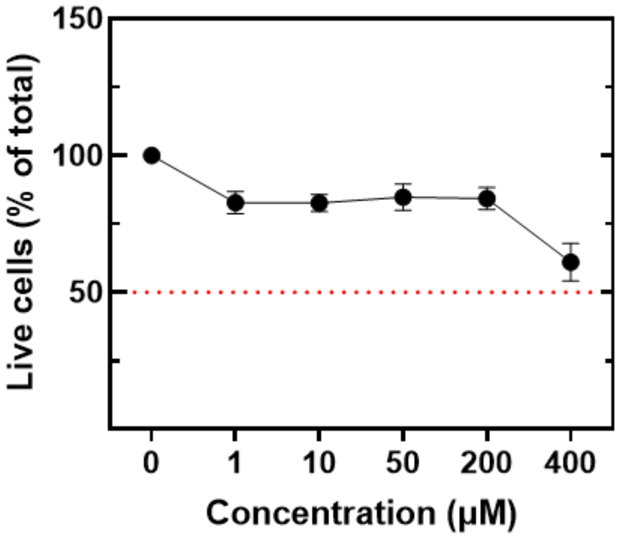

2.15 DDR diagram for compound **4**x3CF_3CO_2H for the MCF-7 cells

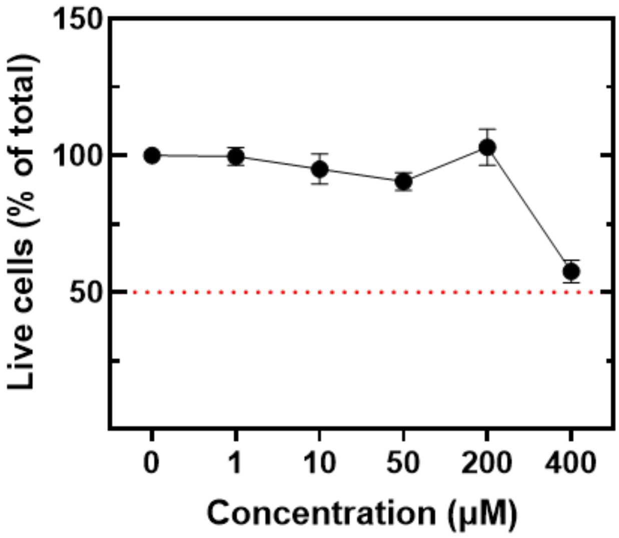

2.16 DDR diagram for compound **4**x3CF_3CO_2H for the MDA-MB-231 cells

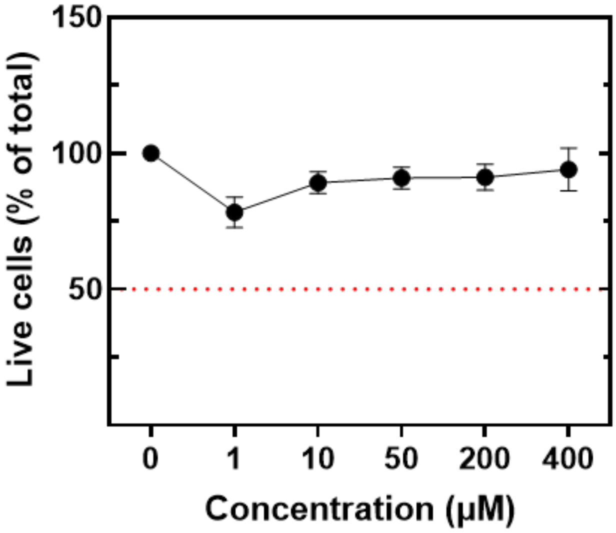

2.17 DDR diagram for compound **5**x3CF_3CO_2H for the MCF-7 cells

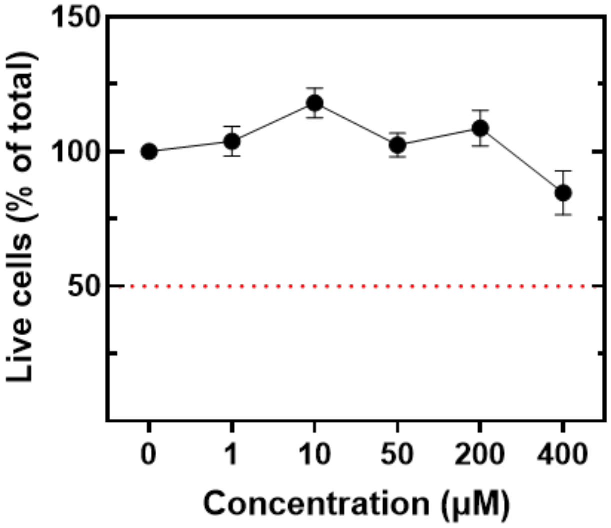

2.18 DDR diagram for compound **5**x3CF_3CO_2H for the MDA-MB-231 cells

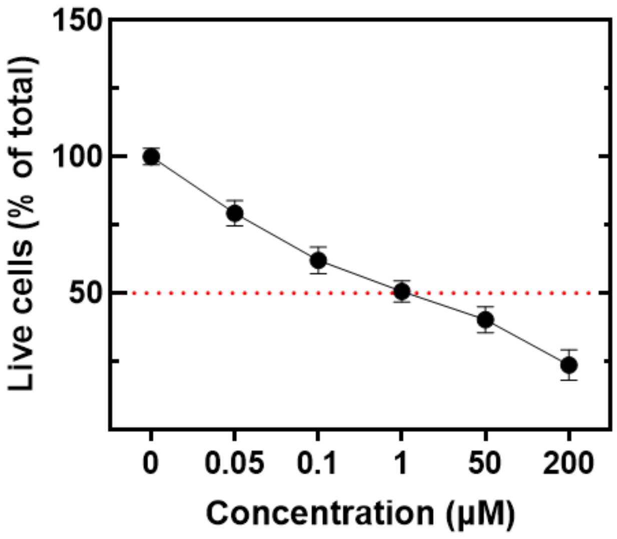

2.19 DDR diagram for compound **9**x3CF_3CO_2H for the MCF-7 cells

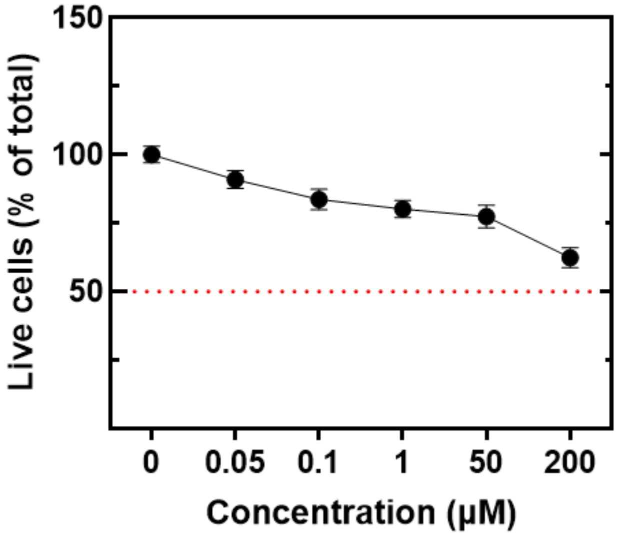

2.20 DDR diagram for compound **9**x3CF_3CO_2H for the MDA-MB-231 cells

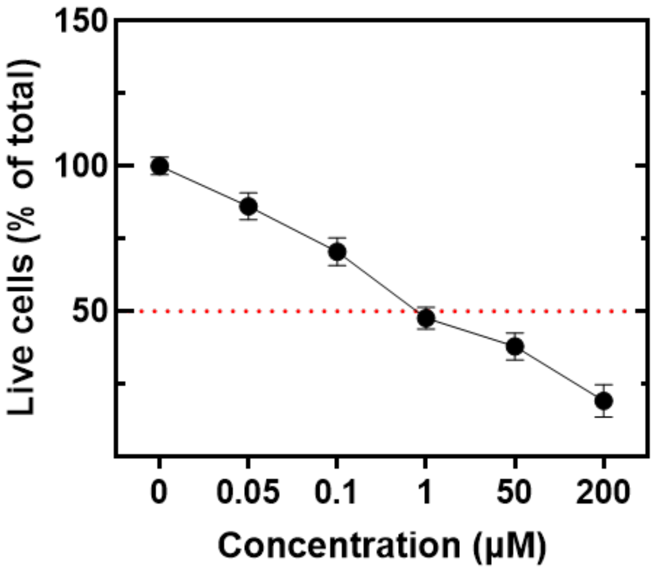

2.21 DDR diagram for compound **10**x3CF_3CO_2H for the MCF-7 cells

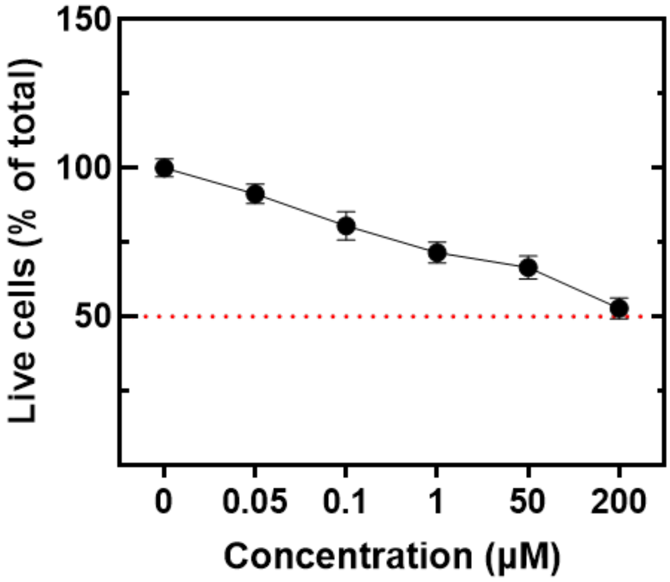

2.22 DDR diagram for compound **10**x3CF_3CO_2H for the MDA-MB-231 cells

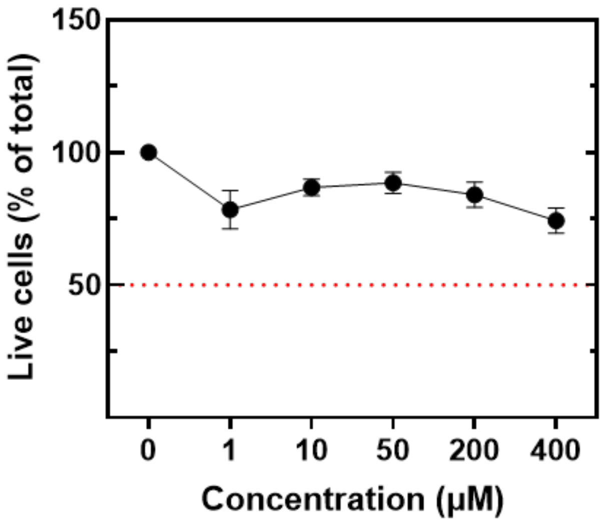

2.23 DDR diagram for compound **6**x3CF_3CO_2H for the MCF-7 cells

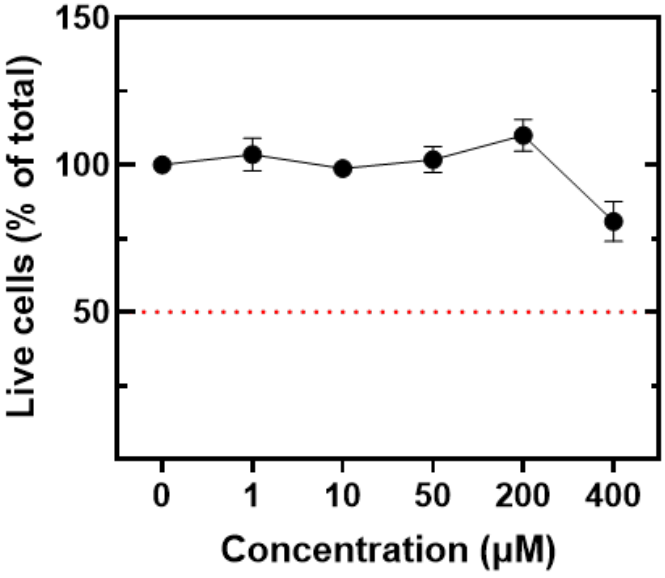

2.24 DDR diagram for compound **6**x3CF_3CO_2H for the MDA-MB-231 cells

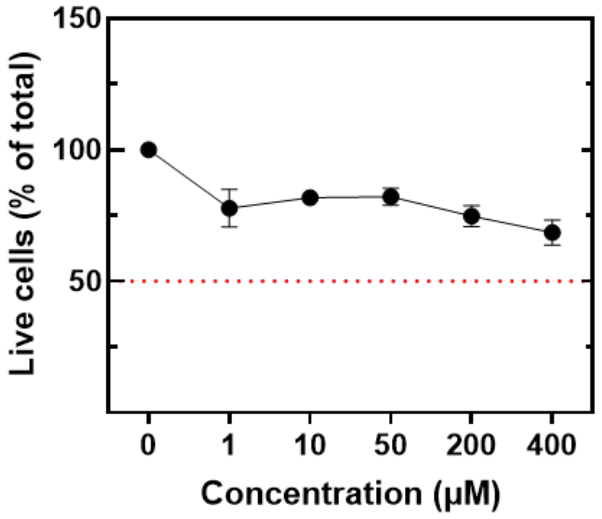

2.25 DDR diagram for compound **7**x3CF_3CO_2H for the MCF-7 cells

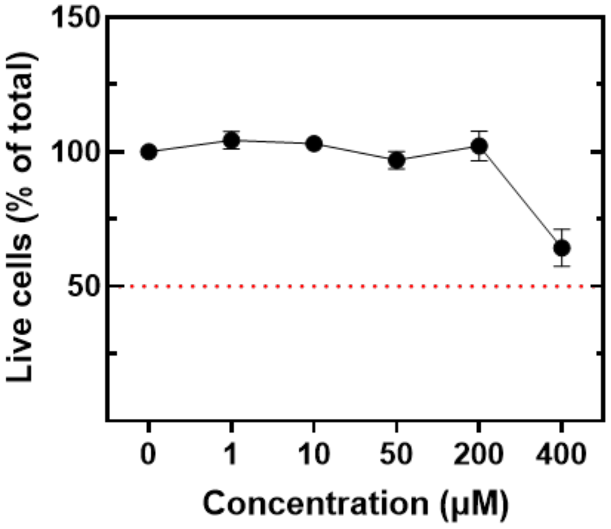

2.26 DDR diagram for compound **7**x3CF_3CO_2H for the MDA-MB-231 cells

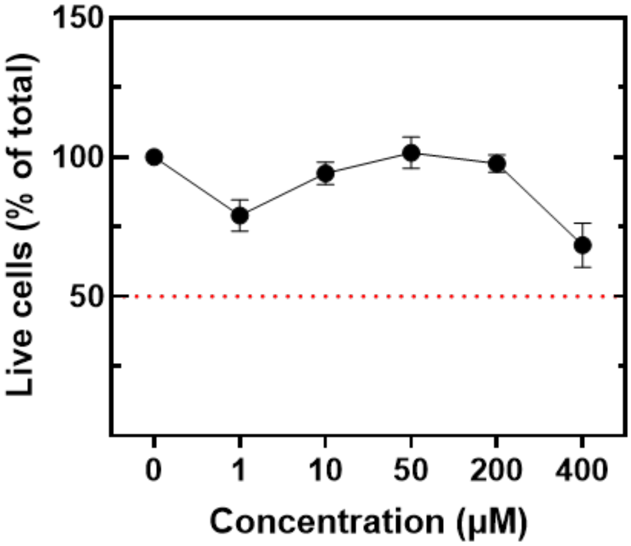

2.27 DDR diagram for compound **8**x4CF_3CO_2H for the MCF-7 cells

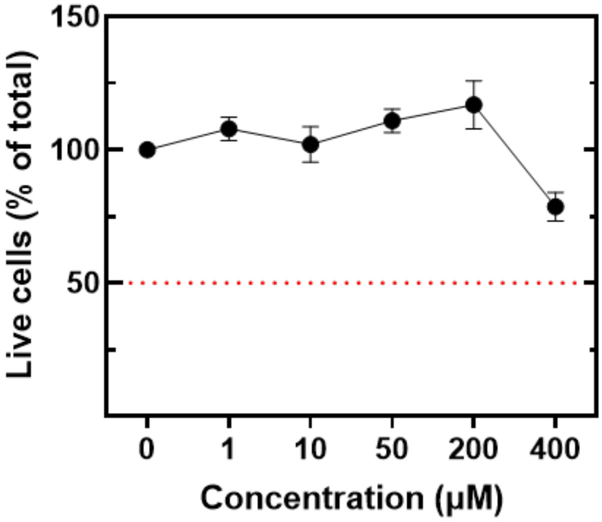

2.28 DDR diagram for compound **8**x4CF_3CO_2H for the MDA-MB-231 cells

3. Experimental protocols

3.1. N^4,N^8-Dinosyl- N^{12}-phthalyl-N^1-tritylthermospermine (**15**)

To a solution of N^4,N^7-dinosyl-N^1-tritylnorspermidine [13] (0.19 g, 0.25 mmol) and *N*-(4-bromobutyl)phthalimide (79 mg, 0.28 mmol) in anhydrous DMF (0.5 mL), K_2CO_3 (83 mg, 0.60 mmol) was added under Ar at ambient temperature. The reaction mixture was vigorously stirred at 60 °C for 2.5 h. Completion of the reaction was verified with TLC using the solvent system N for elution. It was then diluted with H_2O and extracted with ethyl acetate. The organic phase was washed twice with H_2O and once with brine, dried over anhydrous Na_2SO_4, filtered and the filtrate evaporated under reduced pressure. The residue was subjected to FCC using the solvent system K for elution to yield pure product **15** ($C_{49}H_{48}N_6O_{10}S_2$, exact mass: 944.29). Yellow oil (0.19 g, 80%); R_f (K): 0.10; ^{1}H-NMR (600 MHz, $CDCl_3$): δ 8.05-8.00 (m, 2H), 7.88-7.83 (m, 2H), 7.78-7.61 (m, 9H), 7.58-7.52 (m, 2H), 7.51-7.42 (m, 4H), 7.35-7.27 (m, 5H), 7.25-7.18 (m, 3H), 3.68 (t, J = 6.6 Hz, 2H), 3.46-3.26 (m, 8H), 2.13 (br. s, 1H), 1.92-1.82 (m, 2H), 1.76-1.56 (m, 8H) ppm; MS (ESI, 30 eV): *m/z* 967.32 $[M+Na]^+$, 243.52 $[Trt]^+$.

3.2. Preparation of suitably derivatized head groups and fragments

3.2.1. *tert*-Butyl 3-((3-hydroxypropylcarbamoyl)methyl)-1*H*-indole-1-carboxylate (**23**)

To an ice-cooled (0 °C) solution of *N*-Boc-(indol-3-yl)acetic acid (**22**) (0.69 g, 2.5 mmol) and HOSu (0.58 g, 5.0 mmol) in DMF (8 mL), DCC (0.64 g, 3.1 mmol) was added with stirring under Ar. The reaction mixture was stirred at 0 °C for 30 min and at ambient temperature for 2 h whereby the completion of activation was verified by TLC with solvent system Q as eluent. Then, 3-aminopropan-1-ol (0.57 mL, 7.5 mmol) was added to the reaction mixture. Acylation of the amino component was completed within a few minutes, as shown by TLC using solvent system C for elution. Then, a few drops of water and one drop of glacial acetic acid were added, the reaction mixture was further stirred for additional 30 min and then diluted with ethyl acetate. The precipitated urea was filtered off and washed on the filter four times with ice-cold ethyl acetate. The combined filtrates were washed twice with 5% aqueous $NaHCO_3$, twice with H_2O and once with brine, filtered and evaporated to dryness under vacuum. The residue was subjected to FCC using the solvent system B for elution to give pure product **23**. Beige solid (0.66 g, 80%); R_f (B): 0.42; ^{1}H-NMR (600 MHz, $CDCl_3$): δ 8.17 (d, J = 6.0, 1H),

7.57 (s, 1H), 7.51 (d, J = 7.8 Hz, 1H), 7.37 (ddd, J = 7.8, 6.0 and 0.6 Hz, 1H), 7.28 (td, J = 7.8 and 0.6 Hz, 1 H), 5.98 (unresol. t, 1H), 3.70 (s, 2H), 3.55 (q, J = 5.4 Hz, 2H), 3.37 (q, J = 6.0 Hz, 2H), 3.01 (t, J = 5.4 Hz, 1H), 1.69 (s, 9H), 1.58 (quint., J = 6.0 Hz, 2H) ppm; $^{13}C\{^{1}H\}$-NMR (150 MHz, $CDCl_3$): δ 171.5, 149.5, 135.6, 132.4, 129.7, 125.0, 123.0, 118.9, 115.5, 113.6, 84.1, 59.1, 36.3, 33.1, 32.2, 28.2 (3C) ppm; Anal. Calcd (%) for $C_{18}H_{24}N_2O_4$: C, 65.04; H, 7.28; N, 8.43. Found: C, 65.21; H, 7.04; N, 8.23.

3.2.2. Succinimidyl 4-(3-(trifluoromethyl)-3*H*-diazirin-3-yl)benzoate (**29**)

To a cooled (0 °C) solution of acid **28** (0.23 g, 1 mmol) and HOSu (0.23 g, 2 mmol) in anhydrous THF (3 mL), a solution of DCC (0.23 g, 1.1 mmol) in anhydrous THF (1 mL) was added under Ar and exclusion of light. The reaction mixture was stirred at 0 °C for 30 min and at ambient temperature for 2 h. Completion of the reaction was verified with TLC using the solvent system B for elution. Then it was refrigerated overnight and the precipitated urea was filtered off and washed on the filter with ice-cold ethyl acetate. The combined filtrates were then washed twice with ice-cold 5% aqueous $NaHCO_3$ and twice with ice-cold water. The organic phase was dried over anhydrous Na_2SO_4, filtered and evaporated to dryness under vacuum. The residue was subjected to FCC using the solvent system J as eluent to give pure 'active' ester **29**. Beige solid (0.28 g, 87%); M.p. 92-93 °C; R_f(K): 0.30, (B): 0.72; ^{1}H-NMR (600 MHz, $CDCl_3$): δ 8.17 (dt, J = 6.0 and 1.2 Hz, 1H), 7.32 (d, J = 8.4, 1H), 2.91 (s, 4H) ppm; $^{13}C\{^{1}H\}$-NMR (150 MHz, $CDCl_3$): δ 168.9 (2C), 161.0, 135.9 130.9 (2C), 126.8 (2C), 126.3, 121.7 (q, J = 273 Hz), 28.4 (q, J = 40.5 Hz), 25.7 (2C) ppm.

3.2.3. *N*-(4-Bromobutyl)phthalimide

To a solution of *N*-(4-hydroxybutyl)phthalimide (0.99 g, 4.5 mmol) and CBr_4 (2.26 g, 6.8 mmol) in anhydrous DCM (2.5 mL), Ph_3P (1.18 g, 4.5 mmol) was added portion-wise (5 equal portions every 10 min each) at ambient temperature under Ar. Stirring was continued for an additional 1 h, whereby TLC of the reaction mixture with the solvent system O as eluent verified the completion of the reaction. The reaction mixture was then applied directly on a column. FCC using the solvent system K as eluent yielded pure *N*-(4-bromobutyl)phthalimide ($C_{12}H_{12}BrNO_2$, exact mass: 281.01). White solid (1.14 g, 90%); M.p. 77-79 °C; R_f(K): 0.62; ^{1}H-NMR (600 MHz, $CDCl_3$): δ 7.87-7.82 (m, 2H), 7.74-7.70 (m, 2H), 3.73 (t, J = 6.6 Hz, 2H), 3.44 (t, J = 6.6 Hz, 2H), 1.91-

1.88 (m, 4H) ppm; $^{13}C\{^1H\}$-NMR (150 MHz, $CDCl_3$): δ 168.4 (2C), 134.0 (2C), 132.1 (2C), 123.3 (2C), 37.0, 32.8, 29.8, 27.2 ppm; MS (ESI, 30 eV): *m/z* 282.68 and 283.97 $[M+H]^+$.

3.3. Synthesis of polyamine toxins and analogs

3.3.1. PAT analog **1**

3.3.1.1 Synthesis of the fully protected intermediate **30**

To a cooled (0 °C) solution of nosylamide **13** (0.30 g, 0.40 mmol), alcohol **23** (0.17 g, 0.52 mmol) and Ph_3P (0.14g, 0.52 mmol) in anhydrous THF (1.2 mL), DIAD (0.10 mL, 0.52 mmol) was added under Ar. The reaction mixture was then stirred at 0 °C for 15 min and at ambient temperature for 2 h. The solvent was evaporated under reduced pressure and the residue was subjected to FCC using the solvent system K for elution to yield pure intermediate **30** ($C_{56}H_{61}N_7O_{11}S_2$, exact mass: 1071.39). Slightly yellow oil (0.21 g, 49%); R_f (K): 0.42; MS (ESI, 30 eV): *m/z* 1072.42 $[M+H]^+$.

3.3.1.2 Denosylation of intermediate **30**

To a solution of intermediate **30** (0.21 g, 0.2 mmol) in anhydrous DMF (2 mL), Na_2CO_3 (0.21 g, 2 mmol) and PhSH (0.16 mL, 1.6 mmol) were added sequentially under Ar. The reaction mixture was vigorously stirred at ambient temperature overnight. The completion of the reaction was verified with TLC using the solvent systems N or F as eluents. The reaction mixture was then diluted with H_2O and extracted twice with ethyl acetate. The combined organic layers are washed once with 5% aqueous $NaHCO_3$ and once with H_2O, dried over anhydrous Na_2SO_4, filtered and evaporated under reduced pressure. The residue was subjected to FCC using the solvent system F for elution to yield pure denosylated intermediate **31** ($C_{44}H_{55}N_5O_3$, exact mass: 701.43). Slightly yellow oil (86 mg, 61%); R_f (F): 0.12; ^1H-NMR (600 MHz, $CDCl_3$): δ 8.16 (br. s, 1H), 7.56 (s, 1H), 7.53 (d, J = 7.8 Hz, 1H), 7.50-7.44 (m, 6H), 7.34 (t, J = 6.0 Hz, 1H), 7.31-7.23 (m, 6H), 7.26 (t, J = 6.0 Hz, 1H), 7.22-7.17 (m, 3H), 6.74 (unresolv. t, 1H), 3.65 (s, 2H), 3.31 (q, J = 6.0 Hz, 2H), 2.69 (t, J = 6.0 Hz, 2H), 2.58 (t, J = 6.0 Hz, 2H), 2.53 (t, J = 6.0 Hz, 2H), 2.41 (t, J = 6.0 Hz, 2H), 2.21 (t, J = 6.0 Hz, 2H), 2.06 (s, 1H), 1.68 (s, 11H), 1.56 (quint., J = 6.0 Hz, 2H), 1.45 (quint., J = 6.0 Hz, 2H), 1.36 (quint., J = 6.0 Hz, 2H), 1.30-1.26 (m, 2H) ppm; $^{13}C\{^1H\}$-NMR (150 MHz, $CDCl_3$): δ 170.2 (2C), 146.1 (3C), 128.6 (6C), 127.8 (6C), 126.2 (3C), 124.8 (1C), 122.8 (2C), 119.1 (2C), 115.4 (2C), 114.1, 70.9, 49.7, 49.5, 48.3, 42.1 (2C), 38.7,

33.3 (2C), 30.8, 28.7, 28.2 (3C), 27.8, 27.5 ppm; MS (ESI, 30 eV): *m/z* 702.66 $[M+H]^+$, 243.74 $[Trt^+]$.

3.3.1.3 Complete deprotection of intermediate **31** – PAT analog **1**

A cold (0 °C) solution of TFA (0.10 mL, 1.3 mmol) and PhSH (0.12 mL, 1.2 mmol) in DCM (0.90 mL) was added to the intermediate **31** (84 mg, 0.12 mmol) and the resulting solution was kept at ambient temperature for 1, whereby completion of deprotection was verified by TLC using the solvent system I as eluent. Evaporation under reduced pressure left a residue which upon addition of diethyl ether and overnight refrigeration yielded a white precipitate. The supernatant liquor was decanted, fresh diethyl ether was added to wash the precipitate, the supernatant liquor was again decanted and the solid residue was dried overnight, under reduced pressure over P_2O_5, to yield pure product **1** ($C_{20}H_{33}N_5O$, exact mass: 359.27) as the corresponding tetratrifluoroacetate salt. White solid (52 mg, 53%); RP-HPLC: t_R = 10.02 min; ^{1}H-NMR (600 MHz, $(CD_3)_2SO$): δ 10.90 (s, 1H), 8.08 (t, J = 5.4 Hz, 1H), 7.52 (d, J = 7.8 Hz, 1H), 7.33 (d, J = 8.4 Hz, 1H), 7.18 (d, J = 1.8 Hz, 1H), 7.05 (t, J = 7.2 Hz, 1H), 6.95 (t, J = 7.8 Hz, 1H), 3.50 (s, 2H), 3.11 (q, J = 7.2 Hz, 2H), 2.96 (q, J = 6.6 Hz, 2H), 2.90-2.84 (m, 4H), 2.83-2.78 (m, 4H), 2.49-2.48 (m, 2H), 1.89 (quint., J = 7.2 Hz, 2H), 1.70 (quint., J = 7.2 Hz, 2H), 1.52-1.32 (m, 4H) ppm; $^{13}C\{^1H\}$-NMR (150 MHz, $(CD_3)_2SO$): δ 171.9, 136.6, 127.6, 124.4, 121.4, 119.1, 118.8, 111.9, 109.2, 46.6, 46.5, 45.0, 44.4, 36.7, 36.2, 33.2, 26.5, 24.3, 23.1 (2C) ppm; Anal. Calcd (%) for $C_{28}H_{37}F_{12}N_5O_9$: C, 41.23; H, 4.57; N, 8.59. Found: C, 41.48; H, 4.29; N, 8.33; MS (ESI, 30 eV): *m/z* 360.99 $[M+H]^+$.

3.3.2. PAT analog **2**

3.3.2.1 Synthesis of intermediate (**33**)

To a cooled (0 °C) solution of (indol-3-yl)acetic acid (**21**) (0.13 g, 0.77 mmol) and HOSu (0.18 g, 1.54 mmol) in dry DMF (3 mL), DCC (0.17g, 0.84 mmol) was added under Ar. The reaction mixture was stirred at 0 °C for 30 min and 2 h at ambient temperature and then transferred to a flask containing the Spd derivative **32** (0.40 g, 0.70 mmol), followed by the addition of Et_3N (0.15 mL, 1.08 mmol). The reaction mixture was further stirred at ambient temperature for 2 h, whereby completion of coupling was verified by TLC using the solvent system F for elution, and then diluted with ethyl acetate. The organic phase was washed twice with 5% aqueous $NaHCO_3$, twice with H_2O and once with brine, dried over anhydrous Na_2SO_4, filtered and finally

evaporated under reduced pressure. The residue was subjected to FCC using solvent R as eluent to yield pure product **33** ($C_{42}H_{43}N_5O_5S$, exact mass: 729.30). Slightly yellow oil (0.33g, 65%); R_f (Q): 0.30; ^{1}H-NMR (600 MHz, $CDCl_3$): δ 8.22 (br. s, 1H), 7.94 (dd, J = 9.0 and 1.2 Hz, 1H), 7.63 (quint.d, J = 7.8 and 1.2 Hz, 2H), 7.54 (dd, J = 7.2 and 1.2 Hz, 2H), 7.45-7.41 (m, 6H), 7.38 (d, J = 6.0 Hz, 1H), 7.31-7.25 (m, 6H), 7.25-7.17 (m, 4H), 7.17-7.12 (m, 2H), 5.70 (t, J = 6.2 Hz, 1H), 3.72 (s, 2H), 3.30 (t, J = 7.8 Hz, 2H), 3.20-314 (m, 4H), 2.08 (t, J = 6.6 Hz, 2H), 1.67-1.58 (m, 3H), 1.42 (quint., J = 6.6 Hz, 2H), 1.34 (quint., J = 7.2 Hz, 2H) ppm; MS (ESI, 30 eV): *m/z* 730.27 [M+H]+.

3.3.2.2 Denosylation of intermediate **33**

To a solution of intermediate **33** (0.32 g, 0.44 mmol) in anhydrous DMF (4 mL), Na_2CO_3 (0.23 g, 2.2 mmol) and PhSH (0.18 mL, 1.76 mmol) were added sequentially under Ar. The reaction mixture was vigorously stirred at ambient temperature overnight. The completion of the reaction was verified with TLC using the solvent systems Q or F. The reaction mixture was then diluted with H_2O and extracted twice with ethyl acetate. The combined organic layers are washed once with 5% aqueous $NaHCO_3$ and once with H_2O, dried over anhydrous Na_2SO_4, filtered and evaporated under reduced pressure. The residue was subjected to FCC using the solvent system F for elution to yield pure denosylated intermediate **34**. Slightly yellow oil (0.11 g, 46%); R_f (F): 0.16; ^{1}H-NMR (600 MHz, $CDCl_3$): δ 8.43 (br. s, 1H), 7.55 (d, J = 7.8 Hz, 1H), 7.49-7.44 (m, 6H), 7.37 (d, J = 8.4 Hz, 1H), 7.30-7.25 (m, 6H), 7.24-7.17 (m, 3H), 7.15-7.11 (m, 1H), 7.05 (unresolv. d, 1H), 5.96 (unresolv. t, 1H), 3.71 (s, 2H), 3.18 (q, J = 6.0 Hz, 2H), 2.57 (t, J = 6.6 Hz, 2H), 2.48 (t, J = 6.6 Hz, 2H), 2.17 (t, J = 6.6 Hz, 2H), 1.61 (quint., J = 6.6 Hz, 2H), 1.40 (quint., J = 7.2 Hz, 2H), 1.34 (quint., J = 7.2 Hz, 2H) ppm; ^{13}C{^{1}H}-NMR (150 MHz, $CDCl_3$): δ 171.5, 146.2 (3C), 136.4, 128.7 (6C), 127.8 (6C), 127.1, 126.3 (3C), 123.8, 122.6, 120.0, 118.7, 111.4, 109.1, 70.9, 49.4, 48.3, 42.1, 39.3, 33.4, 30.8, 27.3, 27.2 ppm.

3.3.2.3 Deprotection of intermediate **34** – PAT analog **2**

A cold (0 °C) solution of TFA (0.15 mL, 2.0 mmol) and PhSH (0.10 mL, 1.0 mmol) in DCM (1.40 mL) was added to the intermediate **34** (0.11 g, 0.2 mmol) and the resulting solution was kept at ambient temperature for 1, whereby completion of deprotection was verified by TLC using the solvent system I as eluent. Evaporation under reduced pressure left a residue which upon addition of diethyl ether and overnight refrigeration yielded a white precipitate. The supernatant liquor was decanted, fresh

diethyl ether was added to wash the precipitate, the supernatant liquor was again decanted and the solid residue was dried overnight, under reduced pressure over P_2O_5, to yield pure product **2** ($C_{17}H_{26}N_4O$, exact mass: 302.21) as the corresponding tritrifluoroacetate salt. White solid (71 mg, 55%); RP-HPLC: t_R = 10.49 min; ^{1}H-NMR (600 MHz, $(CD_3)_2SO$): δ 10.86 (br. s, 1H), 8.59 (unresolv. t, 1H), 7.93-7.89 (m, 4H), 7.52 (d, J = 7.8 Hz, 1H), 7.32 (d, J = 8.4 Hz, 1H), 7.16 (m, 1H), 7.04 (t, J = 7.2 Hz, 1H), 6.95 (t, J = 7.8 Hz, 1H), 3.47 (s, 2H), 3.04 (q, J = 6.0 Hz, 2H), 2.93-2.89 (m, 2H), 2.87-2.82 (m, 4H), 1.85 (quint., J = 7.2 Hz, 2H), 1.53 (quint., J = 7.2 Hz, 2H), 1.42 (quint., J = 7.2 Hz, 2H), ppm; $^{13}C\{^1H\}$-NMR (150 MHz, $(CD_3)_2SO$): δ 171.2, 136.6, 127.7, 124.2, 121.4, 119.1, 118.7, 111.8, 109.3, 47.0, 44.3, 38.4, 36.7, 33.2, 26.8, 24.2, 23.4 ppm; MS (ESI, 30 eV): *m/z* 303.64 $[M+H]^+$. Anal. Calcd (%) for $C_{23}H_{29}F_9N_4O_7$: C, 42.86; H, 4.54; N, 8.69. Found: C, 43.01; H, 4.33; N, 8.38.

3.3.3. PAT analog **3**

3.3.3.1 Synthesis of intermediate **35**

To a cooled (0 °C) solution of (indol-3-yl)acetic acid (**21**) (0.19 g, 1.1 mmol) and HOSu (0.25 g, 2.2 mmol) in dry DMF (4.5 mL), DCC (0.25 g, 1.2 mmol) was added under Ar. The reaction mixture was stirred at 0 °C for 30 min and 2 h at ambient temperature and then transferred to a flask containing the Put derivative **11** (0.33 g, 1.0 mmol), followed by the addition of Et_3N (0.21 mL, 1.5 mmol). The reaction mixture was further stirred at ambient temperature for 2 h, whereby completion of coupling was verified by TLC using the solvent system F for elution, and then diluted with ethyl acetate. The organic phase was washed twice with 5% aqueous $NaHCO_3$, twice with H_2O and once with brine, dried over anhydrous Na_2SO_4, filtered and finally evaporated under reduced pressure. The residue was subjected to FCC using solvent R as eluent to yield pure product **35** ($C_{33}H_{33}N_3O$, exact mass: 487.26). Slightly yellow oil (0.37 g, 75%); R_f(N): 0.32; ^{1}H-NMR (600 MHz, $CDCl_3$): δ 8.28 (br. s, 1H), 7.55 (d, J = 7.8 Hz, 1H), 7.42-7.40 (m, 6H), 7.38 (d, J = 7.8 Hz, 1H), 7.28-7.24 (m, 8H), 7.22-7.15 (m, 4H), 5.67 (unresolv. t, 1H), 3.73 (s, 2H), 3.13 (q, J = 6.6 Hz, 2H), 2.03 (t, J = 6.0 Hz, 2H), 1.39 (quint., J = 7.2 Hz, 3H), 1.33 (quint., J = 7.2 Hz, 2H) ppm; $^{13}C\{^1H\}$-NMR (150 MHz, $CDCl_3$): δ 171.4, 146.2 (3C), 136.4, 128.6 (6C), 127.8 (6C), 127.0, 126.2 (3C), 123.7, 122.7, 120.1, 118.8, 111.4, 109.2, 70.8, 43.2, 39.5, 33.5, 28.1, 27.5 ppm; MS (ESI, 30 eV): *m/z* 974.14 [2M]+, 488.18 $[M+H]^+$, 243.13 $[Trt]^+$.

3.3.3.2 Deprotection of intermediate **35** – PAT analog **3**

A cold (0 °C) solution of TFA (0.6 mL, 7.90 mmol) and PhSH (0.4 mL, 3.95 mmol) in DCM (5.4 mL) was added to the intermediate **35** (0.37 g, 0.75 mmol) and the resulting solution was kept at ambient temperature for 1, whereby completion of deprotection was verified by TLC using the solvent system I as eluent. Evaporation under reduced pressure left a residue which upon addition of diethyl ether and overnight refrigeration yielded a white precipitate. The supernatant liquor was decanted, fresh diethyl ether was added to wash the precipitate, the supernatant liquor was again decanted and the solid residue was dried overnight, under reduced pressure over P_2O_5, to yield pure product **3** ($C_{14}H_{19}N_3O$, exact mass: 245.15) as the corresponding tritrifluoroacetate salt. Brown oil (0.24 g, 67%); RP-HPLC: t_R = 10.69 min; ^{1}H-NMR (600 MHz, $(CD_3)_2SO$): δ 10.88 (br. s, 1H), 7.92 (t, J = 5.4 Hz, 1H), 7.74 (br. s, 3H), 7.53 (d, J = 7.8 Hz, 1H), 7.34 (d, J = 8.4 Hz, 1H), 7.18 (unreslov. d, 1H), 7.06 (t, J = 7.2 Hz, 1H), 6.97 (t, J = 7.2 Hz, 1H), 3.49 (s, 2H), 3.05 (q, J = 6.0 Hz, 2H), 2.77 (q, J = 6.0 Hz, 2H), 1.51 (quint., J = 7.8 Hz, 2H), 1.43 (quint., J = 7.2 Hz, 2H) ppm; ^{13}C{^{1}H}-NMR (150 MHz, $(CD_3)_2SO$): δ 171.2, 158.6 (q, J = 32.3 Hz, 2C) 136.6, 127.7, 124.2, 121.4, 119.1, 118.7, 118.3 (q, J = 284.0 Hz, 2C), 111.8, 109.3, 39.0, 38.5, 33.2, 26.7, 25.0 ppm; MS (ESI, 30 eV): *m/z* 268.60 $[M+Na]^+$, 246.49 $[M+H]^+$, 229.43 $[M+H-NH_3]^+$.

3.3.4. PAT analog **4**

3.3.4.1 Removal of the Phth group from compound **15** - Preparation of N^4,N^8-Nosyl-N^1-tritylthermospermine (**36**)

To a solution of Tsm derivative **15** (0.28 g, 0.3 mmol) in ethanol (2.5 mL), $H_2NNH_2{\cdot}H_2O$ (30 μL, 0.6 mmol) was added and the reaction mixture was refluxed for 1 h. Additional $H_2NNH_2{\cdot}H_2O$ (30 μL) was added and the reaction mixture was further refluxed for 1 h, whereby completion of the reaction was verified with TLC using the system K as eluent. The solvent was evaporated under reduced pressure, 5% aqueous $NaHCO_3$ was added to the residue and the aqueous phase was extracted twice with DCM. The combined organic phases were washed once with brine, dried over anhydrous Na_2SO_4, filtered and evaporated to dryness. The residue was subjected to FCC using initially the solvent F as eluent to yield pure product **36**. Slightly yellow oil (0.17g, 71%); R_f (F): 0.16; ^{1}H NMR (600 MHz, $CDCl_3$): δ 8.02-7.96 (m, 2H), 7.73-7.65 (m, 4H), 7.65-7.60 (m, 1H), 7.60-7.53 (m, 1H), 7.47-7.42 (m, 6H), 7.32-7.27 (m,

6H), 7.23-7.19 (m, 3H), 3.43-3.35 (m, 2H), 3.34-3.26 (m, 7H), 3.17 (q, *J* = 6.0 Hz, 1H), 2.93 (unresolv. t, 1H), 2.14-2.07 (m, 2H), 1.92-1.87 (quint., *J* = 6.6 Hz, 1H), 1.87-1.80 (quint., *J* = 7.2 Hz, 1H), 1.73-1.64 (m, 5H), 1.64-1.58 (m, 1H), 1.49-1.44 (m, 1H), 1.30 (br. s, 1H) ppm.

3.3.4.2 Coupling of amine **36** and Fmoc-(L)-Thr(^{t}Bu)-OH (**25**) – Synthesis of amide **37**

To a cooled (0 °C) solution of amine **36** (0.16 g, 0.20 mmol) and DIPEA (80 μL, 0.46 mmol) in DMF (0.5 mL), the carboxylic acid **25** (90 mg, 0.23 mmol) was added followed by the coupling agent HBTU (0.11 g, 0.28 mmol). The reaction mixture was then brought to ambient temperature were it was stirred for 30 min. The completion of the reaction was verified by TLC with the solvent system F. The reaction mixture was then diluted with ethyl acetate and the organic phase was washed once with 5% aqueous $NaHCO_3$, twice with H_2O and once with brine. It was finally dried over anhydrous Na_2SO_4, filtered and evaporated to dryness. The residue was then subjected to FCC using the solvent system N for elution to give pure amide **37** ($C_{64}H_{71}N_7O_{12}S_2$, exact mass: 1193.46). Cream oil (0.19 g, 81%); R_f (N): 0.26; ^{1}H NMR (600 MHz, $CDCl_3$): *δ* 8.03 (dd, *J* = 7.8 and 4.8 Hz, 2H), 7.81 (*J* = 7.8 Hz, 2H), 7.73-7.67 (m, 4H), 7.65 (dd, *J* = 7.8 and 4.8 Hz, 2H), 7.64-7.56 (m, 4H), 7.48-7.42 (m, 2H), 7.35 (t, *J* = 7.8 Hz, 4H), 7.32-7.28 (m, 6H), 7.24-7.19 (m, 3H), 7.03 (unresolv. t, 1H), 6.03 (unresolv. d, 1H), 4.44 (m, 2H), 4.27 (t, *J* = 6 Hz, 1H), 4.30-4.24 (unresolv. t, 1H), 4.22-4.17 (m, 1H), 4.14 (unresolv. t, 1H), 3.40 (t *J* = 7.2 Hz, 2H), 3.37-3.25 (m, 8H), 2.12 (t *J* = 4.8 Hz, 2H), 1.88 (t, *J* = 5.4 Hz, 2H), 1.69 (unresolv. t, 2H), 1.57-1.52 (m, 2H), 1.31 (s, 9H), 1.06 (d, *J* =6.0 Hz, 3H), 0.93-0.87 (m, 3H) ppm. ^{13}C{^{1}H}-NMR (150 MHz, $CDCl_3$): *δ* 148.0, 145.9 (3C), 143.9, 143.7, 141.3, 133.6 (2C), 133.2, 131.8 (2C), 130.8, 128.6 (6C), 127.8 (6C), 127.7 (2C), 127.1 (2C), 126.3 (3C), 125.1, 124.2, 124.1 (2C), 121.4, 120.0, 119.9 (2C), 118.0, 70.9, 66.9, 63.5, 47.2, 46.0, 44.9, 29.7 (2C), 29.6, 29.4, 28.2 (2C), 25.6, 22.7 (3C), 20.3, 17.1, 14.1 (2C) ppm; MS (ESI, 30 eV): *m/z* 1216.94 $[M+Na]^+$, 1193.98 $[M+H]^+$, 243.81 $[Trt^+]$.

3.3.4.3 Removal of the Fmoc group from compound **37** - Preparation of intermediate **38**

A solution made up of Et_2NH (0.5 mL) and DCM (1.5 mL) was added to compound **37** (0.12 g, 0.1 mmol) under Ar and the resulting reaction mixture was stirred at ambient temperature for 1 h. The completion of the reaction was verified with TLC using the solvent system N as eluent. The reaction mixture was diluted with DCM and the organic

phase was washed thrice with H_2O. Drying over anhydrous Na_2SO_4 followed by filtration and evaporation of the solvent under reduced pressure left an oily residue, from which pure amine **38** ($C_{49}H_{61}N_7O_{10}S_2$, exact mass: 971.39) was obtained through FCC using the solvent system E for elution. Colorless oil (76 mg, 78%); R_f (E): 0.20; MS (ESI, 30 eV): *m/z* 1010.58 $[M+K]^+$, 994.60 $[M+Na]^+$, 972.58 $[M+H]^+$.

3.3.4.4 Coupling of amine **38** and butanoic acid – Synthesis of bisamide **39**

To a cooled (0 °C) solution of amine **38** (76 mg, 0.078 mmol) and DIPEA (50 μL, 0.28 mmol) in DMF (0.5 mL), butanoic acid (18 μL, 0.195 mmol) was added followed by the coupling agent HBTU (74 mg, 0.195 mmol). The reaction mixture was allowed to attain ambient temperature were it was stirred for 30 min. The completion of the reaction was verified by TLC with the solvent system F. The reaction mixture was then diluted with ethyl acetate and the organic phase was washed once with 5% aqueous $NaHCO_3$, twice with H_2O and once with brine. It was finally dried over anhydrous Na_2SO_4, filtered and evaporated to dryness. The residue was then subjected to FCC using the eluent R to give pure bisamide **39** ($C_{53}H_{67}N_7O_{11}S_2$, exact mass: 1041.43). Colorless oil (50 mg, 61%); R_f (R): 0.40; ^{1}H NMR (600 MHz, $CDCl_3$): δ 8.08-8.04 (m, 2H), 7.73-7.66 (m, 4H), 7.64-7.60 (m, 2H), 7.47-7.42 (m, 6H), 7.33-7.28 (m, 6H), 7.25-7.20 (m, 3H), 7.15 (unresolv. t, 1H), 6.63 (d, J = 4.8 Hz, 1H), 4.34 (unresolv. t, 1H), 4.28-4.23 (m, 1H), 3.43 (m, 11H), 2.27 (t, J = 7.8 Hz, 2H), 1.81 (quint., J = 6.6 Hz, 3H), 1.75-1.64 (m, 6H), 1.59-1.50 (m, 4H), 1.29 (*d*, J = 9.6 Hz, 3H), 1.28 (s, 9H), 0.99 (t, J = 7.2 Hz 3H) ppm. $^{13}C\{^1H\}$-NMR (150 MHz, $CDCl_3$): δ 173.1, 170.0, 148.1, 148.0 (2C), 145.9 (2C), 133.6, 133.4, 133.2, 131.8 (2C), 131.6, 130.7, 130.6, 128.5 (6C), 127.8 (6C), 126.3 (2C), 124.2 (3C), 75.3, 70.9, 66.0, 57.7, 47.1, 46.6, 45.5, 45.2, 40.9, 38.4, 32.0, 29.7, 29.6, 29.4, 29.3, 28.2 (3C), 25.1, 18.9, 13.8 ppm; MS (ESI, 30 eV): *m/z* 1080.54 $[M+K]^+$, 1064.49 $[M+Na]^+$, 243.67 $[Trt]^+$.

3.3.4.5 Denosylation of bisamide **39**

To a solution of bisamide **39** (50 mg, 0.048 mmol) in anhydrous DMF (0.5 mL), Na_2CO_3 (50 mg, 0.48 mmol) and PhSH (40 μL, 0.39 mmol) were added sequentially under Ar. The reaction mixture was vigorously stirred at ambient temperature overnight. The completion of the reaction was verified with TLC using the solvent systems F. The reaction mixture was then diluted with H_2O and extracted twice with ethyl acetate. The combined organic layers are washed once with 5% aqueous $NaHCO_3$ and once with H_2O, dried over anhydrous Na_2SO_4, filtered and evaporated under reduced pressure. The residue was subjected to FCC using the solvent system G for

elution to yield pure denosylated intermediate **40** ($C_{41}H_{61}N_5O_3$, exact mass: 671.48). Slightly yellow oil (16 mg, 51%); R*f* (G): 0.24; MS (ESI, 30 eV): *m/z* 694.67 $[M+Na]^+$, 672.58 $[M+H]^+$, 244.46 $[TrtH]^{\cdot+}$.

3.3.4.6 Deprotection of intermediate **40** – PAT analog 4

A cold (0 °C) solution of TFA (0.3 mL, 3.92 mmol) and PhSH (20 μL, 0.196 mmol) in DCM (0.3 mL) was added to the intermediate **40** (16 mg, 0.024 mmol) and the resulting solution was kept at ambient temperature for 1, whereby completion of deprotection was verified by TLC using the solvent system I as eluent. Evaporation under reduced pressure left a residue which upon addition of diethyl ether and overnight refrigeration yielded a white precipitate. The supernatant liquor was decanted, fresh diethyl ether was added to wash the precipitate, the supernatant liquor was again decanted and the solid residue was dried overnight, under reduced pressure over KOH pellets, to yield pure product **4** ($C_{18}H_{39}N_5O_3$, exact mass: 373.31) as the corresponding tritrifluoroacetate salt. White solid (15 mg, 89%); MS (ESI, 30 eV): *m/z* 374.95 $[M+H^+]$. Anal. Calcd (%) for $C_{24}H_{42}F_9N_5O_9$: C, 40.28; H, 5.92; N, 9.79. Found: C, 40.01; H, 6.16; N, 9.97.

3.3.5. PAT analog **5**

3.3.5.1 Coupling of amine **53** and Fmoc-(L)-Thr(tBu)-OH (**25**) – Synthesis of amide **54**

To a cooled (0 °C) solution of amine **53** (0.16 g, 0.20 mmol) and DIPEA (80 μL, 0.46 mmol) in DMF (0.5 mL), the carboxylic acid **25** (90 mg, 0.23 mmol) was added followed by the coupling agent HBTU (0.11 g, 0.28 mmol). The reaction mixture was then brought to ambient temperature were it was stirred for 30 min. The completion of the reaction was verified by TLC with the solvent system F. The reaction mixture was then diluted with ethyl acetate and the organic phase was washed once with 5% aqueous $NaHCO_3$, twice with H_2O and once with brine. It was finally dried over anhydrous Na_2SO_4, filtered and evaporated to dryness. The residue was then subjected to FCC using the solvent system N for elution to give pure adduct **54** ($C_{64}H_{71}N_7O_{12}S_2$, exact mass: 1193.46). Slightly yellow oil (0.19 g, 78%); R*f* (N): 0.26; MS (ESI, 30 eV): *m/z* 1216.51 $[M+Na]^+$, 243.59 $[Trt]^+$.

3.3.5.2 Removal of the Fmoc group from compound **54** - Preparation of intermediate **55**

A solution made up of Et_2NH (0.8 mL) and DCM (2.4 mL) was added to compound **54** (0.19 g, 0.16 mmol) under Ar and the resulting reaction mixture was stirred at ambient temperature for 1 h. The completion of the reaction was verified with TLC using the solvent system N as eluent. The reaction mixture was diluted with DCM and the organic phase was washed thrice with H_2O. Drying over anhydrous Na_2SO_4 followed by filtration and evaporation of the solvent under reduced pressure left an oily residue, from which pure amine **55** was obtained through FCC using the solvent system E for elution. Colorless oil (0.14 g, 90%); R_f (E): 0.20.

3.3.5.3 Coupling of amine **55** and butanoic acid – Synthesis of bisamide **56**

To a cooled (0 °C) solution of amine **55** (0.14 g, 0.14 mmol) and DIPEA (70 μL, 0.42 mmol) in DMF (0.5 mL), butanoic acid (17 μL, 0.18 mmol) was added followed by the coupling agent HBTU (68 mg, 0.18 mmol). The reaction mixture was allowed to attain ambient temperature where it was stirred for 30 min. The completion of the reaction was verified by TLC with the solvent system E. The reaction mixture was then diluted with ethyl acetate and the organic phase was washed once with 5% aqueous $NaHCO_3$, twice with H_2O and once with brine. It was finally dried over anhydrous Na_2SO_4, filtered and evaporated to dryness. The residue was then subjected to FCC using the eluent R to give pure bisamide **56** ($C_{53}H_{67}N_7O_{11}S_2$, exact mass: 1041.43). Colorless oil (85 mg, 58%); R_f (EtOAc (100%): 0.40; ^{1}H NMR (600 MHz, $CDCl_3$): δ 8.04-7.99 (m, 2H), 7.73-7.69 (m, 3H), 7.68 (t, J = 7.2 Hz, 1H), 7.64-7.60 (m, 2H), 7.48-7.42 (m, 6H), 7.32-7.28 (m, 6H), 7.25-7.20 (m, 3H), 7.15 (unresolv. t, 1H), 6.63 (d, J = 8.0 Hz, 1H), 4.34 (unresolv. t, 1H), 4.25 (unresolv. quint., 1H), 3.42-3.28 (m, 10H), 2.27 (t, J = 7.8 Hz, 2H), 2.13-2.09 (m, 2H), 1.81 (quint., J = 6.6 Hz, 3H), 1.75-1.66 (m, 6H), 1.58-1.55 (m, 2H), 1.29 (d, J = 9.6 Hz, 3H), 1.28 (s, 9H), 0.98 (t, J = 7.2 Hz 3H) ppm. $^{13}C\{^1H\}$ NMR (150 MHz, $CDCl_3$): δ 173.1, 170.0, 148.0, 147.9, 145.9 (3C), 133.6, 133.4, 133.2, 131.8 (2C), 131.6, 130.7, 130.6, 128.5 (6C), 127.8 (6C), 126.3 (2C), 124.1 (3C), 75.3, 70.9, 66.0, 57.7, 47.1, 46.6, 45.5, 45.2, 40.9, 38.4, 36.3, 31.9, 29.7, 28.2 (3C), 25.1, 22.7, 18.9, 17.8, 13.8 ppm; MS (ESI, 30 eV): *m/z* 1080.54 $[M+K]^+$, 1064.49 $[M+Na]^+$, 243.67 $[Trt]^+$.

3.3.5.4 Denosylation of bisamide **56**

To a solution of bisamide **56** (85 mg, 0.082 mmol) in anhydrous DMF (0.8 mL), Na_2CO_3 (85 mg, 0.8 mmol) and PhSH (65 μL, 0.64 mmol) were added sequentially under Ar. The reaction mixture was vigorously stirred at ambient temperature overnight. The completion of the reaction was verified with TLC using the solvent

systems F. The reaction mixture was then diluted with H_2O and extracted twice with ethyl acetate. The combined organic layers are washed once with 5% aqueous $NaHCO_3$ and once with H_2O, dried over anhydrous Na_2SO_4, filtered and evaporated under reduced pressure. The residue was subjected to FCC using the solvent system G for elution to yield pure denosylated intermediate **57** ($C_{41}H_{61}N_5O_3$, exact mass: 671.48). Colorless oil (40 mg, 72%); R_f(G): 0.24; MS (ESI, 30 eV): *m/z* 694.67 $[M+Na]^+$, 672.58 $[M+H]^+$, 244.46$[TrtH]^{\cdot+}$.

3.3.5.5 Deprotection of intermediate **57** – PAT analog **5**

A cold (0 °C) solution of TFA (0.4 mL, 5.22 mmol) and PhSH (30 μL, 0.29 mmol) in DCM (0.4 mL) was added to the intermediate **57** (40 mg, 0.06 mmol) and the resulting solution was kept at ambient temperature for 1, whereby completion of deprotection was verified by TLC using the solvent system I as eluent. Evaporation under reduced pressure left a residue which upon addition of diethyl ether and overnight refrigeration yielded a white precipitate. The supernatant liquor was decanted, fresh diethyl ether was added to wash the precipitate, the supernatant liquor was again decanted and the solid residue was dried overnight, under reduced pressure over KOH pellets, to yield pure product **5** ($C_{18}H_{39}N_5O_3$, exact mass: 373.31) as the corresponding tritrifluoroacetate salt. White solid (37 mg, 87%); MS (ESI, 30 eV): *m/z* 374.95 $[M+H]^+$. Anal. Calcd (%) for $C_{24}H_{42}F_9N_5O_9$: C, 40.28; H, 5.92; N, 9.79. Found: C, 40.07; H, 6.10; N, 10.01.

3.3.6. PAT analog **9**

3.3.6.1 Selective removal of the Trt group from the Tsm derivative **17** – Preparation of intermediate **41**

A cold (0 °C) solution consisted of TFA (1 mL) and DCM (9 mL) was added to the TSM derivative **17** (0.50 g, 0.51 mmol) and the resulting reaction mixture was kept at ambient temperature for 1 h whereby the completion of the reaction was verified with TLC using L as eluent. Volatiles were then evaporated under reduced pressure and dry diethyl ether was added to the residue. Upon overnight refrigeration an oil separated. The supernatant solvent was decanted and fresh dry diethyl ether was added to wash the residue. Refrigeration overnight and decantation of the supernatant solvent left an oily residue which was dried over P_2O_5 overnight under vacuo yielding crude intermediate **41**, as the corresponding bistritrifluoroacetate salt (0.44 g), which was used as such into the next coupling experiment.

3.3.6.2 Coupling of amine **41** with Fmoc-(L)-propargylglycine (**27**) – Synthesis of amide **43**

To a cold (0 °C) solution of the bistrifluoroacetate salt of amine **41** (0.44 g, 0.46 mmol) and dry DIPEA (0.4 mL, 2.30 mmol) in anhydrous DMF (1.4 mL), the amino acid derivative **33** (0.17 g, 0.51 mmol) and PyBrOP (0.28 g, 0.60 mmol) were added sequentially under Ar. The reaction mixture was then stirred at ambient temperature for 30 min whereby completion of the reaction was certified with TLC using G as eluent. The reaction mixture was then diluted with ethyl acetate and the organic phase was washed sequentially once with ice-cold 5% aqueous citric acid, once with ice-cold 5% aqueous $NaHCO_3$, twice with H_2O and once with brine. Drying over anhydrous Na_2SO_4, filtration and evaporation of the solvent under reduced pressure left a residue, which was subjected to FCC using the eluent R for elution yielding pure amide **43**. Slightly yellow oil (0.34 g, 70% for the two steps); R_f (R): 0.30; ^{1}H-NMR (600 MHz, $CDCl_3$): δ 13.45 (unresolv. t, 1H), 8.03-8.01 (m, 1H), 7.98-7.95 (m, 1H), 7.76 (d, J = 7.8 Hz, 2H), 7.70-7.65 (m, 4H), 7.63-7.57 (m, 4H), 7.39 (t, J = 7.2 Hz, 2H), 7.30 (t, J = 7.2 Hz, 2H), 6.45 (br. s, 1H), 5.75 (br. s, 1H), 4.36-4.41 (m, 1H), 4.39-4.34 (unresolv. t, 1H), 4.33-4.26 (m, 1H), 4.21 (t, J = 7.2 Hz, 1H), 3.42-3.37 (m, 4H), 3.33-3.25 (m, 8H), 2.81-2.72 (m, 1H), 2.66-2.58 (m, 1H), 2.49 (s, 3H), 2.35 (s, 4H), 2.12 (t, J = 2.4 Hz, 1H), 1.92 (quint., J = 7.2 Hz, 2H), 1.88 (quint., J = 7.8 Hz, 2H), 1.59 (quint., J = 6.0 Hz, 2H), 1.50 (quint., J = 6.0 Hz, 2H), 1.02 (s, 6H) ppm. ^{13}C{^{1}H}-NMR (150 MHz, $CDCl_3$): δ 173.8, 171.1, 170.0, 156.0, 148.0, 147.9, 143.7, 141.3, 133.9, 133.7, 132.8, 132.5, 132.0, 131.8, 131.1, 130.8, 127.8 (2C), 127.6, 127.1, 127.0, 125.1, 124.7, 124.3, 124.2, 120.0 (2C), 108.1, 71.9, 67.2, 65.2, 53.4 (2C), 50.4, 47.7, 47.1, 45.5, 45.3, 40.5, 38.8, 31.9, 30.1, 29.7, 28.3, 28.0 (2C), 27.8, 26.5, 25.6, 17.9 ppm. *Note*: The *C*=O carbons of the Dde protecting group are not observed in the ^{13}C-NMR spectrum.

3.3.6.3 Removal of the Fmoc group from amide **43** – Preparation of intermediate **44**

A solution of Et_2NH (1.8 mL, 17.40 mmol) in DCM (7.2 mL) was added to amide **43** (0.34 g, 0.32 mmol) under Ar and the resulting reaction mixture was kept at ambient temperature for 3 h whereby the completion of the reaction was certified using TLC with eluent R. It was then diluted with additional DCM and the organic phase was washed thrice with H_2O, dried and evaporated under reduced pressure to dryness. The resulting crude amine **44** was used as such into the next experiment.

3.3.6.4 Coupling of amine **44** with 'active' ester **29** – Synthesis of bisamide **45**

To a solution of amine **44** (0.32 mmol) in dry DMF (0.6 mL), DIPEA (70 μL, 0.40 mmol) and the 'active' ester **29** (0.10 g, 0.32 mmol) were added sequentially at ambient temperature under Ar. The reaction mixture was stirred overnight at ambient temperature whereby the completion of the reaction was certified with TLC using the solvent system L. It was then applied directly on the top of a chromatography column and subjected to FCC using the solvent system L for elution yielding pure bisamide **45**. Slightly yellow oil (0.24 g, 71% for the two steps); R_f (L): 0.24; ^{1}H-NMR (600 MHz, $CDCl_3$): δ 13.43 (t, J = 4.2 Hz, 1H), 8.02 (dd, J = 7.8 and 1.8 Hz, 1H), 7.98-7.95 (m, 1H), 7.85 (dt, J = 7.8 and 0.6 Hz, 2H), 7.73-7.67 (m, 4H), 7.63-7.59 (m, 2H), 7.36 (d, J = 7.2 Hz, 1H), 7.23 (d, J = 8.4 Hz, 2H), 6.72 (t, J = 6.0 Hz, 1H), 4.71 (td, J = 7.2 and 6.6 Hz, 1H), 3.44-3.37 (m, 4H), 3.33-3.24 (m, 6H), 3.34 (br. s, 4H), 2.12 (*t*, J = 2.4 Hz, 1H), 1.92 (quint., J = 7.2 Hz, 2H), 1.88 (quint., J = 7.2 Hz, 2H), 1.72 (br. s, 3H), 1.60 (quint., J = 7.2 Hz, 2H), 1.52 (quint., J = 7.2 Hz, 2H), 1.27-1.23 (m, 4H), 1.02 (s, 6H) ppm. ^{13}C{^{1}H}-NMR (150 MHz, $CDCl_3$): δ 173.8, 171.2, 169.8, 166.1, 148.1, 148.0, 134.7, 134.0, 133.7, 132.8, 132.7, 132.6, 132.0, 131.9, 131.0, 130.7, 127.7 (2C), 126.6, 124.3, 124.2, 108.1, 79.5, 71.9, 60.4, 52.1, 47.8, 45.6, 45.4, 45.3, 40.5, 38.8, 30.1, 29.7, 28.3 (2C), 28.0, 27.9, 26.5, 25.6, 22.3, 17.9 ppm. *Note*: The *C*=O carbons of the Dde protecting group and the *C*-*C*F_3 carbons of the substituted diazirine ring are not observed in the ^{13}C-NMR spectrum.

3.3.6.5 Removal of the Dde group from intermediate **45** – Preparation of intermediate **46**

A solution $H_2NNH_2 \cdot H_2O$ (60 μL, 1.3 mmol) in DMF (3 mL) was added to intermediate **45** (0.24 g, 0.23 mmol), and the reaction mixture was stirred at ambient temperature under Ar for 1 h. Additional $H_2NNH_2 \cdot H_2O$ (30 μL) and after 30 min the completion of the reaction was verified by TLC with solvent R as eluent. The reaction mixture was then diluted with DCM and the organic phase was washed twice with H_2O and once with brine, dried over anhydrous Na_2SO_4, filtered and evaporated to dryness under reduced pressure. The residue was subjected to FCC using the solvent system F for elution yielding pure partially deprotected intermediate **46** ($C_{36}H_{40}F_3N_9O_{10}S_2$, exact mass: 879.23). Slightly yellow oil (0.15 g, 73%); R_f (F): 0.23; MS (ESI, 30 eV): *m/z* 880.30 $[M+H]^+$.

3.3.6.6 Tritylation of partially deprotected intermediate **46** – Preparation of intermediate **47**

To a solution of compound **46** (0.14 g, 0.16 mmol) in dry DCM (0.7 mL) was added sequentially dry Et_3N (40 μL, 0.29 mmol) and TrtCl (50 mg, 0.18 mmol) at ambient temperature under Ar and the resulting reaction mixture was stirred for 1 h. The completion of the reaction was verified by TLC with solvent system F as eluent. The reaction mixture was then diluted with DCM and the organic phase was washed once with 5% aqueous $NaHCO_3$ and twice with H_2O, dried over anhydrous Na_2SO_4, filtered and evaporated to dryness under reduced pressure to leave crude intermediate **47** as a yellowish oil which was used without further purification to the next denosylation experiment.

3.3.6.7 Removal of the Ns groups from intermediate **47** – Preparation of the N-tritylated conjugate **48**

To a solution of intermediate **47** (0.16 mmol) in DMF (2.0 mL), Na_2CO_3 (0.14 g, 1.3 mmol) and PhSH (50 μL, 0.48 mmol) were sequentially added at ambient temperature under Ar. The reaction mixture was vigorously stirred for 2 h and then additional PhSH (50 μL) was added. Stirring was continued for 2 h and then additional Na_2CO_3 (70 mg) and PhSH (70 μL) were added. After 1 h, the completion of the reaction was verified by TLC using solvent R as eluent. The reaction mixture was diluted with H_2O and the aqueous phase was extracted twice with DCM. The combined aqueous layers were washed twice with H_2O and once with brine, dried over anhydrous Na_2SO_4, filtered and evaporated under reduced pressure. The residue was subjected to FCC using the solvent system F to yield pure intermediate **48** ($C_{43}H_{48}F_3N_7O_2$, exact mass: 751. 38). Slightly yellow oil; (75 mg, 75% for the two steps); R_f (F): 0.23; ^{1}H-NMR (600 MHz, $CDCl_3$): δ 7.85 (dt, J = 8.4 and 0.6 Hz, 2H), 7.47-7.43 (m, 6H), 7.34 (t, J = 4.3 Hz, 1H), 7.30 (d, J = 7.2 Hz, 1H), 7.28-7.23 (m, 8H), 7.19- 7.15 (m, 3H), 4.67 (td, J = 7.2 and 6.0 Hz, 1H), 3.36-3.23 (m, 3H), 2.86 (ddd, J = 16.8, 5.4 and 2.4 Hz, 1H), 2.74-2.65 (m, 8H), 2.60 (t, J = 6.0 Hz, 2H), 2.19 (t, J = 6.6 Hz, 2H), 2.09 (t, J = 3.0 Hz, 1H), 1.72-1.65 (m, 5H), 1.62-1.56 (m, 2H), 1.56-1.49 (m, 2H) ppm. ^{13}C{^{1}H}-NMR (150 MHz, $CDCl_3$): δ 169.4, 165.8, 146.2 (3C), 134.7, 132.7, 128.6 (6C), 127.8 (6C), 127.7 (2C), 126.6 (2), 126.2 (3C), 79.6, 71.8, 70.9, 52.1, 49.1, 48.7 (2C), 48.6, 48.4, 42.0, 39.6, 30.8, 27.3, 27.2, 22.7 ppm; MS (ESI, 30 eV): *m/z* 752.48 $[M+H]^+$. *Note*: The *C*-*C*F_3 carbons of the substituted diazirine ring are not observed in the ^{13}C-NMR spectrum.

3.3.6.8 Removal of the Trt group from intermediate **48** – PAT analog **9**

A cold (0 °C) solution consisted of TFA (0.5 mL) and DCM (4.5 mL) was added to the intermediate **48** (75 mg, 0.1 mmol) and the resulting reaction mixture was kept at ambient temperature for 30 min whereby the completion of the reaction was verified with TLC using solvent system G as eluent. Volatiles were then evaporated under reduced pressure to leave a residue which upon adding dry diethyl ether and overnight refrigeration gave a precipitate. The solvent was decanted and fresh dry diethyl ether was added to wash the solid. The solvent was decanted again and the remaining solid was dried over P_2O_5 overnight, under vacuo, yielding pure PAT analog **9** ($C_{24}H_{34}F_3N_7O_2$, exact mass: 509. 27) as the corresponding tritrifluoroacetate salt. White solid (55 mg, 65%); RP-HPLC: t_R = 4.742 min; ^{1}H-NMR (600 MHz, D_2O): δ 7.96 (dt, J = 8.4 and 0.6 Hz, 2H), 7.36 (d, J = 8.4 Hz, 2H), 7.30-7.25 (m, 1H), 7.25-7.20 (m, 1H) 4.61 (dd, J = 7.8 and 6.6 Hz, 1H), 3.32-3.29 (m, 4H), 3.27 (td, J = 6.6, and 4.2 Hz, 2H), 3.17-3.1 (m, 6H), 3.05 (q, J = 7.8 Hz, 4H), 2.81 (ddd, J = 16.8, 6.0 and 2.4 Hz, 1H), 2.73 (ddd, J = 16.8, 7.8 and 2.4 Hz, 1H), 2.42 (t, J = 3.0 Hz, 1H), 2.16-2.06 (m, 4H), 1.76-1.69 (m, 2H), 1.61 (q, J = 7.2 Hz, 2H) ppm. ^{13}C{^{1}H}-NMR (150 MHz, D_2O): δ 171.3, 167.6, 135.2, 132.0, 128.0, 127.9, 127.2, 126.3, 122.0 (q, J = 274.3 Hz), 79.0, 71.0, 53.4, 44.6 (2C) , 44.3, 38.1, 36.4, 28.0 (q, J = 40.2 Hz), 25.9, 24.0, 22.9, 22.7, 21.1 ppm; MS (ESI, 30 eV): *m/z* 510.32 $[M+H]^+$. Anal. Calcd (%) for $C_{30}H_{37}F_{12}N_7O_8$: C, 42.31; H, 4.38; N, 11.51. Found: C, 42.59; H, 4.15; N, 11.32.

3.3.7. PAT analog **10**

3.3.7.1 Removal of the Dde group from the Tsm derivative **17** – Preparation of intermediate **42**

To a solution of orthogonally protected Tsm **17** (0.50 g, 0.51 mmol) in DMF (6.5 mL), $H_2NNH_2 \cdot H_2O$ (0.14 mL, 2.89 mmol) was added and the reaction mixture was stirred at ambient temperature under Ar for 30 min whereby completion of the reaction was verified by TLC with solvent R as eluent. The reaction mixture was then diluted with diethyl ether and the organic phase was washed thrice with H_2O and once with brine, dried over anhydrous Na_2SO_4, filtered and evaporated to dryness under reduced pressure. The residue was subjected to FCC using the solvent system E for elution yielding pure partially protected Tsm **42**. Slightly yellow oil (0.33 g, 79%); R_f(E): 0.11.

3.3.7.2 Coupling of amine **42** with Fmoc-(L)-propargylglycine (**27**) – Synthesis of amide **49**

To a cold (0 °C) solution of amine **42** (0.29 g, 0.36 mmol) and dry DIPEA (0.19 mL, 1.08 mmol) in anhydrous DMF (1 mL), the amino acid derivative **27** (0.14 g, 0.42 mmol) and PyBrOP (0.23 g, 0.5 mmol) were added sequentially under Ar. The reaction mixture was then stirred at ambient temperature for 30 min whereby completion of the reaction was certified with TLC using E as eluent. The reaction mixture was then diluted with ethyl acetate and the organic phase was washed sequentially once with 5% aqueous $NaHCO_3$, twice with H_2O and once with brine. Drying over anhydrous Na_2SO_4, filtration and evaporation of the solvent under reduced pressure left a residue, which was subjected to FCC using the solvent system N for elution yielding pure amide **49**. Slightly yellow oil (0.31 g, 76%); R_f(N): 0.15; ^{1}H-NMR (600 MHz, $CDCl_3$): δ 7.99-7.92 (m, 2H), 7.79-7.72 (m, 2H), 7.67-7.56 (m, 7H), 7.54-7.50 (m, 1H), 7.45-7.40 (m, 6H), 7.40-7.36 (m, 2H), 7.29-7.26 (m, 7H), 7.20-7.18 (m, 4H), 6.72 (unresolv. t, 1H), 5.67 (d, J = 6.6 Hz, 1H), 4.43-4.32 (m, 3H), 4.22 (t, J = 6.6 Hz, 1H), 3.41-3.30 (m, 4H), 3.29-3.18 (m, 6H), 2.88-2.79 (m, 1H), 2.71-2.61 (m, 1H), 2.14 (t, J = 3.0 Hz, 1H), 2.05 (t, J = 7.2 Hz, 2H), 1.87-1.72 (m, 4H), 1.50 (q, J = 7.2 Hz, 2H), 1.38 (q, J = 7.8 Hz, 2H), 1.26 (br. s, 1H) ppm. $^{13}C\{^1H\}$-NMR (150 MHz, $CDCl_3$): δ 170.2, 146.5 (3), 144.2, 144.1, 141.7, 138.3, 134.1, 134.0, 133.6, 132.3, 132.3, 132.1, 131.2, 131.1, 129.4 (4C), 129.0 (2C), 128.6 (6C), 128.2 (6C), 128.1, 127.5, 126.7 (3C), 125.7 (2C), 124.6, 124.5, 120.4, 71.2, 67.8, 48.2, 47.5, 46.0, 45.9, 45.3, 43.5, 36.6, 36.5, 28.2, 28.0, 26.3 ppm.

3.3.7.3 Removal of the Fmoc group from amide **49** – Preparation of intermediate **50**

A solution of Et_2NH (1.5 mL, 14.50 mmol) in DCM (6 mL) was added to amide **49** (0.31 g, 0.27 mmol) under Ar and the resulting reaction mixture was kept at ambient temperature for 1 h whereby the completion of the reaction was certified using TLC with eluent R. It was then diluted with additional DCM and the organic phase was washed thrice with H_2O, dried and evaporated under reduced pressure to dryness. The resulting crude amine **50** was used as such into the next experiment.

3.3.7.4 Coupling of amine **50** with 'active' ester **29** – Synthesis of bisamide **51**

To a solution of amine **50** (0.27 mmol) in dry DMF (0.5 mL), DIPEA (60 μL, 0.34 mmol) and the 'active' ester **29** (88 mg, 0.27 mmol) were added sequentially at ambient temperature under Ar. The reaction mixture was stirred overnight at ambient temperature whereby the completion of the reaction was certified with TLC using the

solvent system A. It was then applied directly on the top of a chromatography column and subjected to FCC using the solvent system O for elution yielding pure bisamide **51**. Slightly yellow oil (0.22 g, 74%); R_f (O): 0.19.

3.3.7.5 Removal of the Ns groups from intermediate **51** – Preparation of the N-tritylated conjugate **52**

To a solution of intermediate **51** (0.22 g, 0.2 mmol) in DMF (2.5 mL), Na_2CO_3 (0.17 g, 1.6 mmol) and PhSH (60 µL, 0.6 mmol) were sequentially added at ambient temperature under Ar. The reaction mixture was vigorously stirred for 3 h whereby completion of the reaction was verified by TLC using solvent R as eluent. The reaction mixture was diluted with H_2O and the aqueous phase was extracted twice with DCM. The combined aqueous layers were washed twice with H_2O and once with brine, dried over anhydrous Na_2SO_4, filtered and evaporated under reduced pressure. The residue was subjected to FCC using the solvent system F to yield pure intermediate **52** ($C_{43}H_{48}F_3N_7O_2$, exact mass: 751. 88). Slightly yellow oil (0.14 g, 92%); R_f (G): 0.40; ^{1}H-NMR (600 MHz, $CDCl_3$): δ 7.85 (d, J = 8.4 Hz, 2H), 7.71 (unresolv. t, 1H), 7.48-7.43 (m, 6H), 7.35 (d, J = 6.0 Hz, 1H), 7.28-7.23 (m, 8H), 7.19-7.15 (m, 3H), 4.66 (q, J = 6.0 Hz, 1H), 3.45-3.36 (m, 2H), 2.87-2.80 (m, 1H), 2.78-2.69 (m, 3H), 2.67-2.62 (m, 3H), 2.59-2.55 (m, 2H), 2.13 (unresolv. t, 2H), 2.07 (unresolv. t, 1H), 2.02-1.81 (m, 5H), 1.69 (q, J = 6.0 Hz, 2H), 1.64 (q, J = 6.0 Hz, 2H), 1.26 (br. s, 1H) ppm. $^{13}C\{^1H\}$-NMR (150 MHz, $CDCl_3$): δ 172.4, 172.1, 146.3 (3C), 134.8, 132.7, 128.7 (6C), 127.8 (6C), 127.7 (2C), 126.6 (2C), 126.2 (3C), 79.4, 71.7, 70.9, 52.1, 50.0, 48.6, 48.5, 48.1, 43.4, 39.2, 29.6, 28.6, 28.4, 27.7, 22.7 ppm; MS (ESI, 30 eV): *m/z* 752.22 $[M+H]^+$, 243.11 $[Trt]^+$.

Note: The *C*-*C*F_3 carbons of the substituted diazirine ring are not observed in the ^{13}C-NMR spectrum.

3.3.7.6 Removal of the Trt group from intermediate **52** – PAT analog **10**

A cold (0 °C) solution consisted of TFA (1 mL) and DCM (9 mL) was added to the intermediate **52** (0.14 g, 0.19 mmol) and the resulting reaction mixture was kept at ambient temperature for 1 h whereby the completion of the reaction was verified with TLC using G as eluent. Volatiles were then evaporated under reduced pressure to leave a residue which upon adding dry diethyl ether and overnight refrigeration gave a precipitate. The solvent was decanted and fresh dry diethyl ether was added to wash the solid. The solvent was decanted again and the remaining solid was dried over P_2O_5 overnight, under vacuo, yielding pure PAT analog **10** ($C_{24}H_{34}F_3N_7O_2$, exact mass: 509.

27) as the corresponding tritrifluoroacetate salt. White solid (0.14 g, 87%); RP-HPLC: t_R = 4.738 min; ^{1}H-NMR (600 MHz, CD_3OD): δ 7.97 (unresolv. dt, J = 8.4, 2H), 7.36 (d, J = 7.8 Hz, 2H), 7.30-7.25 (m, 1H), 7.24-7.21 (m, 1H), 4.58-4.53 (m, 1H), 3.43-3.33 (m, 2H), 3.16-3.10 (m, 4H), 3.10-3.04 (m, 4H), 2.97 (t, J = 7.2 Hz, 2H), 2.86-2.75 (m, 2H), 2.46 (t, J = 1.7 Hz, 1H), 2.17-2.09 (m, 2H), 1.91 (q, J = 6.6 Hz, 2H), 1.83-1.70 (m, 4H) ppm. $^{13}C\{^1H\}$-NMR (150 MHz, D_2O): δ 172.1, 167.8, 135.0, 132.1, 128.0, 127.9, 127.2, 126.3, 122.0 (q, J = 273.0 Hz), 79.0, 71.3, 53.6, 45.0, 44.5, 44.4, 38.6, 35.5, 28.0 (q, J = 40.5 Hz), 26.1, 24.1, 22.8, 22.7, 20.8 ppm; MS (ESI, 30 eV): *m/z* 510.16 $[M+H]^+$. Anal. Calcd (%) for $C_{30}H_{37}F_{12}N_7O_8$: C, 42.31; H, 4.38; N, 11.51. Found: C, 42.56; H, 4.18; N, 11.30.

3.3.8. PAT analog **6**

3.3.8.1 Coupling of amine **53** with Fmoc-(L)-Tyr(^{t}Bu)-OH (**26**) – Synthesis of amide **58**

To a cold (0 °C) solution of amine **53** (0.33 g, 0.40 mmol) and dry DIPEA (0.15 mL, 0.88 mmol) in anhydrous DMF (1 mL), the amino acid derivative **26** (0.20 g, 0.44 mmol) and HBTU (0.18 g, 0.48 mmol) were added sequentially under Ar. The reaction mixture was then stirred at ambient temperature for 30 min whereby completion of the reaction was certified with TLC using F as eluent. The reaction mixture was then diluted with ethyl acetate and the organic phase was washed sequentially once with 5% aqueous $NaHCO_3$, twice with H_2O and once with brine. Drying over anhydrous Na_2SO_4, filtration and evaporation of the solvent under reduced pressure left crude amide **58** ($C_{69}H_{73}N_7O_{12}S_2$, exact mass: 1255.48) which was used as such into the next experiment, without further purification. Slightly yellow oil; R_f (N): 0.22; MS (ESI, 30 eV): *m/z* 1294.60 $[M+K]^+$, 1278.55 $[M+Na]^+$, 1256.32 $[M+H]^+$, 243.81 $[Trt]^+$.

3.3.8.2 Removal of the Fmoc group from compound **58** – Preparation of intermediate **59**

A solution made up of Et_2NH (2 mL) and DCM (6 mL) was added to compound **58** (0.40 mmol) under Ar and the resulting reaction mixture was stirred at ambient temperature for 1 h. The completion of the reaction was verified with TLC using the solvent system N as eluent. The reaction mixture was diluted with DCM and the organic phase was washed thrice with H_2O. Drying over anhydrous Na_2SO_4 followed by filtration and evaporation of the solvent under reduced pressure left crude amine **59** ($C_{54}H_{63}N_7O_{12}S_2$, exact mass: 1033.41), which was used as such into the next experiment

without further purification. Colorless oil; R_f (N): 0.02; MS (ESI, 30 eV): *m/z* 1072.05 $[M+K]^+$, 1056.29 $[M+Na]^+$, 243.88 $[Trt]^+$.

3.3.8.3 Coupling of amine **59** with butanoic acid – Synthesis of bisamide **60**

To a cooled (0 °C) solution of amine **59** (0.4 mmol) and DIPEA (0.14 mL, 0.8 mmol) in DMF (1 mL), butanoic acid (40 μL, 0.44 mmol) was added followed by the coupling agent HBTU (0.18 g, 0.48 mmol). The reaction mixture was allowed to attain ambient temperature were it was stirred for 30 min. The completion of the reaction was verified by TLC with the solvent system E. The reaction mixture was then diluted with ethyl acetate and the organic phase was washed once with 5% aqueous $NaHCO_3$, twice with H_2O and once with brine. It was finally dried over anhydrous Na_2SO_4, filtered and evaporated to dryness. The residue was then subjected to FCC using the eluent Q to give pure bisamide **60** ($C_{58}H_{69}N_7O_{11}S_2$, exact mass: 1103.45).

Slightly yellow oil (0.28 g, 64% for the three steps); R_f (EtOAc/Tol (7:3): 0.25; MS (ESI, 30 eV): *m/z* 1126.75 $[M+Na]^+$, 1104.44 $[M+H]^+$, 243.59 $[Trt]^+$.

3.3.8.4 Removal of the Ns groups from bisamide **60** – Preparation of the N-tritylated conjugate **61**

To a solution of intermediate **60** (0.28 g, 0.25 mmol) in DMF (2.5 mL), Na_2CO_3 (0.26 g, 2.5 mmol) and PhSH (0.2 mL, 2.0 mmol) were sequentially added at ambient temperature under Ar. The reaction mixture was vigorously stirred for 3 h whereby completion of the reaction was verified by TLC using F as eluent. The reaction mixture was diluted with H_2O and the aqueous phase was extracted twice with DCM. The combined aqueous layers were washed twice with H_2O and once with brine, dried over anhydrous Na_2SO_4, filtered and evaporated under reduced pressure. The residue was subjected to FCC using the solvent system G to yield pure intermediate **61** ($C_{46}H_{63}N_5O_3$, exact mass: 733.49). Slightly yellow oil (0.12 g, 65%); R_f (G): 0.31; ^{1}H-NMR (600 MHz, $CDCl_3$): δ 7.48-7.43 (m, 6H), 7.28-7.25 (m, 6H), 7.20-7.15 (m, 3H), 7.10 (d, J = 8.4 Hz, 2H), 6.89 (d, J = 8.4 Hz, 2H), 6.32 (d, J = 7.8 Hz, 1H), 4.50 (q, J = 7.2 Hz, 1H), 3.24 (q, J = 6.0 Hz, 2H), 3.01 (dd, J = 13.8 and 6.6 Hz, 1H), 2.94 (two dd, J = 13.8 and 6.6 Hz, 1H), 2.70 (t, J = 7.2 Hz, 2H), 2.63 (t, J = 6.6 Hz, 2H), 2.57-2.48 (m, 4H), 2.19 (t, J = 6.6 Hz, 2H), 2.14 (td, J = 7.2 and 3.6 Hz, 2H), 1.69 (quint., J = 7.2 Hz, 2H), 1.62-1.55 (m, 4H), 1.54-1.44 (m, 6H), 1.31 (s, 9H), 1.25 (br. s, 1H), 0.87 (t, J = 7.2 Hz, 3H) ppm. $^{13}C\{^1H\}$-NMR (150 MHz, $CDCl_3$): δ 146.1 (3C), 131.8, 131.2, 129.8 (2C), 128.6 (6C), 127.8 (6C), 126.2 (3C), 124.2 (2C), 92.0, 70.9, 54.9 (2), 49.5, 48.5, 48.2, 42.0, 38.5, 38.2, 28.8 (3C), 28.1, 27.8, 27.7, 19.0 (2C), 13.7 (2C) ppm; MS

(ESI, 30 eV): *m/z* 772.70 [M+K]$^+$, 756.57 [M+Na]$^+$, 734.76 [M+H]$^+$, 243.67[Trt]$^+$. *Note*: The two *C*=O carbons of the amide groups are not observed in the ^{13}C-NMR spectrum.

3.3.8.5 Deprotection of intermediate **61** – PAT analog **6**

A cold (0 °C) solution of TFA (0.8 mL) and PhSH (80 μL, 0.80 mmol) in DCM (0.8 mL) was added to the intermediate **61** (0.12 g, 0.16 mmol) and the resulting solution was kept at ambient temperature for 1, whereby completion of deprotection was verified by TLC using the solvent system I as eluent. Evaporation under reduced pressure left a residue which upon addition of diethyl ether and overnight refrigeration yielded a white precipitate. The supernatant liquor was decanted, fresh diethyl ether was added to wash the precipitate, the supernatant liquor was again decanted, and the solid residue was dried overnight, under reduced pressure over KOH pellets, to yield pure product **6** ($C_{23}H_{41}N_5O_3$, exact mass: 435.32) as the corresponding tritrifluoroacetate salt. White solid (0.11 g, 90%); RP-HPLC: t_R = 2.505 min; MS (ESI, 30 eV): *m/z* 458.66 [M+Na]$^+$, 436.78 [M+H]$^+$. Anal. Calcd (%) for $C_{29}H_{44}F_9N_5O_9$: C, 44.79; H, 5.70; N, 9.01. Found: C, 45.01; H, 5.48; N, 8.79.

3.3.9. PAT analog **7**

3.3.9.1 Removal of the Phth group from compound **18** – Preparation of *N*^{5},*N*^{10}-dinosyl-*N*^{1}-tritylhomospermine (**62**)

To a solution of Hsm derivative **18** (0.29 g, 0.6 mmol) in ethanol (4 mL), $H_2NNH_2 \cdot H_2O$ (60 μL, 1.2 mmol) was added and the reaction mixture was refluxed for 1 h. Additional $H_2NNH_2 \cdot H_2O$ (60 μL) was added and the reaction mixture was further refluxed for 1 h, whereby completion of the reaction was verified with TLC using the system L as eluent. The solvent was evaporated under reduced pressure, 5% aqueous $NaHCO_3$ was added to the residue and the aqueous phase was extracted twice with DCM. The combined organic phases were washed once with brine, dried over anhydrous Na_2SO_4, filtered and evaporated to dryness. The residue was subjected to FCC using the solvent system F as eluent to yield pure product **62** ($C_{43}H_{50}N_6O_8S_2$, exact mass: 842.31). Slightly yellow oil (0.21 g, 41%); R_f (F): 0.18; MS (ESI, 30 eV): *m/z* 881.45 [M+K]+, 843.38 [M+H]$^+$, 243.38 [Trt]$^+$.

3.3.9.2 Coupling of amine **62** with Fmoc-(L)-Tyr(^{t}Bu)-OH (**26**) – Synthesis of amide **63**

To a cold (0 °C) solution of amine **62** (0.21 g, 0.25 mmol) and dry DIPEA (90 μL, 0.52 mmol) in anhydrous DMF (0.7 mL), the amino acid derivative **26** (0.13 g, 0.28 mmol) and HBTU (0.12 g, 0.32 mmol) were added sequentially under Ar. The reaction mixture was then stirred at ambient temperature for 30 min whereby completion of the reaction was certified with TLC using F as eluent. The reaction mixture was then diluted with ethyl acetate and the organic phase was washed sequentially once with 5% aqueous $NaHCO_3$, twice with H_2O and once with brine. Drying over anhydrous Na_2SO_4, filtration and evaporation of the solvent under reduced pressure left an oily residue which was subjected to FCC using the solvent system O for elution providing pure amide **63** ($C_{71}H_{77}N_7O_{12}S_2$, exact mass: 1283.51). Slightly yellow oil (0.23 g, 73%); R_f (O): 0.34; MS (ESI, 30 eV): *m/z* 1306.48 $[M+Na]^+$, 1284.24 $[M+H]^+$, 243.31 $[Trt]^+$.

3.3.9.3 Removal of the Fmoc group from compound **63** – Preparation of intermediate **64**

A solution made up of Et_2NH (1.5 mL) and DCM (4.5 mL) was added to compound **63** (0.23 g, 0.18 mmol) under Ar and the resulting reaction mixture was stirred at ambient temperature for 1 h. The completion of the reaction was verified with TLC using the solvent system O as eluent. The reaction mixture was diluted with DCM and the organic phase was washed thrice with H_2O. Drying over anhydrous Na_2SO_4 followed by filtration and evaporation of the solvent under reduced pressure left crude amine **64**, which was used as such into the next experiment without further purification. Yellow oil; R_f (E): 0.60.

3.3.9.4 Coupling of amine **64** and butanoic acid – Synthesis of bisamide **65**

To a cooled (0 °C) solution of amine **64** (0.18 mmol) and DIPEA (60 μL, 0.34 mmol) in DMF (0.7 mL), butanoic acid (20 μL, 0.22 mmol) was added followed by the coupling agent HBTU (0.1 g, 0.26 mmol). The reaction mixture was allowed to attain ambient temperature were it was stirred for 30 min. The completion of the reaction was verified by TLC with the solvent system E. The reaction mixture was then diluted with ethyl acetate and the organic phase was washed once with 5% aqueous $NaHCO_3$, twice with H_2O and once with brine. It was finally dried over anhydrous Na_2SO_4, filtered and evaporated to dryness. The residue was then subjected to FCC using initially the eluent P and the eluent R to give pure bisamide **65** ($C_{60}H_{73}N_7O_{11}S_2$, exact mass: 1131.48). Yellow oil; R_f (R): 0.33; MS (ESI, 30 eV): *m/z* 1132.52 $[M+H]^+$.

3.3.9.5 Removal of the Ns groups from bisamide **65** – Preparation of the partially protected conjugate **66**

To a solution of intermediate **65** (0.18 mmol) in DMF (1.8 mL), Na_2CO_3 (0.15 g, 1.43 mmol) and PhSH (0.14 mL, 1.4 mmol) were sequentially added at ambient temperature under Ar. The reaction mixture was vigorously stirred for 3 h whereby completion of the reaction was verified by TLC using solvent R as eluent. The reaction mixture was diluted with H_2O and the aqueous phase was extracted twice with DCM. The combined aqueous layers were washed twice with H_2O and once with brine, dried over anhydrous Na_2SO_4, filtered and evaporated under reduced pressure. The residue was subjected to FCC using initially the solvent system F, then G and finally the solvent system H to yield pure intermediate **66** ($C_{48}H_{67}N_5O_5$, exact mass: 761.52). Slightly yellow oil (0.11 g, 79%); R_f (F): 0.14; ^{1}H NMR (600 MHz, $CDCl_3$): δ 7.46 (d, J = 7.2 Hz, 6H), 7.25 (t, , J = 7.2 Hz, 6H), 7.17 (t, , J = 7.2 Hz, 3H), 7.10 (d, J = 8.4 Hz, 2H), 6.89 (d, J = 8.4 Hz, 2H), 6.76 (unresolv. t, 1H), 6.35 (unresolv. d, 1H), 4.53 (q, J = 7.2 Hz, 1H), 3.18-3.09 (m, 2H), 3.00 and 2.96 (two dd, J = 13.8 and 7.2 Hz, 2H), 2.61 (t, J = 6.0 Hz, 2H), 2.57 (t, J = 6.6 Hz, 4H), 2.55-2.47 (m, 2H), 2.15-2.11 (m, 4H), 1.60-1.49 (m, 10H), 1.44-1.38 (m, 4H), 1.31 (s, 9H), 1.25 (s, 3H), 0.86 (t, J = 7.2 Hz 3H) ppm. ^{13}C{^{1}H} NMR (150 MHz, $CDCl_3$): δ 172.8, 170.9, 154.2, 146.2 (3C), 131.9, 129.8 (2C), 128.6 (6C), 127.7 (6C), 126.2 (3C), 124.2 (2C), 78.4, 70.8, 54.8, 49.5, 48.9, 43.4, 39.1, 38.5, 38.1, 29.7, 28.8 (3C), 28.6, 27.7, 27.6, 27.1, 27.0, 19.0 (2C), 13.6 (2C) ppm; MS (ESI, 30 eV): *m/z* 762.48 $[M+H]^+$.

3.3.9.6 Deprotection of intermediate **66** – PAT analog **7**

A cold (0 °C) solution of TFA (1.7 mL) and PhSH (0.34 mL, 3.3 mmol) in DCM (1.7 mL) was added to the intermediate **66** (0.11 g, 0.14 mmol) and the resulting solution was kept at ambient temperature for 1, whereby completion of deprotection was verified by TLC using the solvent system I as eluent. Evaporation under reduced pressure left a residue which upon addition of diethyl ether and overnight refrigeration yielded a white precipitate. The supernatant liquor was decanted, fresh diethyl ether was added to wash the precipitate, the supernatant liquor was again decanted and the solid residue was dried overnight, under reduced pressure over KOH pellets, to yield pure product **7** ($C_{25}H_{45}N_5O_3$, exact mass: 463.35) as the corresponding tritrifluoroacetate salt. Slightly yellow oil (96 mg, 85%); RP-HPLC: t_R = 2.546 min; MS (ESI, 30 eV): *m/z* 486.95 $[M+Na]^+$, 464.72 $[M+H]^+$.

3.3.10. PAT analog **8**

3.3.10.1 Alkylation of N^4,N^8,N^{11}-trinosyl-N^1-tritylnorspermine (**20**) with $PhthN(CH_2)_4Br$ – Synthesis of penta-amine derivative **67**

To a solution of Nsm derivative **20** (0.39 g, 0.40 mmol) and *N*-(4-bromobutyl)phthalimide (0.12 g, 0.44 mmol) in anhydrous DMF (0.6 mL), K_2CO_3 (0.12 g, 0.90 mmol) was added under Ar at ambient temperature. The reaction mixture was vigorously stirred at 60 °C for 2.5 h. Completion of the reaction was verified with TLC using the solvent system E for elution. It was then diluted with H_2O and extracted with ethyl acetate. The organic phase was washed twice with H_2O and once with brine, dried over anhydrous Na_2SO_4, filtered and the filtrate evaporated under reduced pressure to leave crude penta-amine derivative **67** which was used as such into the next experiment. Yellow oil; R_f (E): 0.69.

3.3.10.2 Removal of the Phth group from compound **67** – Preparation of the 4-3-3-3 PA intermediate **68**

To a solution of PA derivative **67** (0.40 mmol) in ethanol (3 mL), $H_2NNH_2{\cdot}H_2O$ (40 μL, 0.8 mmol) was added and the reaction mixture was refluxed for 1 h. Additional $H_2NNH_2{\cdot}H_2O$ (40 μL) was added and the reaction mixture was further refluxed for 1 h, whereby completion of the reaction was verified with TLC using the system E as eluent. The solvent was evaporated under reduced pressure, 5% aqueous $NaHCO_3$ was added to the residue and the aqueous phase was extracted twice with DCM. The combined organic phases were washed once with brine, dried over anhydrous Na_2SO_4, filtered and evaporated to dryness. The residue was subjected to FCC using the solvent system G as eluent to yield pure product **68** ($C_{50}H_{56}N_8O_{12}S_3$, exact mass: 1056.32). Yellow oil (0.22 g, 52%); R_f (G): 0.28; MS (ESI, 30 eV): *m/z* 1079.17 $[M+Na]^+$, 243.38 $[Trt]^+$.

3.3.10.3 Coupling of amine **68** with Fmoc-(L)-Tyr(^{t}Bu)-OH (**26**) – Synthesis of amide **69**

To a cold (0 °C) solution of amine **68** (0.22 g, 0.21 mmol) and dry DIPEA (80 μL, 0.46 mmol) in anhydrous DMF (0.6 mL), the amino acid derivative **26** (0.11 g, 0.23 mmol) and HBTU (0.10 g, 0.27 mmol) were added sequentially under Ar. The reaction mixture was then stirred at ambient temperature for 30 min whereby completion of the reaction was certified with TLC using solvent system G as eluent. The reaction mixture was then diluted with ethyl acetate and the organic phase was washed sequentially once with 5% aqueous $NaHCO_3$, twice with H_2O and once with brine. Drying over anhydrous

Na_2SO_4, filtration and evaporation of the solvent under reduced pressure left an oily residue which was subjected to FCC using the solvent system O for elution providing pure amide **69** ($C_{78}H_{83}N_9O_{16}S_3$, exact mass: 1497.51). Slightly yellow oil (0.19 g, 60%); R_f (O): 0.24; MS (ESI, 30 eV): *m/z* 1537. 02 $[M+K]^+$, 1520.90 $[M+Na]^+$, 1498.22 $[M+H]^+$, 243.88 $[Trt]^+$.

3.3.10.4 Removal of the Fmoc group from compound **69** - Preparation of intermediate **70**

A solution made up of Et_2NH (3.5 mL) and DCM (10.5 mL) was added to compound **69** (0.19 g, 0.13 mmol) under Ar and the resulting reaction mixture was stirred at ambient temperature for 1 h. The completion of the reaction was verified with TLC using the solvent system O as eluent. The reaction mixture was diluted with DCM and the organic phase was washed thrice with H_2O. Drying over anhydrous Na_2SO_4 followed by filtration and evaporation of the solvent under reduced pressure left crude amine **70**, which was used as such into the next experiment without further purification. Yellow oil; R_f (E): 0.52.

3.3.10.5 Coupling of amine **70** and butanoic acid – Synthesis of bisamide **71**

To a cooled (0 °C) solution of amine **70** (0.13 mmol) and DIPEA (45 μL, 0.26 mmol) in DMF (0.5 mL), butanoic acid (15 μL, 0.17 mmol) was added followed by the coupling agent HBTU (64 mg, 0.17 mmol). The reaction mixture was allowed to attain ambient temperature were it was stirred for 30 min. The completion of the reaction was verified by TLC with the solvent system D. The reaction mixture was then diluted with ethyl acetate and the organic phase was washed once with 5% aqueous $NaHCO_3$, twice with H_2O and once with brine. It was finally dried over anhydrous Na_2SO_4, filtered and evaporated to dryness. The residue was then subjected to FCC using initially the eluent P and then eluent R to give bisamide **71** ($C_{67}H_{79}N_9O_{15}S_3$, exact mass: 1345.49) which was used as such into the next denosylation experiment. Orange oil; R_f (P): 0.20; MS (ESI, 30 eV): *m/z* 1368.86 $[M+Na]^+$, 1346.89 $[M+H]^+$.

3.3.10.6. Removal of the Ns groups from bisamide **71** – Preparation of the partially protected conjugate **72**

To a solution of intermediate **71** (0.13 mmol) in DMF (1.3 mL), Na_2CO_3 (0.22 g, 2.08 mmol) and PhSH (0.16 mL, 1.56 mmol) were sequentially added at ambient temperature under Ar. The reaction mixture was vigorously stirred overnight whereby completion of the reaction was verified by TLC using solvent R as eluent. The reaction mixture was diluted with H_2O and the aqueous phase was extracted twice with DCM.

The combined aqueous layers were washed twice with H_2O and once with brine, dried over anhydrous Na_2SO_4, filtered and evaporated under reduced pressure. The residue was subjected to FCC using initially the solvent system G, then H and finally the solvent system I to yield pure intermediate **72** ($C_{49}H_{70}N_6O_3$, exact mass: 790.55). Slightly yellow oil (76 mg, 74%); R_f (G): 0.16; ^{1}H NMR (600 MHz, $CDCl_3$): δ 7.45 (d, J = 7.2 Hz, 6H), 7.25 (t, J = 7.2 Hz, 6H), 7.16 (t, J = 7.2 Hz, 3H), 7.10 (d, J = 8.4 Hz, 2H), 6.88 (d, J = 8.4 Hz, 2H), 6.86 (unresolv. t, 1H), 6.41 (d, J = 7.2 Hz, 1H), 4.54 (q, J = 7.2 Hz, 1H), 3.13 (q, J = 4.8 Hz, 2H), 3.00-2.95 (m, 2H), 2.69-2.61 (m, 10H), 2.56-2.52 and 2.52-2.48 (two m, 2H), 2.17 (t, J = 6.6 Hz, 2H), 2.12 (t, J = 6.6 Hz, 2H), 1.70-1.63 (m, 6H), 1.57 (sextet, J = 7.2 Hz, 2H), 1.43-1.37 (m, 4H), 1.30 (s, 9H), 1.25 (br. s, 4H), 0.86 (t, J = 7.2 Hz 3H) ppm. $^{13}C\{^1H\}$ NMR (150 MHz, $CDCl_3$): δ 172.8, 170.9, 154.2, 146.1 (3C), 131.8, 129.8 (2C), 128.6 (6C), 127.7 (6C), 126.2 (3C), 124.2 (2C), 78.3, 70.9, 54.7, 49.2, 48.6, 48.4, 42.0, 39.2, 38.4, 38.1, 30.8, 29.7, 29.4, 28.8 (3C), 27.2, 22.6, 19.0 (2C), 14.1, 13.7 (2C) ppm; MS (ESI, 30 eV): m/z 813.94 $[M+Na]^+$, 791.70 $[M+H]^+$.

3.3.10.7 Deprotection of intermediate **72** – PAT analog **8**

A cold (0 °C) solution made up of TFA (1.1 mL), PhSH (0.21 mL, 2.1 mmol) and DCM (1.1 mL) was added to the intermediate **72** (76 mg, 0.096 mmol) and the resulting solution was kept at ambient temperature for 1, whereby completion of deprotection was verified by TLC using the solvent system I as eluent. Evaporation under reduced pressure left a residue which upon addition of diethyl ether and overnight refrigeration yielded a white precipitate. The supernatant liquor was decanted, fresh diethyl ether was added to wash the precipitate, the supernatant liquor was again decanted and the solid residue was dried overnight, under reduced pressure over KOH pellets, to yield pure product **8** ($C_{26}H_{48}N_6O_3$, exact mass: 492.38) as the corresponding tetratrifluoroacetate salt. White solid (76 mg, 83%); RP-HPLC: t_R = 2.611 min; MS (ESI, 30 eV): m/z 515.90 $[M+Na]^+$, 493.89 $[M+H]^+$. Anal. Calcd (%) for $C_{29}H_{44}F_9N_5O_9$: C, 43.04; H, 5.52; N, 8.86. Found: C, 43.33; H, 5.28; N, 8.59.

4. Table S1: Structures and antiproliferative activity (IC_{50} values) for tested compounds

Table S1. Structures[@] and antiproliferative activity of synthesized analogs of polyamine toxins from spider and wasp venoms in breast cancer cell lines

Structure	IC_{50} (μM) MCF-7	MDA-MB-231
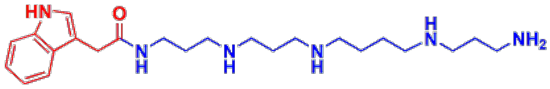 Agel 416	0.55±0.02	3.31±0.08
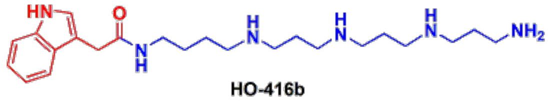 HO-416b	0.09±0.012	3.98±0.064
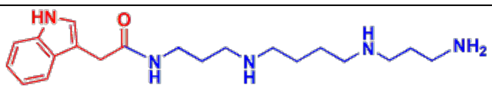 1	>200	>200
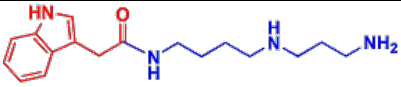 2	3.15± 0.25	12.6± 0.57
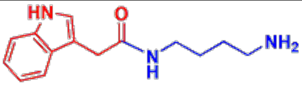 3	272.4±11.2	107.2±10.7
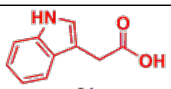 21	365.9±73	262.2±42
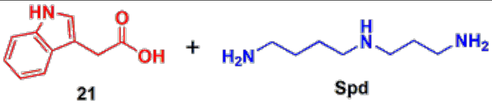 21 Spd 21:Spd=1:1	145.6±96	282.5±55
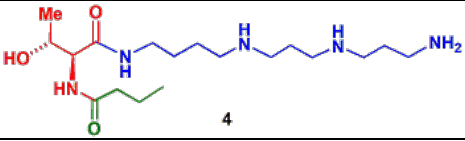 4	>400	>400
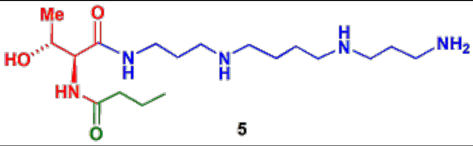 5	>400	>400
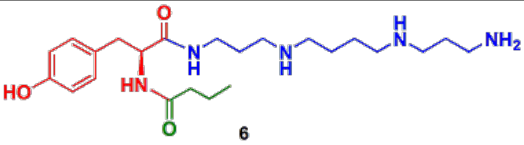 6	>400	>400
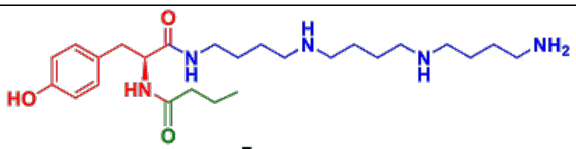 7	>400	>400
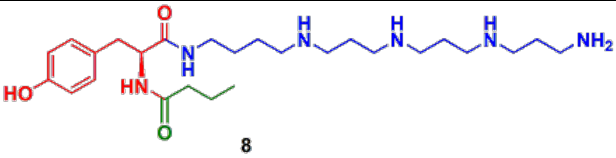 8	>400	>400

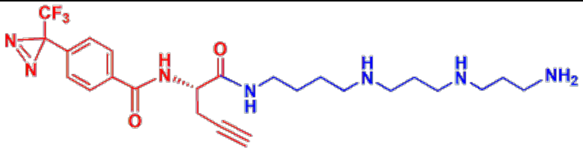 9	**2.63±1.58**	**>200**
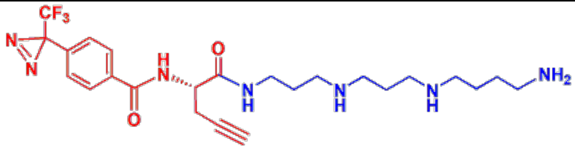 10	**2.81±1.64**	**>200**

@The compounds were isolated,as described in the Experimental Protocols part of the Supplementary Material, and tested as their corresponding polytrifluoroacetate salts as follows: compounds Agel 416 and HO-416b as the corrresponding pentatrifluoroacetates; compounds **1** and **8** as the corresponding tetratrifluoroacetates; compounds **2** and **4-7** and **9-10** as the corresponding tritrifluoroacetate salts; compound **3** as the corresponding ditrifluoroacetate salt.

5. Figure S1: Viability of MCF-12A cells after treatment with PATs HO-416b and Agel 416.

Note: The values in the second table below are the corresponding viability ± standard deviation (SD) values.

	100 µM	10 µM	1 µM	0.1 µM
**HO-416b**	50.80	77.45	89.68	92.45
**Agel 416**	69.02	63.54	84.30	95.42

6.46	6.91	11.50	10.2
2.45	10.03	3.03	8.54

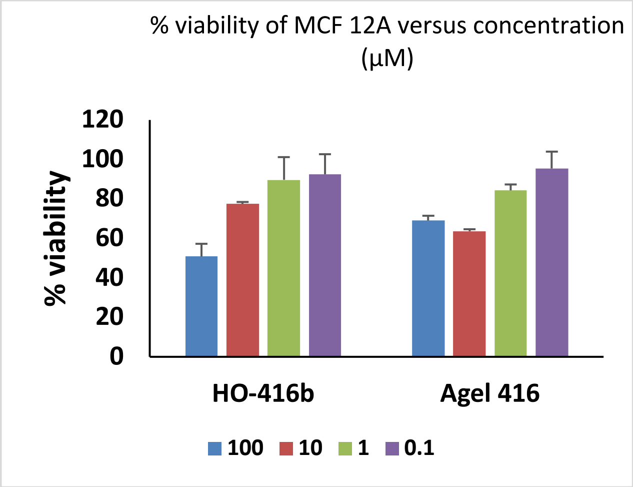

6. Table S2: Structures and IC_{50} values for the effect of PATs Agel 416 and HO-416b on the viability of MCF-12A cells

Table S2. The IC_{50} values of PATs Agel 416 and HO-416b in the MCF-12A epithelial breast cells

Compound	IC_{50} (μM)
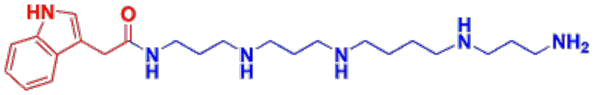 Agel 416	**184.14**
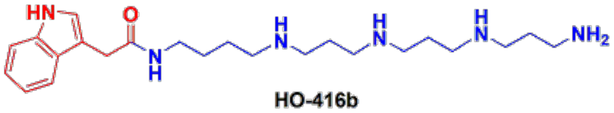 HO-416b	**97.24**
